# Spin‐State Engineering of Iron Phthalocyanine D‐Orbitals via Atomic Fe‐N_4_ Coupling for Enhanced Oxygen Reduction Reaction

**DOI:** 10.1002/advs.202510306

**Published:** 2025-07-18

**Authors:** Ze Lv, Zheng Shu, Yang Qiu, Jiawei Luo, Kaibing Xu, Yimeng Ma, Linping Zhang, Hong Xu, Zhiping Mao

**Affiliations:** ^1^ National Engineering Research Center for Dyeing and Finishing of Textiles College of Chemistry and Chemical Engineering Donghua University No.2999 North Renmin Road Shanghai 201620 China; ^2^ Shanghai Frontier Science Research Center for Modern Textiles Donghua University No.2999 North Renmin Road Shanghai 201620 China; ^3^ Joint Key Laboratory of the Ministry of Education Institute of Applied Physics and Materials Engineering University of Macau Macao SAR 999078 China; ^4^ School of Chemical Engineering Sichuan University Chengdu 610065 China; ^5^ State Key Laboratory for Modification of Chemical Fibers and Polymer Materials College of Materials Science and Engineering Donghua University Shanghai 201620 China

**Keywords:** FePc, oxygen reduction reactive, single atom catalyst, spin‐orbit coupling, Zn‐air battery

## Abstract

Since the properties of electron transfer and orbital interactions in oxygen electrocatalysts are highly spin‐dependent, reaction kinetics and thermodynamics are very sensitive to the spin configuration. However, understanding the spin‐related origin of catalytic activity in heterogeneous molecular electrocatalysts still remains challenging. Herein, a molecular‐atomic coupled catalyst is constructed by integrating iron phthalocyanine (FePc) molecules with Fe‐N_4_ atomic sites anchored on nitrogen‐doped carbon nanotubes (FePc‐Fe‐NCNT). The strong electronic coupling between FePc and the Fe‐N_4_‐containing carbon substrate triggers a transition of the Fe sites from a low‐spin state to an intermediate‐spin state. Additionally, the formation of σ* bonds between the electron‐injected perpendicular d_z2_ orbitals of intermediate‐spin Fe and the 2p orbitals of adsorbed oxygen species suppresses site blocking and accelerates OH* desorption, thereby enhancing the reaction kinetics of the oxygen reduction reaction (ORR). The resulting catalyst exhibits exceptional ORR activity in alkaline media, reaching a half‐wave potential of 0.89 V and negligible degradation after 10,000 cycles. Remarkably, the quasi‐solid‐state Zinc‐air battery based on this prepared catalyst operates stably from −40 to 70 °C with minimal performance loss. This work reveals a spin‐state manipulation strategy for the development of advanced molecular catalysts and provides new insights into the regulation of electronic structure for energy conversion technologies.

## Graphical abstract

We propose a strategy of coupling FePc to atomically dispersed Fe‐N_4_ sites, and design a composite catalyst (FePc‐Fe‐NCNT) that can synergistically tune the 3d electronic configuration of Fe. This strategy triggers a low‐spin to intermediate‐spin transition at Fe centers, balancing the adsorption and dissociation of oxygen‐containing intermediates. The tailored spin‐state coupling simultaneously weakens Fe‐O bond strength and promotes OH* desorption, thereby balancing thermodynamic and kinetic pathways. The resulting catalyst achieves exceptional ORR performance (E_1/2_ = 0.89 V vs. RHE) and stability in alkaline media.

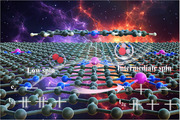

## 1 Introduction

The future of sustainable energy development needs innovative break thoughts in the design of non‐expensive and durable oxygen reduction reaction (ORR) catalysts for efficient metal‐air batteries. However, the ORR process is hindered by the high energy barrier associated with O─O bond dissociation and the sluggish kinetics of the multielectron process.^[^ ^1^ ^]^ While platinum (Pt) and Pt‐based materials have been applied as cathodic electrocatalysts, their applications are limited by high cost and low stability. Therefore, there is an urgent need to replace Pt with alternative materials without compromising the catalytic activity for the ORR.

Atomically dispersed M─N─C catalysts (M═Fe, Co, Ni, etc.) have attracted considerable attention due to their earth‐abundant resources, high atomic utilization, and diverse coordination structures. The inherent unoccupied d orbitals in M‐N‐C catalysts can facilitate efficient electron acceptance from the oxygenated intermediates.^[^ ^2^ ^]^ Among these systems, iron phthalocyanine (FePc) with well‐defined Fe‐N_4_ coordination stands out for its exceptional intrinsic ORR activity.^[^ ^3^ ^]^ However, pure FePc is hindered by its limited electrical conductivity, thus necessitating integration with conductive substrates such as carbon nanotubes (CNTs) or graphene oxide (GO).^[^ ^4^ ^]^ Although some FePc‐based catalysts on carbon materials exhibit high activity,^[^ ^5^ ^]^ there are still stability issues associated with these hybrid constructs. Furthermore, while Fe‐N‐C catalysts occupy the optimal positions on ORR volcano plots,^[^ ^6^ ^]^ their performance is hampered by two intrinsic limitations: (1) over‐absorption of oxygen‐containing intermediates leads to high overpotentials; and (2) H_2_O_2_ produced by the associative pathway attacks both the Fe‐N_4_ active sites and the carbon substrate. These two challenges emphasize the necessity of developing catalysts with robust metal‐support interactions (MSIs) and enhancing their ORR reaction kinetics for practical applications.

It is well established that ORR kinetics involve multiple proton–coupled electron transfer (PCET) steps.^[^ ^7^ ^]^ More specifically, the interaction between metal atoms and O_2_/oxygen‐containing intermediates (e.g., *OOH, *O, *OH) with the electron donation from O species and back‐donation to the metal sites, generally involves spin‐related electron transfer from the paramagnetic O_2_ (triplet ground state) to the diamagnetic intermediates.^[^ ^8^ ^]^ Importantly, the various orbital interactions and electron transfer between the reactant/intermediate and the metal sites strongly influence the adsorption configurations and the adsorption/desorption processes of the intermediates, thereby immensely manipulating both the reaction pathway (associative or dissociative) and the overall reaction kinetics.^[^ ^9^ ^]^ Thus, ORR performance depends on these orbital interactions, which are highly sensitive to the spin configurations of the active metal sites. Consequently, modulating the spin state emerges as a promising strategy for enhancing ORR performance. For instance, Wang *et al.* ^[^ ^10^ ^]^ demonstrated that an axial Fe─O─Ti ligand‐induced transition from low‐spin to intermediate‐spin could promote the filling of Fe eg orbitals to enhance their interactions with the π anti‐bonding orbitals of oxygen, thereby ultimately improving the oxygen affinity and the activity of the ORR. Moreover, Sun *et al.* ^[^ ^11^ ^]^ reported that adjusting the axial ligand field strength reorganizes the Fe 3d orbitals into a high‐spin state, thus enhancing ORR activity at Fe‐N_4_ sites. Collectively, these findings, along with recent reviews by Wang *et al.* ^[^ ^12^ ^]^ and Zhang *et al.*,^[^ ^13^ ^]^ emphasize the crucial role of spin‐state transitions in improving both the ORR performance and stability of Fe‐N‐C catalysts. However, although spin modulation can optimize the electronic structure of active sites, achieving precise and effective control over the spin state during the oxygen reduction reaction remains a significant challenge. This uncertainty continues to impede the rational design of high‐performance ORR catalysts. Moreover, while current theoretical models primarily address the thermodynamics of intermediate adsorption and desorption, the effects of orbital hybridization and spin modulation on ORR kinetics are less explored. Therefore, an effective strategy for the controlled regulation of the spin state at active sites is urgently needed.

Herein, we report a strategy of fabricating the molecular FePc catalysts with atomically dispersed Fe‐N_4_ sites to tailor the electronic structure and spin‐state coupling during the ORR, which can enhance the ORR performance of metal‐air batteries in alkaline media. We found that the FePc‐Fe‐NCNT composite catalyst achieves a half‐wave potential (E_1/2_) of 0.89 V, surpassing the 0.85 V of the 20% Pt/C catalyst, with negligible activity decay after 10,000 CV cycles. Both the experimental and theoretical results indicate that the enhanced performance is due to strong electronic interactions between the FePc and Fe single atoms, which trigger a transition in the spin state of Fe sites from low‐spin (LS) to intermediate‐spin (MS). This improved spin state balances the adsorption and dissociation of oxygen‐containing intermediates, thereby optimizing the reaction thermodynamics of the ORR. Moreover, the formation of σ* bonds between the electron‐injected perpendicular d_z2_ orbitals of intermediate‐spin Fe and the 2p orbitals of adsorbed oxygen species suppresses site blocking and accelerates OH* desorption, thus facilitating the overall ORR kinetics process. More importantly, FePc‐Fe‐NCNT demonstrates excellent cycling stability in quasi‐solid‐state metal‐air batteries, significantly outperforming commercial Pt/C+RuO_2_ and most previously reported ORR catalysts. Furthermore, the temperature adaptability of FePc‐Fe‐NCNT in quasi‐solid‐state Zinc‐Air Batteries (ZABs) was validated over a wide temperature range from −40 to 70°C. This work provides new insights into the structure‐activity relationship from the perspective of spintronic and offers a novel paradigm for the regulation and optimization of catalysts in electrochemical energy technologies.

## 2 Results and Discussion

### 2.1 Synthesis and characterization of FePc‐Fe‐NCNT catalyst

**Figure 1:**
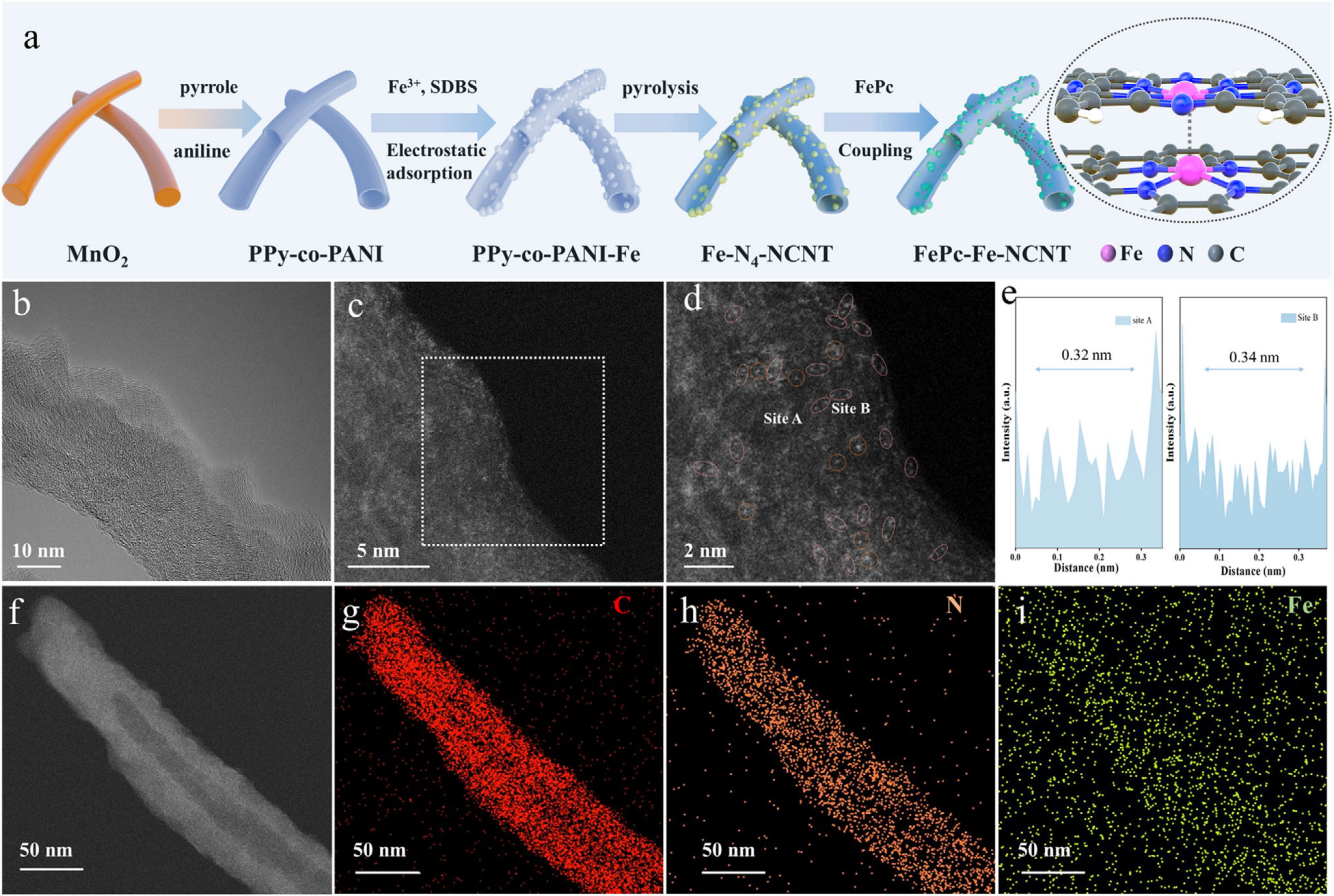
Synthesis scheme and morphology structural characterization of FePc‐Fe‐NCNT. a) Schematic illustration of the synthesis of FePc‐Fe‐NCNT; b) TEM image of FePc‐Fe‐NCNT; c) AC HADDF‐STEM image and d) Enlarged intensity image of FePc‐Fe‐NCNT; e) Line‐scanning intensity profiles obtained from site A and site B are highlighted in d. f–i) Elemental mapping images and corresponding STEM image of C, N, and Fe.

**Figure** **1**a provided an overview of the preparation of FePc‐Fe‐NCNT catalysts. Initially, uniform MnO₂ nanowires were synthesized to serve as both templates and oxidants for the subsequent polymerization of pyrrole and aniline. The higher redox potential of MnO₂/Mn^2^⁺ (1.224 V vs. SHE) compared to that of pyrrole/aniline (0.5 V vs. SHE) drove the spontaneous polymerization, resulting in nitrogen‐rich carbon‐based polymers (PPy‐co‐PANI) hollow structures on the MnO₂ surface.^[^ ^14^ ^]^ To further modify the surface properties, sodium dodecylbenzenesulfonate (SDBS) – an anionic surfactant with both hydrophilic and hydrophobic groups‐was employed to adjust the surface charge and wettability of PPy‐co‐PANI. Following SDBS modification, the PPy‐co‐PANI surface became more hydrophilic, which enhanced electrostatic interactions and facilitated the efficient deposition of Fe^3^⁺ species. Subsequent simultaneous carbonization and reduction under an Ar atmosphere produced Fe‐N_4_‐NCNT. Finally, FePc molecules were anchored onto the Fe‐N_4_‐NCNT substrate via robust non‐covalent π–π stacking and electrostatic interactions,^[^ ^15^ ^]^ forming the composite material denoted as FePc‐Fe‐NCNT. Notably, this coupling process preserved the nanotubular morphology and structure, as confirmed by Scanning electron microscopy (SEM) analysis (Figure S1, Supporting Information). A final thermal treatment at 400 °C in Ar further improved the structural stability of the composite catalyst.

FePc‐Fe‐NCNT exhibited a distinct 1D hollow nitrogen‐doped tubular structure with an inner diameter of ≈30 nm and an outer wall thickness of ≈15 nm (Figure 1b). Transmission electron microscopy (TEM) images (Figure 1b) confirmed the absence of Fe particles or clusters. Moreover, the aberration‐corrected high‐angle annular dark‐field scanning transmission electron microscopy (HAADF‐STEM) image revealed dense bright spots distinct from the carbon nanotube matrix (Figure 1c). Notably, the magnified HAADF‐STEM of FePc‐Fe‐NCNT clearly showed paired bright spots near isolated bright spots, which could be attributed to the dual atom spots pairs (Figure 1d). The measured interatomic distance of ≈0.34 nm between two Fe atoms agrees well with the theoretical model (Figure 1e). Meanwhile, well‐dispersed single atoms could also be observed in FePc‐NCNT (Figure S3, Supporting Information). The energy dispersive X‐ray spectroscopy (EDX) elemental mappings further supported the homogeneous distribution of Fe, N, C throughout the entire architecture (Figure 1f–i). Additionally, inductively coupled plasma mass spectrometry (ICP‐MS) analysis determined that the Fe content in FePc‐Fe‐NCNT was 1.5%. As controls, N‐doped carbon (NCNT) without Fe‐N_4_ and its coupling hybrid (FePc‐NCNT) were synthesized by similar methods. SEM and TEM images of the FePc‐NCNT and NCNT (Figure S4, Supporting Information) also displayed a characteristic nanotubular structure, similar to that of FePc‐Fe‐NCNT.

X‐ray diffraction patterns (XRD) of all catalysts showed only one broad peak at 25 °, corresponding to (002) crystal faces of graphitized carbon (Figure S9, Supporting Information), suggesting the absence of aggregated FePc molecules or crystalline Fe particles. Additionally, in order to further eliminate the influence for catalytic activity of carbon structure differences among the different catalysts, the Raman spectroscopy for all as‐synthesized catalysts were tested. As shown in Figure S9 (Supporting Information), the similar I_D_/I_G_ value of FePc‐Fe‐NCNT and other catalysts proved similar disordered or defective carbon structures for above catalysts. Fourier–transform infrared spectroscopy (FT‐IR) identifies the peak at 1331, 1287, 1165, 1119, and 1079 cm^−1^, which represent the C = N, C = C, C─N, C─H and C─C vibrations of FePc moieties. FT─IR spectrum of FePc‐Fe‐NCNT displays signals from FePc (728, 1119, and 1331 cm^−1^).^[^ ^16^ ^]^ Upon coupling with Fe‐N carbon materials, the first peak of out‐of‐plane vibrations exhibits a significant blue shift (Figure S10, Supporting Information). This result suggests that Fe‐N can induce strong out‐of‐plane interactions with FePc. Consequently, the electronic structure of the active sites in Fe‐N_4_ undergoes reconstruction, leading to a reduction in the reaction energy barrier and an enhancement in ORR activity.^[^ ^17^ ^]^ The N₂ adsorption‐desorption isotherms (Figure S11, Supporting Information) indicate that FePc‐Fe‐NCNT possesses a relatively large specific surface area of 150 m^2^ g^−1^, with abundant micropores and mesopores, which are conducive to the transport of O_2_ and facilitate the ORR process.

**Figure 2:**
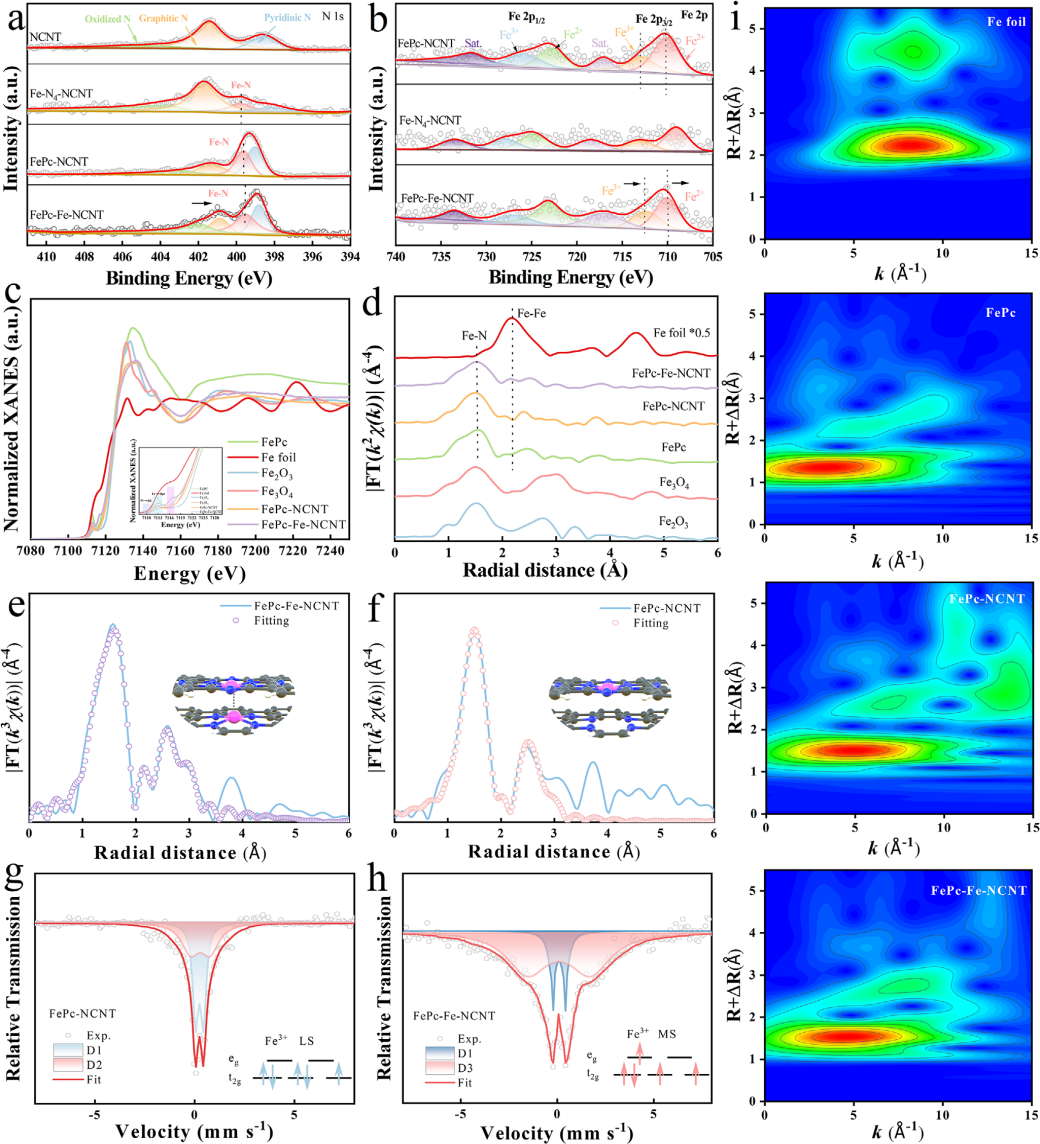
Valence state analysis and coordination environment of FePc‐Fe‐NCNT a) High‐resolution N 1s spectra and b) Fe 2p spectra; c) Fe K‐edge XANES spectra; d) Fourier transformed k3‐weighted Fe K‐edge of EXAFS spectra; Fe FT‐EXAFS fitting curves of e) FePc‐Fe‐NCNT and f) FePc‐NCNT, the inset exhibit the model of the Fe environment; 57Fe Mössbauer spectrum, and their deconvolution g) of FePc‐NCNT and h) FePc‐Fe‐NCNT; i) Wavelet transform for the K^3^‐weighted EXAFS signals of Fe foil, FePc, FePc‐NCNT and FePc‐Fe‐NCNT.

X‐ray photoelectron spectroscopy (XPS) analysis was performed to determine the chemical composition, bonding, and valence states of the elements. The XPS survey spectra of NCNT, Fe‐N_4_‐NCNT, FePc‐NCNT, FePc‐Fe‐NCNT, and FePc illustrate the presence of C, N, and Fe (Figures S15–S19, Supporting Information). The high‐resolution N 1s spectrum of FePc presents two main types of N species, pyridinic N (399.1 eV) and pyrrolic N (400.2 eV) (Figure S19, Supporting Information). Interestingly, the primary N type in FePc‐NCNT is pyridinic N, while the main type in FePc‐Fe‐NCNT is graphitic N and pyridinic N, which suggests the possibility of a different local environment in the two configurations.^[^ ^18^ ^]^ Moreover, the observed peak splitting in the high‐resolution N 1s XPS spectra of both FePc‐NCNT and FePc‐Fe‐NCNT indicates strong interactions between FePc and the NCNT support (**Figure** **2**a),^[^ ^19^ ^]^ which may further suppress the dissolution of FePc in the electrolyte. The high‐resolution spectrum of Fe 2p_3/2_ from FePc‐Fe‐NCNT shows two peaks at 712.4 and 727.3 eV, which can be assigned to the 2p_3/2_ and 2p_1/2_ of Fe^3+^,^[^ ^20^ ^]^ while peaks at 710.1 and 723.1 eV are attributed to Fe^2^⁺ oxidation states (Figure 2b).^[^ ^21^ ^]^ Additionally, the main peak was accompanied by two satellite peaks. Compared to FePc‐NCNT, the Fe 2p_3/2_ spectrum of FePc‐Fe‐NCNT shows a negative shift, indicating that some electrons have transferred from Fe to FePc. Conversely, when compared to Fe‐N_4_‐NCNT, the Fe 2p_3/2_ spectrum of FePc‐Fe‐NCNT shows a positive shift, suggesting electron transfer from FePc to Fe. This bidirectional electronic coupling confirms interfacial charge redistribution. This interfacial charge redistribution modulates 3d orbital occupancy, as evidenced by enhanced satellite peak intensity and a larger spin‐orbit splitting in FePc‐Fe‐NCNT (Δ₂p = 12.8 eV vs. 12.4 eV for FePc‐NCNT), which indicates a higher number of unpaired 3d electrons.^[^ ^22^ ^]^ This phenomenon is attributed to electronic interactions between FePc and Fe that facilitate d‐orbital hybridization and mediate spin‐state transitions at the Fe active site.^[^ ^23^ ^]^ These electronic reconfigurations optimize the adsorption energy of oxygenated intermediates, thereby enhancing ORR activity.

X‐ray absorption spectroscopy (XAS) was performed to elucidate the local electronic environment and electronic structure of Fe in FePc‐Fe‐NCNT. As shown in Fe K‐edge X‐ray Absorption Near Edge Structure (XANES) spectra (Figure 2c), the absorption edge of FePc‐Fe‐NCNT shifts to lower energies compared with FePc‐NCNT, indicating a reduced oxidation state. Furthermore, the white‐line peak (corresponding to 1s → 3d transition) of FePc‐Fe‐NCNT is slightly increased compared to FePc‐NCNT, indicating a higher density of unpaired 3d electrons, probably due to the strong interaction between FePc and the substrate Fe‐N_4_‐NCNT.^[^ ^17^ ^]^ Density function theory (DFT) calculations were also conducted to evaluate the binding energy of FePc with the two substrates, confirming that FePc‐Fe‐NCNT has a higher binding energy (Figure S20, Supporting Information). Moreover, the pre‐edge peak at ≈7113.5 eV can be attributed to the 1s → 4p_z_ transition in the symmetric Fe‐N_4_ structure of FePc.^[^ ^24^ ^]^ The observed decrease in pre‐edge peak intensity for both FePc‐Fe‐NCNT and FePc‐NCNT indicates a disruption of the D_4h_ symmetry in the square‐planar coordination.^[^ ^3^ ^]^ In addition, a new shoulder peak appears at 7116.5 eV in FePc‐Fe‐NCNT, indicating stress‐induced distortions at the Fe‐N_4_ site leading to the disruption of the planar symmetry of Fe‐N coordination. Subsequently, the linear relationship between the XANES absorption threshold and the oxidation state of the standard samples was utilized to further investigate the electronic structure of the FePc and Fe atoms (Figure S21, Supporting Information). Notably, the oxidation state of Fe in FePc‐NCNT is higher than that in isolated FePc, suggesting electron density depletion in the FePc molecules.^[^ ^10^ ^]^ In contrast, Fe in FePc‐Fe‐NCNT exhibits a lower oxidation state than in FePc‐NCNT. These results demonstrate that incorporating Fe‐N_4_ coordination effectively accelerates electron transfer and modulates the local electronic environment. The synergistic effects between FePc and the Fe‐N_4_‐NCNT support facilitate charge polarization and drive electron transfer from the Fe‐N_4_‐NCNT to the Fe centers in FePc.

Figure 2d presented the Fourier Transform k^3^‐weighted Extended X‐ray Absorption Fine Structure (FT‐EXAFS) spectra, which were conducted to gain deeper insights into the coordination environment of the Fe site. Both FePc‐NCNT and FePc‐Fe‐NCNT exhibit a prominent main peak at ≈1.5 Å, corresponding to first‐shell Fe‐N coordination.^[^ ^25^ ^]^ Compared to FePc, the Fe─N bond length in FePc‐Fe‐NCNT is shorter, indicating a distortion in the spatial chemical environment of the Fe sites. This bond length reduction is primarily attributed to the introduction of Fe‐N_4_, which enhances the strong electronic interactions between FePc and Fe‐N_4_. Consequently, this enhancement leads to a reshaping of the 3d orbital occupation at the active site,^[^ ^26^ ^]^ resulting in a change of spin state.^[^ ^27^ ^]^ Moreover, the thermal treatment process may contribute to the reduced bond length by inducing molecular deformation. To further unveil the precise coordination geometry, FT‐EXAFS fitting analysis of the Fe‐K edge (Figure 2e,f) demonstrates that the first coordination shell of FePc‐Fe‐NCNT can be assigned to the Fe‐N_4_ scattering path. Moreover, a mixture of Fe‐N, Fe‐C, and Fe‐Fe coordination paths are identified in the second coordination shell of FePc‐Fe‐NCNT, and an interlayer spacing of 3.48 Å between the axial FePc and the planar Fe‐N_4_‐NCNT is observed. Similarly, the K‐edge EXAFS spectra of Fe‐N_4_‐NCNT in Figure S26 (Supporting Information) show that the prominent peak at 1.50 Å is due to Fe‐N coordination, indicating that the Fe atoms are atomically distributed in the nitrogen‐doped carbon nanotube. Wavelet transform (WT)‐EXAFS analysis was performed in k‐space, overcoming the limitations in R‐space, to identify the backscattering atoms. The WT‐EXAFS analysis (Figure 2i) exhibits only one major intensity maximum at ≈4.9 Å for the FePc samples, further confirming the existence of the Fe─N bonding. The position of Fe‐N intensity maximum for FePc‐Fe‐NCNT (4.7 Å) is lower than that of FePc‐Fe‐NCNT (4.9 Å), indicating the structural change of the local electronic behavior due to the manipulation of strong electronic interactions. This structural modification could further optimize its ORR activity. Both FT and WT‐EXAFS analysis prove that the Fe atom is isolated, which consistent with the HADDF‐STEM results. Overall, these XAFS analyses reveal that charge transfer between FePc and Fe‐N_4_ sites modulates the d‐band electronic structure of Fe, which tailors the adsorption energetics of oxygen intermediates.

^57^Fe Mössbauer spectroscopy was further employed distinguish Fe species with analogous coordinated environments but different electron configurations.^[^ ^28^ ^]^ As depicted in Figure 2g, the Mössbauer spectra of FePc‐NCNT were fitted to two doublet peaks (D1 and D2), corresponding to the isomer shift (IS) and quadrupole splitting (QS) values. These can be assigned to low‐spin Fe(III) (S = 1/2) and low‐spin Fe(II) (S = 1/2),^[^ ^27^ ^]^ respectively. In the case of FePc‐Fe‐NCNT (Figure 2h), the nearly identical isomer shifts values of the two observed doublets (D1 and D3) indicate minimal differences in s‐electron density at the Fe nuclei. Notably, the observed quadrupole splitting difference is caused by the electric field gradient (EFG) asymmetry of the Fe nucleus, reflecting the d‐electron redistribution of FePc‐Fe‐NCNT. Quantitative analysis of the FePc‐NCNT spectrum reveals that D1 and D2 contribute 51.2% and 48.8% to the total spectral area, respectively (Table S4, Supporting Information), reflecting a mixture of low‐spin Fe(II) and Fe(III) states within the Fe‐N_4_ moieties. After coupling with Fe‐N_4_‐NCNT, the intensity of D1 decreases significantly, and a new doublet, D3, becomes dominant with a relative area of 74.9% in FePc‐Fe‐NCNT, indicating the predominance of intermediate‐spin Fe(III) states.^[^ ^7a^ ^]^ Furthermore, a smaller isomer shift of the spectrum of FePc‐Fe‐NCNT compared to FePc‐NCNT shows an enhanced shielding effect of Fe‐3d electrons on 1s electrons at the core position due to delocalization. These findings confirm that coupling with Fe‐N_4_‐NCNT increases the electron density in the Fe‐N_4_ moiety, generating more unpaired electrons. The strong interactions between the single Fe atom and FePc effectively remodel the electronic structure of Fe, inducing a transition in the Fe 3d electron spin configuration from low‐spin (LS) to intermediate‐spin (MS), which ultimately confers high ORR catalytic activity to FePc‐Fe‐NCNT.

### 2.2 Electrocatalytic Evaluation

**Figure 3:**
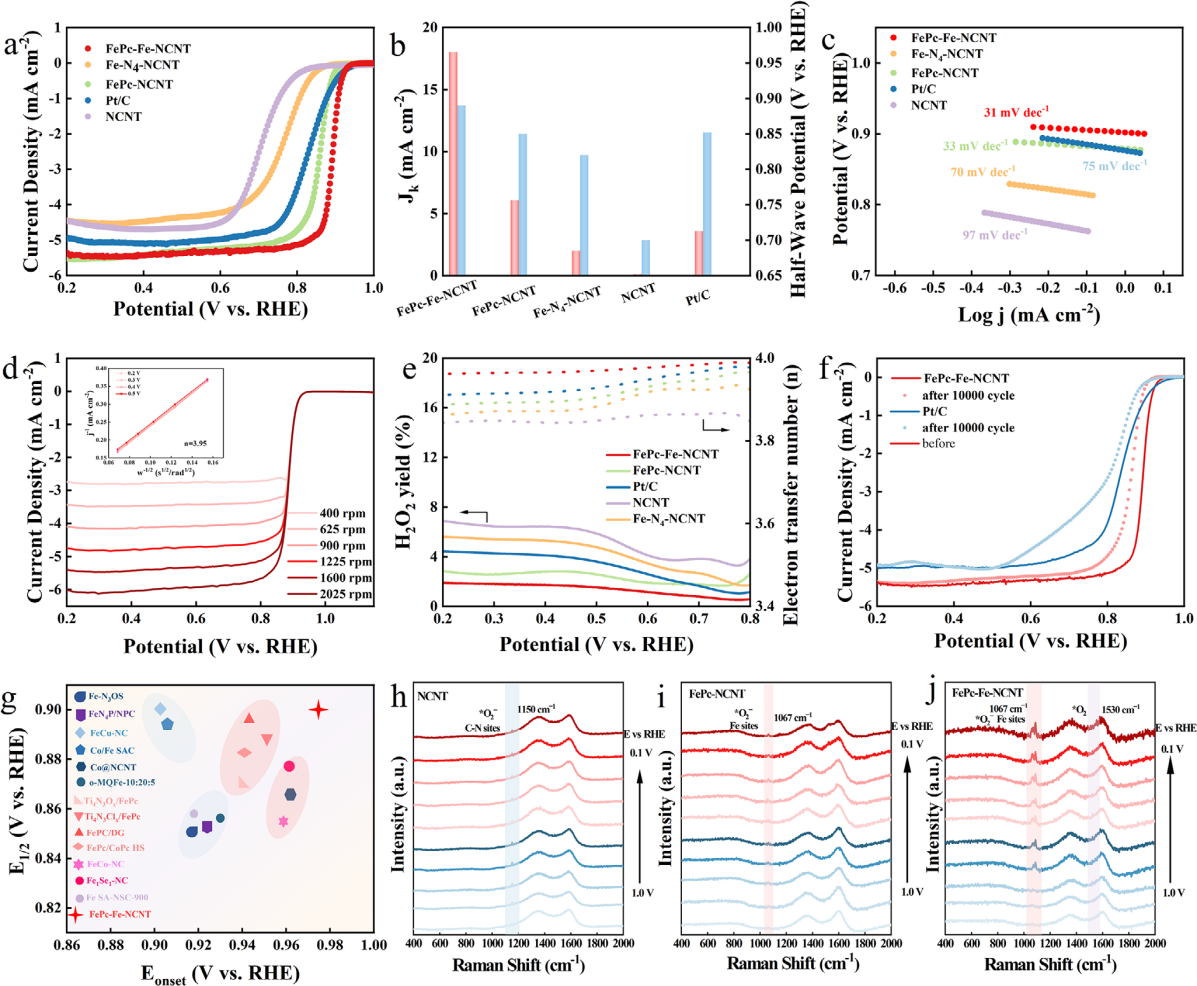
ORR performance a) ORR polarization curves of FePc‐Fe‐NCNT and reference catalysts; b) E_1/2_ and J_k_ of FePc‐Fe‐NCNT and reference catalysts; c) Tafel slop of FePc‐Fe‐NCT and reference catalysts d) LSV curves of FePc‐Fe‐NCNT obtained at different rotating speeds, corresponding fitted K‐L plots of FePc‐Fe‐NCNT; e) H_2_O_2_ yield and electron transfer number from RRDE polarization curves for FePc‐Fe‐NCNT and reference catalysts; f) ORR polarization curves after 10000 cycles of FePc‐Fe‐NCNT and 20% Pt/C; g) Comparison of alkaline ORR performance for FePc‐Fe‐NCNT with reported catalysts; In situ Raman spectra of h) NCNT, i) FePc‐NCNT and j) FePc‐Fe‐NCNT catalyst at different potentials vs. RHE in a three‐electrode system.

The oxygen reduction reaction activity was evaluated using ring‐disk electrode modified with FePc‐Fe‐NCNT, FePc‐NCNT, Fe‐N_4‐_NCNT, NCNT, and 20% Pt/C in oxygen‐saturated 0.1 M KOH solutions. The potential was calibrated to a reversible hydrogen electrode (RHE). As shown in **Figure** **3**a, FePc‐Fe‐NCNT delivers a significantly enhanced ORR activity with a high half‐wave potential (E_1/2_) of 0.89 V, superior to those of FePc‐NCNT (0.852 V) and Fe‐N_4_‐NCNT (0.80 V). In contrast, the metal‐free NCNT (0.78 V) shows poor electrocatalytic activity. Notably, the ORR activity of FePc‐Fe‐NCNT is among the best for non‐precious metal electrocatalysts. In order to quantitatively assess the ORR activity, the kinetic current density (J_k_) was calculated based on the LSV curves in Figure 3b, the J_k_ at 0.85 V of FePc‐Fe‐NCNT is 18.03 mA cm^−2^, which is higher than that of FePc‐NCNT (6.06 mA cm^−2^) and Pt/C (3.6 mA cm^−2^), indicating its higher intrinsic activity. Remarkably, FePc‐Fe‐NCNT exhibits a minimum Tafel slope of 31 mV dec^−1^, indicating its fastest reaction kinetics arising from the strong electronic interactions between FePc and Fe‐N_4_‐NCNT (Figure 3c). Further analysis revealed a turnover frequency (TOF) for FePc‐Fe‐NCNT of 0.58 e s^−1^ site^−1^, significantly higher than those recorded for FePc‐NCNT and 20% Pt/C (Table S5, Supporting Information). Additionally, as depicted in Figure S33 (Supporting Information), the electrochemical double‐layer capacitance (C_dl_) of FePc‐Fe‐NCNT is calculated to be 123.9 mF cm^−2^, larger than those of FePc‐NCNT (78.08 mF cm^−2^), Fe‐N_4_‐NCNT (61.16 mF cm^−2^), and NCNT (48.91 mF cm^−2^), revealing that FePc‐Fe‐NCNT possesses a larger electrochemical active surface area. The OER polarization curves of serial catalysts are compared in Figure S34 (Supporting Information). The FePC‐Fe‐NCNT possesses the over potential of 330 mV at 10 mA cm^−2^ apparently lower than those of RuO_2_ (350 mV), and FePc‐ NCNT (370 mV). Correspondingly, FePc‐Fe‐NCNT also exhibits a Tafel slope (85.4 mV dec^−1^) comparable to that of RuO₂ (94.6 mV dec^−1^), illustrating its fast kinetics in the OER process.

The electron transfer number (n), which was crucial for determining the ORR pathway and applicable scenarios, was investigated using different rotation speeds and rotating ring‐disc electrode (RRDE) techniques as well as Koutecky–Levich plots (Figure 3d,e). As shown in Figure 3d, the current density of the catalyst was obviously enhanced with the increase in rotational speed. The value of n (3.98) supports the occurrence of an ideal four‐electron transfer process, where O_2_ is reduced to H_2_O. Furthermore, the linearity of the K–L plots suggests that the FePc‐Fe‐NCNT catalyst exhibits first‐order kinetics in ORR. Additionally, the K–L plot of FePc‐NCNT exhibited an electron transfer number of 3.9, while the carbon matrix achieved a value of 3.8 (Figure S35, Supporting Information). To further validate these results, the selectivity of the catalyst in ORR was analyzed using RRDE measurements. This showed that FePc‐Fe‐NCNT had the lowest H_2_O_2_ yield and the highest electron transfer number (n = 3.98) (Figure 3e), confirming a strong preference for the four‐electron pathway from O_2_ to H_2_O. This performance is clearly superior to that of FePc‐NCNT, both in terms of electron transfer number and H_2_O_2_ production. Moreover, electrochemical impedance spectroscopy (EIS) results showed that FePc‐Fe‐NCNT exhibits lower charge transfer resistance (Figure S36, Supporting Information). Overall, these findings suggest that the incorporation of the Fe‐N_4_‐NCNT significantly enhances the ORR kinetics of FePc‐Fe‐NCNT.

The significantly lower hydrogen peroxide yield observed in FePc‐Fe‐NCNT indicates a more efficient four‐electron ORR pathway, thereby contributing to enhanced catalyst stability. By minimizing the formation of reactive hydroxyl and hydroperoxyl radicals through Fenton side reactions, the risk of catalyst degradation during prolonged ORR cycles is reduced.^[^ ^11^ ^]^ This improved stability was further validated through CH_3_OH resistance and accelerated durability tests (ADT). Notably, the current attenuation of FePc‐Fe‐NCNT after methanol injection into the electrolyte is negligible, demonstrating excellent methanol resistance (Figure S37, Supporting Information). This resistance is primarily attributed to the absence of active sites for methanol oxidation, suggesting a broad range of potential applications. Furthermore, as shown in Figure S38 (Supporting Information), after 40000 s of chronoamperometry at 0.9 V, the current retention of FePc‐Fe‐NCNT is 98.5%, which is higher than that of Pt/C (87.08%). Additionally, FePc‐Fe‐NCNT exhibited excellent stability after 10000 CV cycles between 0.6 and 1.1 V versus RHE, with a half‐wave potential drop of only 8.9 mV. In comparison, a larger shift of 28.4 mV was observed for 20% Pt/C (Figure 3f). These results confirm that the Fe–N_4_ sites on the NCNT matrix effectively anchor the FePc molecules, thereby inhibiting their aggregation during prolonged cycling. In turn, the anchored FePc molecules help to prevent the leaching of Fe‐N_4_ sites from the NCNT matrix. The superior stability of FePc‐Fe‐NCNT further supports the beneficial effects of spin‐state modulation on catalyst durability. TEM and EDS mapping of FePc‐Fe‐NCNT after cycling clearly show that its morphology and structural properties are well preserved (Figures S39 and S40, Supporting Information). XPS analysis provides insights into the electronic structure of Fe species in the catalyst after the accelerated durability test process. As depicted in Figure S41 (Supporting Information), there was almost no change in the valence state of Fe following extended operation at a reductive potential. The analysis of both geometric and electronic structural features confirms the stability of the Fe‐N sites even after prolonged aging under ADT conditions. To the best of our knowledge, the ORR performance of FePc‐Fe‐NCNT exceeded that of most other reported Fe‐N_4_‐based catalysts, as detailed in Figure 3g and Table S6 (Supporting Information).

In situ Raman spectroscopy was applied to compare the catalytic activity of FePc‐Fe‐NCNT and FePc‐NCNT, by which the dynamic change of oxygen intermediates in active sites was detected under varied potentials during the ORR in 0.1 M KOH electrolyte. The spectrum of FePc‐Fe‐NCNT electrocatalyst exhibited two peaks ≈ 1607 cm^−1^ and 1350 cm^−1^ corresponding to G‐band and D‐band, respectively. The value of I_D_/I_G_ did not change significantly during the test, indicating that the FePc‐Fe‐NCNT electrocatalyst has high structural stability. As reported that the frequencies ranging from 1000 cm^−1^‐1200 cm^−1^ are assigned to active O─O^−^ species (*O_2_^−^) in an alkaline electrolyte.^[^ ^29^ ^]^ In Figure 3g, only one peak at 1150 cm^−1^ is observed in the Raman spectra of the iron‐free NC catalyst, attributed to *O_2_^−^ absorbed on C‐N sites. Another peak at 1067 cm^−1^ is observed in the Raman spectra of FePc‐NCNT and FePc‐Fe‐NCNT is attributed to *O_2_^−^ adsorbed on Fe‐N_4_ sites. The peak attenuation in FePc‐NCNT is ascribed to the weakened in plane Fe‐N_4_ stretching vibration at a low potential. The Fe atom shifts away from the original in‐plane Fe‐N_4_ structure owing to the adsorption of *O_2_^−^ species as the potential decreases, which generates a nonplanar *O_2_^−^−FeNC configuration. By contrast, the favorable structural stability of Fe‐N_4_ in FePc‐Fe‐NCNT can withstand the dynamic geometric change under low potentials. Compared to that of FePc‐NCNT, the intensity of Raman peaks at 1060 cm^−1^ in FePc‐Fe‐NCNT is strengthened, further confirming its enhanced ORR activity.^[^ ^4a^ ^]^ The peak at 1530 cm^−1^ corresponds to the O‐O vibration of surface‐adsorbed *O_2_.^[^ ^30^ ^]^ These findings provide valuable insights into the ORR mechanism of FePc‐Fe‐NCNT.

### 2.3 Exploration of Spin States of Fe Single Atom And Catalytic Mechanism

**Figure 4:**
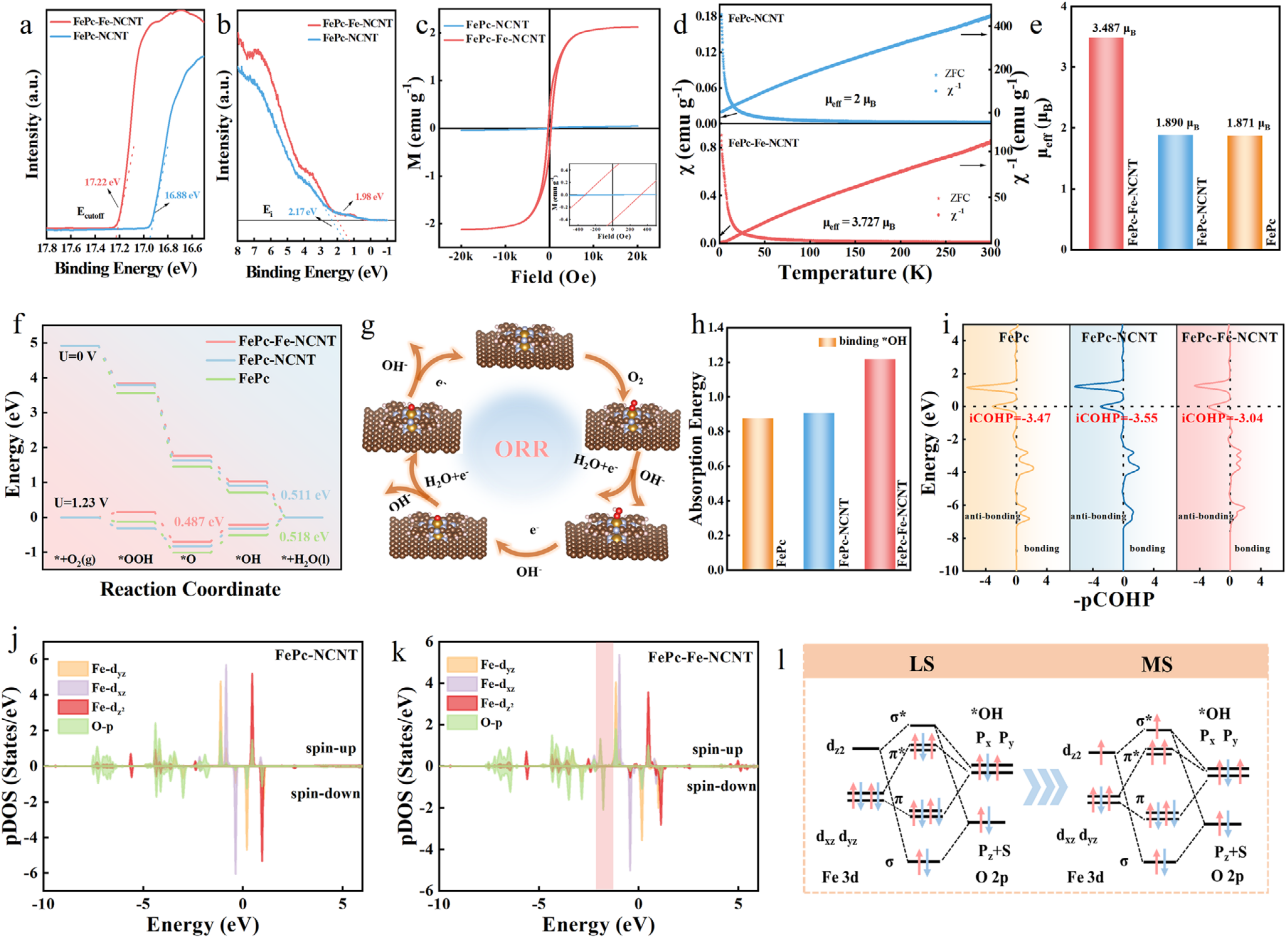
Mechanistic investigation. a) Valence‐band spectrum of FePc‐Fe‐NCNT and FePc‐NCNT; b) The work functions of FePc‐Fe‐NCNT and FePc‐NCNT (right); c) Magnetic hysteresis (M–H) loops of FePc‐Fe‐NCNT and FePc‐NCNT; d) Temperature‐dependent magnetic susceptibility χ_m_(T) and reciprocal χ_m_ of FePc‐NCNT and FePc‐Fe‐NCNT; e) the corresponding magnetic moment (µ_eff_); f) Free energy diagram of ORR for FePc‐Fe‐NCNT, FePc‐NCNT and FePc at 1.23 V and 0 V vs. RHE; g) ORR process on the Fe‐N_4_ site of FePc‐Fe‐NCNT; h) *OH absorption energies on the optimized model structures of FePc, FePc‐NCNT and FePc‐Fe‐NCNT; i) The projected crystal orbital Hamilton populations (pCOHP) demonstrate the Fe‐OH bonding strength in FePc, FePc‐NCNT and FePc‐Fe‐NCNT, respectively; Projected density of states (pDOS) of Fe and O over FePc‐NCNT j) and FePc‐Fe‐NCNT k); l) speculated orbital interaction between low spin/ intermediate spin Fe^3+^ (d) and *OH (p).

To elucidate the origin of the FePc‐Fe‐NCNT catalyst's high ORR activity, a spin‐orbit coupling perspective has been proposed. To be more specific, UV photoemission spectroscopy (UPS) was engaged to illustrate the energy level differences (**Figure** **4**a), the cutoff energy (E_cutoff_) energy of FePc‐Fe‐NCNT and FePc‐NCNT was 17.12 and 16.88 eV, respectively. According to the equation Φ = 21.22 eV − E_cutoff_, their work function was calculated to be 4.00 and 4.34 eV (Figure 4b), indicating that less energy is required for FePc‐Fe‐NCNT to transfer electrons to oxygen‐containing intermediates, thereby facilitating electron transfer. In addition, the energy of the valence band maximum (E_v_) for FePc‐Fe‐NCNT and FePc‐NCNT was determined to be 1.98 and 2.17 eV, respectively, suggesting that the introduction of Fe‐N_4_ leads to an increase in the iron 3d electron density and a decrease in its oxidation state,^[^ ^7a^ ^]^ consistent with XPS and XANES results. In general, the E_v_ reflects changes in the electron arrangement of Fe influenced by the transition of the 3d electron spin configuration.^[^ ^31^ ^]^ Thus, to further clarify the electronic structure of FePc‐Fe‐NCNT, vibrating sample magnetometry (VSM) measurements were performed at room temperature. Figure 4c illustrates that the exhibited saturation magnetization (Ms) increases from 0.03 emu g^−1^ to 2.1 emu g^−1^. Moreover, an enlarged view of the curve is depicted in the inset of Figure 4c, suggesting that FePc‐Fe‐NCNT exhibits a coercive magnetic field (Hc) of 321.2 Oe and a residual magnetization (Mr) of 0.42 emu g⁻^1^, both values exceeding those of FePc‐NCNT. This indicates that the introduction of Fe‐N_4_ increases the number of unpaired electrons, thereby enhancing the spin state.^[^ ^32^ ^]^ These magnetic property changes are attributed to significant alterations in the material's electronic structure. Further evaluation through zero‐field cooling (ZFC) temperature‐dependent magnetic susceptibility measurements quantified unpaired electrons and revealed their electron spin configuration.^[^ ^33^ ^]^ The effective magnetic moment (µ_eff_) is related to the number of unpaired electrons (n) by the equation:

(1) $$\mu_{\mathrm{eff}}=\sqrt{n\;\times \left(n+2\right)}$$

As shown in Figure 4d, both catalysts were found to be paramagnetic, with FePc‐Fe‐NCNT exhibiting a larger effective magnetic moment (*µ*_eff_ = 3.727 µ_B_) than FePc‐NCNT (µ_eff_ = 2 µ_B_). Moreover, the number of unpaired electrons of Fe is verified to be 2.86 for FePc‐Fe‐NCNT, exceeding that of the FePc‐NCNT (1.23). Owing to this larger effective magnetic moment and the higher number of unpaired electrons, the orbital interactions between FePc and Fe‐N_4_ redistribute the electronic structure of the active site Fe, triggering a transition from a low‐spin (LS, t_2g_^5^e_g_^0^) state to an intermediate‐spin (MS, t_2g_^4^e_g_^1^) state.^[^ ^34^ ^]^ By projecting the electron wave function onto the occupied orbitals, we calculated the effective magnetic moments (Figure 4e) to be 3.487 µ_B_ for FePc‐Fe‐NCNT and 1.89 µ_B_ for FePc‐NCNT. In contrast, the effective magnetic moment of FePc‐NCNT is comparable to that of FePc, which is attributed to the strong‐field ligands generated by the phthalocyanine macrocycle in FePc that induce a low‐spin state at the Fe center.^[^ ^35^ ^]^ Importantly, the spin‐state effect can also be visualized through the spin density diagrams (Figure S42, Supporting Information), in which heightened spin states generate broader spin‐dependent channels. This spatially extended spin polarization enhances orbital overlap between the Fe d_Z_^2^ orbitals and the 2p orbitals of adsorbed oxygen species, thereby promoting electronic interaction with ORR intermediates and facilitating their desorption. These features are consistent with the assignment of an intermediate‐spin state (t₂_g_^4^e_g_^1^), as supported by experimental VSM and Mössbauer data. Furthermore, the transition of the spin state of Fe 3d is also supported by ^57^Fe Mössbauer spectra.

Previous studies have revealed that the spin state of Fe center is highly related to the kinetics and thermodynamics of ORR. Subsequently, the Gibbs free energy evolutions of ORR through the associative mechanism on FePc, FePc‐NCNT and FePc‐Fe‐NCNT were calculated to investigate their activity. The 4*e* ^−^ ORR proton‐coupled electron transfer mechanism can be summarized as: (I) the adsorption and hydrogenation of O_2_ on the catalytic site to form the intermediate *OOH; (II) one proton attacks *OOH to break the O─O bond, resulting in the cleavage of O─O bond and H_2_O release, thus forming the intermediate *O; (III) the protonation of *O to form the intermediate *OH; and (IV) *OH removal by the combing one proton and the intermediate *OH.^[^ ^36^ ^]^ Thus, the Gibbs free energy diagrams of ORR process according to this mechanism on FePc, FePc‐NCNT and FePc‐Fe‐NCNT at *U* = 0 V and *U* = 1.23 V are shown in Figure 4f. We can observe that all catalysts are thermodynamically spontaneous at *U* = 0 V vs RHE. As indicated by the rate‐determining step (RDS) at equilibrium potential *U* = 1.23 V, the overpotential of FePc‐NCNT (0.518 V) is almost the same as that of molecular FePc (0.511 V), indicating that it is difficult to change energy barrier of RDS (*OH + H^+^ + *e* ^−^→ * + H_2_O) by NCNT substrate. For the FePc‐Fe‐NCNT, the third PCET step of OOH*+ H^+^ + *e* ^−^→ O*+H_2_O is the RDS with an overpotential of 0.487 V, which is lower than those of FePc and FePc‐NCNT. To simulate this process more thoroughly, the DFT+U method is considered to correct the free energy diagram. As shown in Figure S43 (Supporting Information), a larger ΔG difference of RDS between FePc‐NCNT and FePc‐Fe‐NCNT is observed after considering DFT+U approach and show the similar tendency with the results of previous calcualtions. These results suggest that the Fe‐N_4_ mainly acts as an activity booster to optimize the self‐assembled electronic configuration of the FePc and modulate adsorption and desorption energies of the intermediates. Therefore, the introduction of Fe dopants into NCNT substrate could inhibit the dissolution of FePc and reduce the energy barrier of RDS, thus achieving highly efficient ORR activity. The optimized structures of FePc‐Fe‐NCNT with *OOH, *O and *OH intermediates during ORR are shown in Figure 4g. To determine the impact of introducing Fe‐N_4_ coordination on the electronic configuration of FePc‐Fe‐NCNT orbitals, density of states (DOS) calculations was performed. Figures S44–S46 (Supporting Information) present the five 3d orbitals (d_xy_, d_xz_, d_yz_, d_x2–y2_ and d_z2_) for FePc‐Fe‐NCNT, FePc‐NCNT, and FePc, based on crystal field theory. Notably, the d_z2_ and d_yz_ orbitals in FePc‐Fe‐NCNT exhibit a pronounced shift toward the Fermi level, indicating enhanced conductivity and more facile electron transitions. Furthermore, the observed orbital redistribution provides convincing evidence for strong electronic interactions between the FePc moiety and the Fe‐N_4_ site.^[^ ^37^ ^]^

To elucidate the origin of overpotential reduction on FePc‐Fe‐NCNT, the adsorption free energies of *OH intermediates on FePc, FePc‐NCNT and FePc‐Fe‐NCNT are first examined, as shown in Figure 4h. It is revealed that the *OH adsorption free energy of FePc‐Fe‐NCNT is 1.22 eV, which is weaker than those of FePc (0.88 eV) and FePc‐NCNT (0.91 eV). This characteristic could decrease the free energy change of key step and accelerate the desorption of oxygen intermediates, thus facilitating the overpotential reduction of ORR. In addition, the crystal overlap Hamilton population (COHP) method was employed to further explain the bonding interaction of Fe center and O atom in *OH intermediates. As illustrated in Figure 4i, the negative value of integrated COHP (−iCOHP) of FePc‐Fe‐NCNT is the smallest (−3.04 eV), implying its weakest Fe‐O interaction among three catalysts. In other two cases, Fe−O interaction is stronger than that of FePc‐Fe‐NCNT, as revealed by their more negative −iCOHP values (−3.47 and −3.55 eV) of Fe─O bonding, which is consistent with previous results of *OH adsorption free energies. To elucidate the weaked binding strength for *OH intermediates on FePc‐Fe‐NCNT, the projected density of states (PDOS) of three *OH‐adsorbed systems were calculated for comparison, as shown in Figure S47 and Figure 4j,k (Supporting Information). The orbitals of Fe d_xz_, d_yz_, and d_z2_ hybirdize with O─p orbital, filling the 𝜋* orbital of O to form Fe─O bond when adsorbs OH. This result indicated that the partially occupied d‐orbitals of Fe could donate back electrons to the O‐p orbital. Furthermore, it is found that the FePc‐Fe‐NCNT exhibits a fewer overlapped ratio between Fe 3d and O 2p orbitals compared with the case of FePc‐NCNT. As shown in Figure 4k, there exists an additional bonding state of O−p orbital, which is not hybirdized with Fe 3d orbitals (marked by shaded area). Therefore, it can explain the weakest interaction between Fe center and *OH intermediates on FePc‐Fe‐NCNT. Above analysis demonstrated that FePc‐Fe‐NCNT could modulate the binding strength of OH intermediates, making the charged states of Fe^𝛿+^ less positive to optimize ORR kinetics. To further elucidate the relationship between the spin state of Fe atoms and the adsorption strength of oxygenated intermediates, the atomic orbital bonding schematic was employed to reveal the underlying mechanism. The role of Fe d_xy_ and d _x2–y2_ orbitals can be neglected due to symmetry conservation principle.^[^ ^8^ ^]^ As illustrated of Fe 3d electron configurations (d_xz_, d_yz_, and d_z2_ orbitals) with low‐spin state and intermediate‐spin in Figure 4l, the electrons preferentially occupy lower hybirdized orbitals when *OH intermediates couple with low‐spin Fe atoms, thus resulting in a stronger bonding interaction and enhanced molecular adsorption capability. In contrast, the electrons are filled into σ^*^ antibonding orbitals when intermediate‐spin Fe atoms bond with the 2p electrons of *OH, which can weaken the strength of Fe─O bond and accelerates the ORR kinetics process. Based on above results, the improved ORR performance of FePc‐Fe‐NCNT can be attributed to the enhanced spin state of Fe center and reduced Fe−O interaction.

### 2.4 Electrochemical Evaluation of Zn‐Air Battery

**Figure 5:**
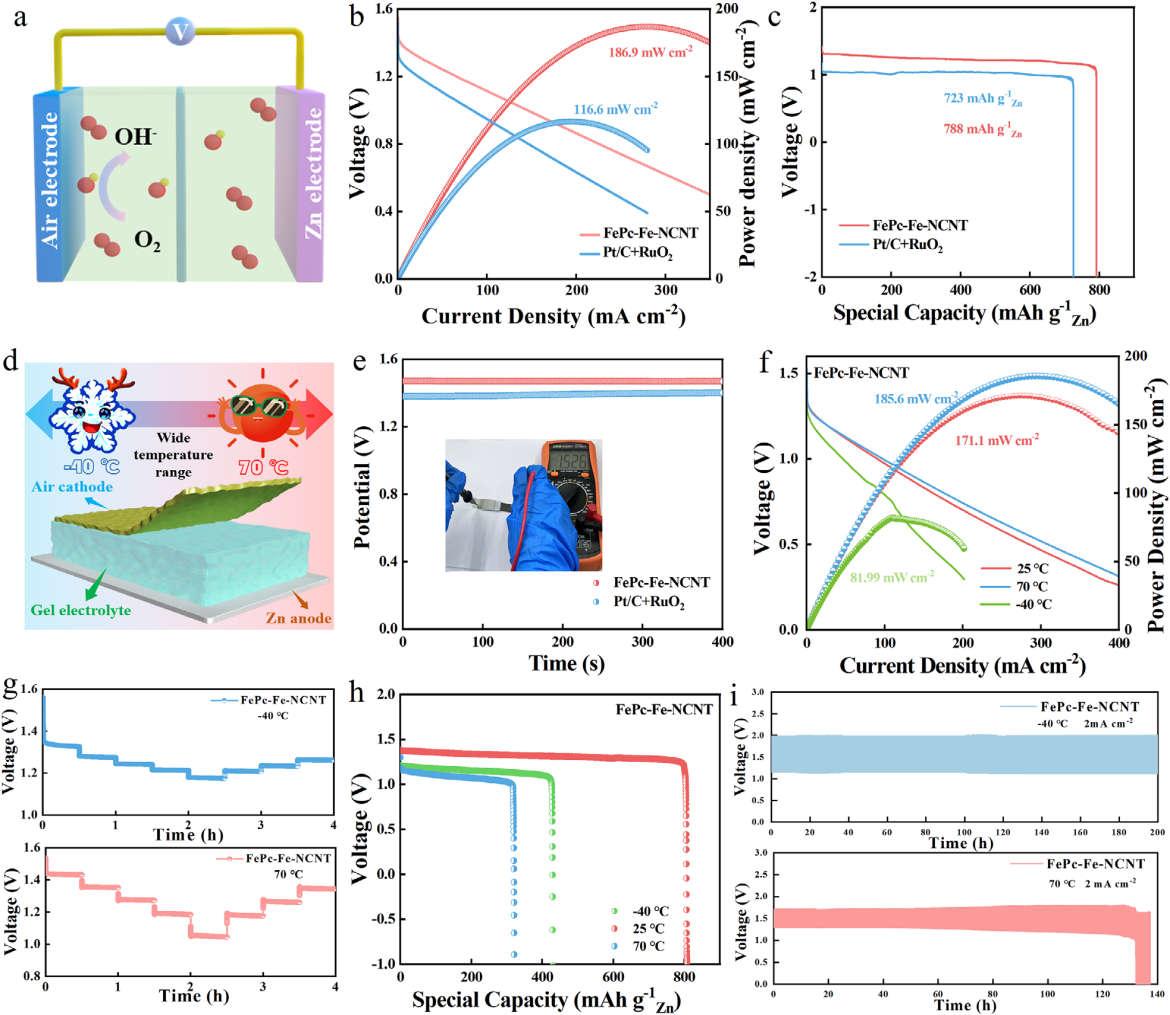
Zn‐air batteries performance. a) Schematic representation of rechargeable liquid Zn‐air battery; b) Discharge polarization curves and corresponding power density curves; c) Galvanostatic discharge curves at a current density of 10 mA cm^−2^; d) Schematic illustration of temperature‐adaptive quasi‐solid‐state ZABs; e) Open‐circuit potential (OCP) curves of quasi‐solid‐state Zn‐air batteries assembled with FePc‐Fe‐NCNT and Pt/C+RuO_2_; f) Discharge polarization plots and corresponding power density curves at −40, 25, 70 °C; g) Galvanostatic discharge curves at various current densities from 1 to 10 mA cm^−2^; h) Galvanostatic discharge curves at 5 mA cm^−2^ of FePc‐Fe‐NCNT based QSS‐ZAB under −40, 25, 70 °C; i) The charge and discharge cyclic performance at 2 mA cm^−2^ and −40, 70 °C.

Owing to its superior ORR activity and stability, the FePc‐Fe‐NCNT catalyst was utilized as the cathode material in rechargeable liquid Zn–air batteries (ZABs) (**Figure** **5**a). The open‐circuit voltage of ZABs with FePc‐Fe‐NCNT was 1.57 V (Figure S48, Supporting Information), surpassing the 1.44 V observed for Pt/C+RuO_2_ cathodes. Notably, the FePc‐Fe‐NCNT‐based ZABs delivered an impressive peak power density of 180 mW cm^−2^ at 313.4 mA cm^−2^, significantly outperforming the Pt/C+RuO_2_ cathode (116.6 mW cm^−2^ at 279.6 mA cm^−2^) (Figure 5b). Discharge performance analysis revealed a specific capacity of 788 mAh g⁻^1^ for FePc‐Fe‐NCNT, exceeding the 723 mAh g⁻^1^ value of Pt/C+RuO_2_ (Figure 5c). This comprehensive enhancement can be attributed to two synergistic mechanisms: atomic‐level tuning of the Fe–N_4_ active sites, which improves intrinsic catalytic activity, and the incorporation of hierarchical carbon nanotube networks that optimize charge transfer at the electrode–electrolyte interfaces. Moreover, as shown in Figure S49 (Supporting Information), When the current density increased from 1 to 10 mA cm⁻^2^, FePc‐Fe‐NCNT‐based ZABs exhibited only a 4.5% discharge potential drop, compared to the 5.3% decline observed for Pt/C+RuO_2_. Consistently, the FePc‐Fe‐NCNT cathode maintained superior discharge plateaus across varying current densities, highlighting its robust oxygen reduction kinetics and rate adaptability. Finally, two FePc‐Fe‐NCNT‐based ZABs connected in series were capable of powering a 3 V LED display (Figure S50, Supporting Information), thereby demonstrating the catalyst's potential for practical applications.

The development of energy storage systems capable of operating across broad temperature ranges remains critical for practical implementations.^[^ ^38^ ^]^ Among various electrolyte candidates, polyacrylamide (PAM) solid‐state electrolytes demonstrate particular promise due to their exceptional elastic moduli and mechanical strength.^[^ ^39^ ^]^ Consequently, a rechargeable Quasi‐Solid‐State ZAB (QSS‐ZAB) is further assembled, utilizing FePc‐Fe‐NCNT as the air cathode, polished Zn foil as the air anode, and a polyacrylamide‐dimethyl‐sulfoxide (PAM‐DMSO) hydrogel electrolyte. Remarkably, the FePc‐Fe‐NCNT‐based QSS‐ZAB achieved a stable open‐circuit voltage of 1.45 V at room temperature, surpassing the 1.40 V performance of conventional Pt/C+RuO_2_‐based counterparts (Figure 5e). The enhanced performance was further evidenced by discharge polarization tests, where the FePc‐Fe‐NCNT system demonstrated a peak power density of 185.6 mW cm^−2^ higher than the Pt/C+RuO_2_‐based device (106.55 mW cm^−2^) (Figure S52, Supporting Information). Cycling stability tests revealed superior charge/discharge durability compared to the Pt/C+RuO_2_ reference system (Figure S53, Supporting Information), while bending tests confirmed stable operation at various angles (Figure S54, Supporting Information). This mechanical resilience is attributed to the optimized viscoelastic properties of the PAM‐DMSO electrolyte, which preserves structural integrity through dynamic hydrogen‐bonded networks. Furthermore, the FePc‐Fe‐NCNT‐based QSS‐ZAB demonstrated exceptional power density compared to both Pt/C+RuO_2_ and state‐of‐the‐art catalysts reported in recent studies (Table S7, Supporting Information).

The temperature adaptability of QSS‐ZABs was systematically evaluated across extreme conditions (−40 to 70 °C). Notably, the FePc‐Fe‐NCNT‐based QSS‐ZAB demonstrated remarkable thermal stability, achieving peak power densities of 171.7 mW cm⁻^2^ at 70 °C and 81.99 mW cm^−2^ at −40 °C. Correspondingly, as shown in Figure 5f, the FePc‐Fe‐NCNT‐based QSS‐ZABs delivered a specific capacity of 470 mAh g_Zn_^−1^ at 70 °C and 370 mAh g_Zn_^−1^ at −40 °C. This enhanced performance can be attributed to the hierarchically porous nitrogen‐doped carbon nanotube architecture of FePc‐Fe‐NCNT, which simultaneously exposes abundant accessible active sites, shortens ion diffusion paths, and particularly critical for maintaining reaction kinetics under low‐temperature stress. Furthermore, the PAM‐DMSO gel electrolyte, in contrast to conventional liquid electrolytes, maintains superior ion conductivity at low temperatures, enhancing the low‐temperature adaptability of the QSS‐ZABs. This synergistic combination enabled stable rate performance during current density cycling (1–10 mA cm^−2^), confirming the catalyst's excellent temperature adaptability across this wide range (Figure 5g). Long‐term cycling tests at 2 mA cm^−2^ revealed exceptional durability, as shown in Figure 5i, the FePc‐Fe‐NCNT‐based QSS‐ZABs exhibited a discharging voltage of 1.26 V and a charging voltage of 1.95 V, and could stably operate for 200 hours at −40 °C. Benefiting from the good stability of the FePc‐Fe‐NCNT catalyst, the QSS‐ZABs maintained a stable discharging voltage after 120 h of charge/discharge cycling at 70 °C, with an initial discharging voltage of 1.28 V and a charging voltage of 1.8 V. Such comprehensive performance establishes FePc‐Fe‐NCNT as a thermally robust catalyst platform for extreme‐condition energy devices.

## 3 Conclusion

In summary, we present a new class of hybrid oxygen electrocatalysts composed of molecular FePc and individual Fe atoms with well‐regulated spin states, designed to enhance the oxygen reduction reaction (ORR) performance in alkaline media. We demonstrate that spin regulation can ameliorate the relatively slow ORR kinetics of Fe‐N‐C catalysts. The electronic interaction between FePc and Fe‐N_4_ effectively facilitates the filling of the d_z2_ orbitals of Fe in the FePc‐Fe‐NCNT, leading to a transition from a low‐spin state to an intermediate‐spin state, further strengthening the interaction between FePc and the substrate as well. The FePc‐Fe‐NCNT catalyst exhibits excellent ORR activity, with an E_1/2_ of 0.89 V, and outstanding stability, showing almost no degradation after 10,000 cycles. The study reveals that the occupied d_z2_ orbital alleviates the over‐adsorption of the key intermediate *OH at the active sites, thereby shifting the ORR overpotential toward the volcano peak. When FePc‐Fe‐NCNT is used as the cathode in a quasi‐solid‐state metal‐air battery, it achieves a power density of 185.6 mW cm^−2^ at 25 °C and demonstrates long‐term cycling stability. Additionally, the adaptability of FePc‐Fe‐NCNT at both −40 °C low temperature and 70 °C high temperature has been verified. This work provides precise guidance for spin‐regulated FePc‐based oxygen reduction catalysts, with significant prospects for energy storage and conversion applications.

## Conflict of Interest

The authors declare no conflict of interest.

## Supporting information



Supporting Information

## Data Availability

The data that support the findings of this study are available from the corresponding author upon reasonable request.
