# Supplementary material for: Spin‐State Engineering of Iron Phthalocyanine D‐Orbitals via Atomic Fe‐N4 Coupling for Enhanced Oxygen Reduction Reaction
Source: Adv Sci (Weinh). 2025 Jul 18;12(37):e10306. doi: 10.1002/advs.202510306 (PMC12499505; doi:10.1002/advs.202510306)
Supplement: Supplementary file 1 — Supporting Information [file ADVS-12-e10306-s001.docx]

**Spin-State Engineering of Iron Phthalocyanine d-Orbitals via Atomic Fe-N_4_ Coupling for Enhanced Oxygen Reduction Reaction**

Ze Lv ^a, #^, Zheng Shu ^c, #^, Yang Qiu ^d^, Jiawei Luo^a^, Kaibing Xu ^e^, Yimeng Ma^a^, Linping Zhang ^a^, Hong Xu^a^, Zhiping Mao ^a, b *^

1. *National Engineering Research Center for Dyeing and Finishing of Textiles, College of Chemistry and Chemical Engineering, Donghua University*

*No.2999 North Renmin Road, Shanghai, 201620, China*

1. *Shanghai Frontier Science Research Center for Modern Textiles, Donghua University*

*No.2999 North Renmin Road, Shanghai, 201620, China*

1. *Joint Key Laboratory of the Ministry of Education, Institute of Applied Physics and Materials Engineering University of Macau, Macao SAR 999078, China*
2. *School of Chemical Engineering, Sichuan University, Chengdu, 610065, China*
3. *State Key Laboratory for Modification of Chemical Fibers and Polymer Materials, College of Materials Science and Engineering, Donghua University, Shanghai 201620, China*

#These authors contributed equally to this work.

* Corresponding authors.

**Contents**

**S1 Experimental Details**

**S2 Computational Details**

**S3 Supplementary Figures and tables**

**S4 References**

**S1 Experimental Details**.

**S1.1 Material preparation**

**Preparation of MnO_2_:** Firstly, 3 mmol of MnSO_4_, 2 mmol of KMnO_4_, and 2 mmol of NH_4_F were dissolved in 40 mL of deionized water under magnetic stirring to form a homogeneous solution. The solution was then transferred to a 50 mL Teflon-lined stainless steel autoclave, which was subsequently placed in a convection oven at 150°C for 12 hours. After the autoclave naturally cooled to room temperature, the brown product was collected.

**Preparation of PPy-co-PANI**: In synthesis of PPy-co-PANI, 200 mg of MnO_2_ NWs were dispersed in 200 mL of 0.02 M H_2_SO_4_. Subsequently, 510 µL of C_4_H_5_N and 390 µL of C_6_H_7_N were added to the solution. After stirring for 6 hours, the product was collected by centrifugation using deionized water, followed by drying in an 80°C oven for 24 hours.

**Preparation of Fe-N_4_-NCNT**: In synthesis of Fe-N_4_-NCNT, First, 100 mg of sodium dodecylbenzene sulfonate (SDBS) and 400 mg of PPy-co-PANI were dispersed in 20 mL of deionized water under continuous stirring for 6 hours. Subsequently, 86.54 mg of FeCl_3_·6H_2_O) was added to the above mixture, followed by stirring for an additional 24 hours. The resulting precipitate was collected via centrifugation, thoroughly washed with deionized water and ethanol to remove residual impurities, and dried under vacuum at 60°C for 12 hours. Finally, the obtained product was thermally annealed at 1100°C for 1 hour under a high-purity argon flow (heating rate: 5°C/min) to yield the Fe-N_4_-NCNT.

**Preparation of FePc-Fe-NCNT**: In synthesis of FePc-Fe-NCNT, 10 mg of iron phthalocyanine (FePc) was dissolved in 120 mL of DMF and sonicated for 30 minutes. Then, 50 mg of Fe-N₄-NCNT was added, and the mixture was sonicated until homogeneous. The solution was subsequently stirred vigorously for 24 hours. The resulting mixture was filtered and repeatedly washed with ethanol until the filtrate became colorless. The product was then vacuum-dried. The obtained powder was placed in a tubular furnace and heated to 400°C under an argon atmosphere (heating rate: 5°C/min) for 2 hours. After natural cooling, the FePc-Fe-NCNT product was collected.

**S1.2** **Material characterizations**

The structural characteristics of the prepared materials were examined using multiple techniques: scanning electron microscopy (SEM, S-4800) operated at 5 kV, transmission electron microscopy (TEM, JEM-2100/JEM-2100F) at 200 kV/800 kV, and aberration-corrected HADDF-STEM (JEM-ARM300) at 300 kV. X-ray diffraction (XRD) analyses were conducted using a Bruker D8 ADVANCE instrument. Fourier transform infrared (FT-IR) spectroscopy was carried out using a Bruker ALPHA spectrometer with KBr pellets. X-ray photoelectron spectroscopy (XPS) measurements were acquired using an Escalab 250Xi, with all recorded binding energy data calibrated using C 1s (284.8 eV) as the reference to analyze the chemical composition. The Renishaw 2000 model confocal microscopy Raman spectrometer was employed for Raman spectroscopy. The specific surface area was determined by analyzing the N_2_ adsorption/desorption isotherms (Autosorb-iQ) according to the Brunauer-Emmett-Teller (BET) method. The iron content was assessed using an inductively coupled plasma optical emission spectrometer (ICP-OES, Agilent 5110). The temperature magnetic susceptibility (M-T) curves were obtained using a MPMS3 Quantum Design SQUID physical property measurement system with a constant external magnetic field of 1000 Oe in the temperature region of 2 to 300 K. ^57^Fe Mössbauer spectra were performed using an MS-500 instrument (Germany, Wissel) with a ^57^Co (Pd) source at room temperature without magnetic field. The Fe K-edge X-ray absorption spectra (XAS) were recorded at the BL14W1 beamline of the Shanghai Synchrotron Radiation Facility. XAS data were processed and analyzed using the Demeter software package. Data reduction, data analysis, and EXAFS fitting were performed and analyzed with the Athena and Artemis programs of the Demeter data analysis packages ^[1]^ that utilizes the FEFF6 program ^[2]^ to fit the EXAFS data. The Fe K-edge theoretical XANES simulation were carried out with the FDMNES code in the framework of real-space full multiple-scattering (FMS) scheme using Muffin-tin approximation for the potential.^[3]^ The used FePc-NCNT and FePc-Fe-NCNT model were based on the optimized model by DFT calculation.

**S1.3 Electrochemical measurements**

All the electrochemical measurements were conducted in 0.1 mol L^–1^ KOH electrolyte using a three-electrode system operated on an electrochemical workstation (CHI760E). The working electrode were the rotating disk electrode (RDE) with a glass carbon disk of 5 nm diameter and rotating ring-disk electrode (RRDE) with a Pt ring. The graphite rod electrode and Hg/HgO electrode (1 mol L^–1^ KOH solution) were used as a counter electrode and reference electrode. The recorded potential values vs. Hg/HgO were converted to a reversible hydrogen electrode (RHE) scale according to the Nernst equation (E_RHE_ = E_Hg/HgO_ + 0.059 × pH + 0.098). For the working electrode preparation, 5mg catalyst sample were dispersed in 450 μL ethanol 50 μL 5 wt% Nafion by sonication for 1 h. The prepared catalyst ink was coated on the rotating disk electrode with a mass catalyst load of 0.5 mg cm^–2^ followed by air drying.

The cyclic voltammetry (CV) was performed in N_2_ and O_2_-saturated 0.1 M KOH solution with a sweep rate of 50 mV s^–1^. The linear sweep voltammetry (LSV) curves were recorded in O_2_ saturated electrolyte at 5 mV s^–1^ in rotating speed of 1600 rpm. The polarization curve of LSV at a scanning rate of 10 mV s^–1^ was recorded in 0.1 M KOH solution with a rotating speed of 400 rpm ~ 2025 rpm. The H_2_O_2_ yield was obtained by RRDE measurements. The electrochemical active surface area (ESCA) of the catalyst was evaluated by measuring the double layer capacitance at different scanning rates (2 to 10 mV s^−1^) in the non-Faraday potential range using CV curves.

The kinetic parameters of the ORR can be analyzed through the Koutecky - Levich (K-L) plots by

$$\frac{1}{j}=\frac{1}{j_{k}}+ \frac{1}{j_{l}}=\frac{1}{{B\omega}^{\frac{1}{2}}}+\frac{1}{j_{k}} \left( 1 \right)$$

$$B=0.2nFC_{O_{2}}D_{O_{2}}^{\frac{2}{3}} \left( 2 \right)$$

The measured current density is expressed as j, the kinetic current density is j_k_, the diffusion limited current density is j_l_, the electrode speed (rpm) is ω, the electron transfer number of O_2_ is n, the Faraday constant (96485 C mol^−1^) is F, and the volume concentration of O_2_ (7.8×10^−7^ mol cm^−3^) is C_O2_. The kinetic viscosity of the electrolyte (0.01 cm^2^ S^−1^) is υ and the diffusion factor of O_2_ at 0.1 M KOH is 1.8 × 10^−5^ cm^2^ S^−1^.

Hydrogen peroxide yield (H_2_O_2_ %) and electron transfer number (n) are determined by the following formula:

$$\%\left( H2O2 \right)=200\times\frac{\frac{I_{r}}{N}}{I_{d}+\frac{I_{r}}{N}} \left( 3 \right)$$

$$n=4\times\frac{I_{d}}{I_{d}+\frac{I_{r}}{N}} \left( 4 \right)$$

where I_d_ is the disk current, I_r_ is the ring current, and N is the current collection efficiency of the Pt ring (0.37).

The turnover frequency (TOF) and mass activity (MA) of the catalyst are normalized by the total metal content, and the formula is as follows:

$$\mathrm{MA}\left[ A/g \right]=\frac{j_{k}}{m_{0}\times w_{metal}} \left( 5 \right)$$

$$\mathrm{TOF}\left[ S^{-1} \right]=\frac{j_{k}}{n_{metal}\times F}=\frac{M_{metal}\times j_{k}}{m_{metal}\times F}=\frac{M_{metal}\times j_{k}}{m_{0}\times F\times w_{metal}} \left( 6 \right)$$

Where the kinetic current is j_k_, m_0_ is the mass loading of catalysts on glassy carbon electrode surface, w_metal_ is the mass concentration of metal in the catalysts, n_metal_ is the mol amount of metal in the catalysts, m_metal_ is the mass of metal in the catalysts, M_metal_ is the atomic weight of metal, and F (96485 C mol^–1^) is the Faraday constant.

Stability was characterized both by accelerated aging test and CV. The former compared the LSV curves before and after cycling in the potential range of 0.6~1.0 V vs. RHE for 10000 cycles. For the current-time chronoamperometric test, O_2_ was bubbled into 0.1 M KOH electrolyte for 30 min prior to the experiment and a flow of O_2_ was maintained over the electrolyte during the test to ensure O_2_ saturation. The test process was constant at 0.9 V vs. RHE. For the tolerance to CH_3_OH test was carried out by chronoamperometric measurements at 0.9 V vs. reversible hydrogen electrode (RHE) in a O_2_-saturated mixed solution containing 0.1 M KOH (180 mL) along with the injection of CH_3_OH (8 mL) at the time of 300 second.

**S1.4 Zn-air battery assembly and measurements**

A zinc-air battery (ZAB) was assembled with zinc sheet as anode and hydrophobic carbon paper coated with catalyst as cathode. The electrolyte is a mixed aqueous solution of 6 M KOH and 0.2 M Zn(Ac)_2_. Cathode catalytic ink was prepared by dispersing 8mg FePc-Fe-NCNT or Pt/C+RuO_2_ in 800 μL water, 200 μL isopropyl alcohol and 60 μL 5 wt% nafion, ultrasonic for 30 min. The catalyst is coated on the hydrophobic carbon paper as the cathode of the air battery with a load of 1 mg cm^−2^.

**Synthesis of polyacrylamide-dimethyl sulfoxide (PAM-DMSO) hydrogel electrolyte**

The hydrogel electrolyte was prepared as follows: 4 g of acrylamide (AM), 4 mg of N, N′-methylenebisacrylamide (MBAA, Crosslinking agent), and 10 mg of potassium persulfate (K_2_S_2_O_8_, Initiator) were dissolved in 7 mL H_2_O and 3 mL dimethyl sulfoxide under magnetic stirring at 500 r min^–1^. The resultant solution was poured into a transparent rectangular mold, which was then sealed and placed in a 60°C oven for 4 hours. The as-prepared PAM-DMSO hydrogel (thickness of 0.3 cm) was immersed in the 6 M KOH + 0.2 M Zn(Ac)_2_·6H_2_O electrolyte for 72 h before use.

The electrochemical testing of ZAB was conducted in the electrochemical workstation (CHI660E) of the dual electrode system and in the ambient air of the Neware battery measurement system. The LSV curve records the discharge and charging curves of a half ZAB with an air cathode at room temperature. The constant current discharge, rate performance, and charge discharge cycle (10 minutes discharging followed by a 10 minutes charging.) of the battery were recorded on the Neware battery tester.

**S2 Computational Details**

The spin-polarized DFT calculations were performed using the projector-augmented wave (PAW) pseudo-potentials in the Vienna *ab initio* Simulation Package (version 5.4.4) code.^[4, 5]^ The Perdew–Burke–Ernzerhof (PBE) form of the generalized gradient approximation (GGA) was used to depict the exchange-correlation functional with a plane-wave cutoff energy of 450 eV.^[6, 7]^ The structures in ORR process were fully optimized with single Γ point by the conjugate-gradient (CG) algorithm until the total energy and Hellmann-Feynman force are less than 1 × 10^-5^ eV and 0.05 eV Å^–1^, respectively. For static calculations, a 2 × 2 × 1 *k*-point mesh and a denser 3 × 3 × 1 *k*-point mesh within Monkhorst-Pack scheme were used to obtain the system’s total energy and calculate the projected density of states (pDOS), respectively. To avoid the periodic interaction, a ~15 Å vacuum layer was adopted in the *z*-axis, and the van der Waals dispersion correction was considered via the DFT-D3 method.^[8]^ For 3*d* metal Fe, the Hubbard *U*_eff_ correction with a value of 5.30 (consistent with the value of Materials Project) was included to describe the localized *d* electrons. ^[9]^ The binding energies between FePc molecule and NCNT substrate can be calculated by *E*_b_ = *E*_FePc+NCNT_ – *E*_NCNT_ – *E*_FePc_, where *E*_FePc+NCNT_, *E*_NCNT_ and *E*_FePc_ are the energies of NCNT with adsorbed FePc molecule, NCNT without FePc molecule, and FePc molecule, respectively. The crystal orbital Hamilton population (COHP) calculations of the Fe-O bonding characteristics were implemented through the LOBSTER code.^[10]^ All geometric structures were visualized using the VESTA package.

As for ORR in an alkaline electrolyte, four elementary proton-coupled electron transfer (PCET) steps can be proceeded as follows:

$*+O_{2}(g)+H_{2}O\left( l \right)+e^{-}\to OOH^{*}+OH^{-}$ (7)

$\mathrm{OO}H^{*}+e^{-}\to O^{*}+OH^{-}$ (8)

$O^{*}+H_{2}O\left( l \right)+e^{-}\to OH^{*}+OH^{-}$ (9)

$OH^{*}+e^{-}\to OH^{-}+*$ (10)

According to the computational hydrogen electrode (CHE) model proposed by Nørskov *et al.*,^[11]^ the Gibbs free energy change of each PCET step can be calculated as:

$\Delta G=\Delta E+\Delta ZPE-T\Delta S$ (11)

where ∆*E* represents the energy difference of the system before and after elementary reaction, ∆*ZPE* and ∆*S* are the contributions of zero-point energy and entropy change, which can be calculated from vibrational frequencies, and *T* is the temperature which is set to 298.15 K in our work. The calculations of *ZPE* and *TS* are based on the following equations:

$ZPE=\frac{1}{2}\sum_{i} h\nu_{i}$ (12)

$-TS=k_{B}T\sum_{i} \ln\left( 1-e^{-\frac{h\nu_{i}}{k_{B}T}} \right)-\sum_{i} h\nu_{i}\left( \frac{1}{e^{\frac{h\nu_{i}}{k_{B}T}}-1} \right)$ (13)

where *k*_B_, h and *v*_i_ denote Boltzmann constant, Planck constant and vibrational frequencies of mode *i*, respectively. Therefore, the reaction Gibbs free energy of equations (7)-(10) for ORR can be calculated using the following equations:

$\Delta G_{1}=G_{OOH*}-G_{*}-G_{O2}-{1/2G}_{H2}$ (14)

$\Delta G_{2}=G_{O*}+G_{H2O}-G_{OOH*}-{1/2G}_{H2}$ (15)

$\Delta G_{3}=G_{OH*}-G_{O*}-{1/2G}_{H2}$ (16)

$\Delta G_{4}=G_{*}+G_{H2O}-G_{OH*}-{1/2G}_{H2}$ (17)

The Gibbs free energy of the O_2_ molecule should be determined according to *G*_O2_(g) = 2*G*_H2O_(l) − 2*G*_H2_ + 4 × 1.23 (eV) due to the poor description of O_2_ molecule high-spin ground state in DFT calculations. For ORR, the onset potential can be calculated by

$U_{\mathrm{ORR}}^{\mathrm{onset}}=-max\{\Delta G_{1},\Delta G_{2},\Delta G_{3},\Delta G_{4}\}$ (18)

The overpotential can be calculated using

$\eta_{\mathrm{ORR}}=\max\left\{ \Delta G_{1},\Delta G_{2},\Delta G_{3},\Delta G_{4} \right\}/e+1.23V$ (19)

**S3 Supplementary Figures and tables**

^
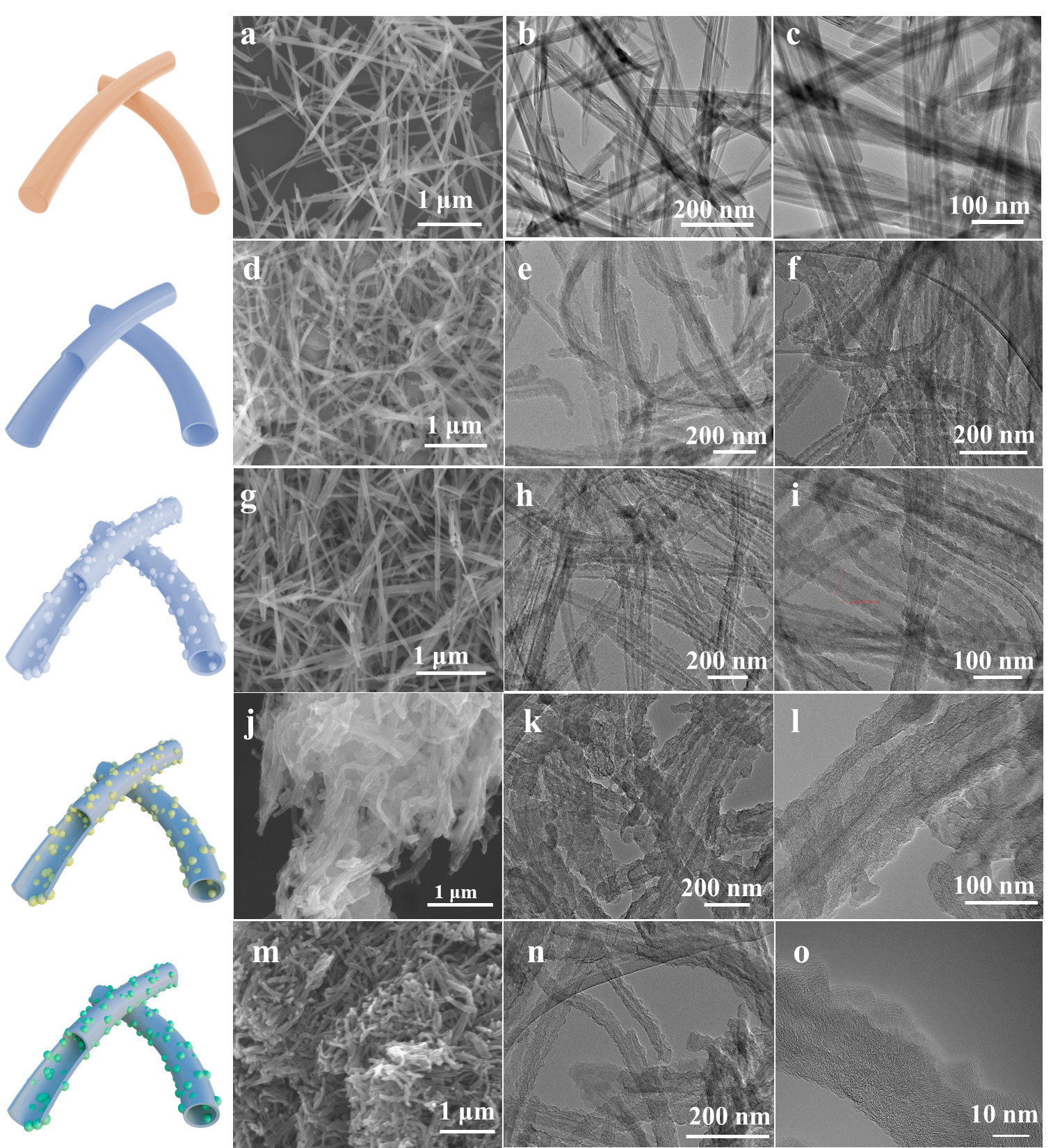
^

Figure S1. SEM and TEM images of MnO_2_ nanowires(a-c), PPy-co-PANI (d-f), PPy-co-PANI-Fe (g-i), Fe-N_4_-NCNT (j-l), FePc-Fe-NCNT (m-o).


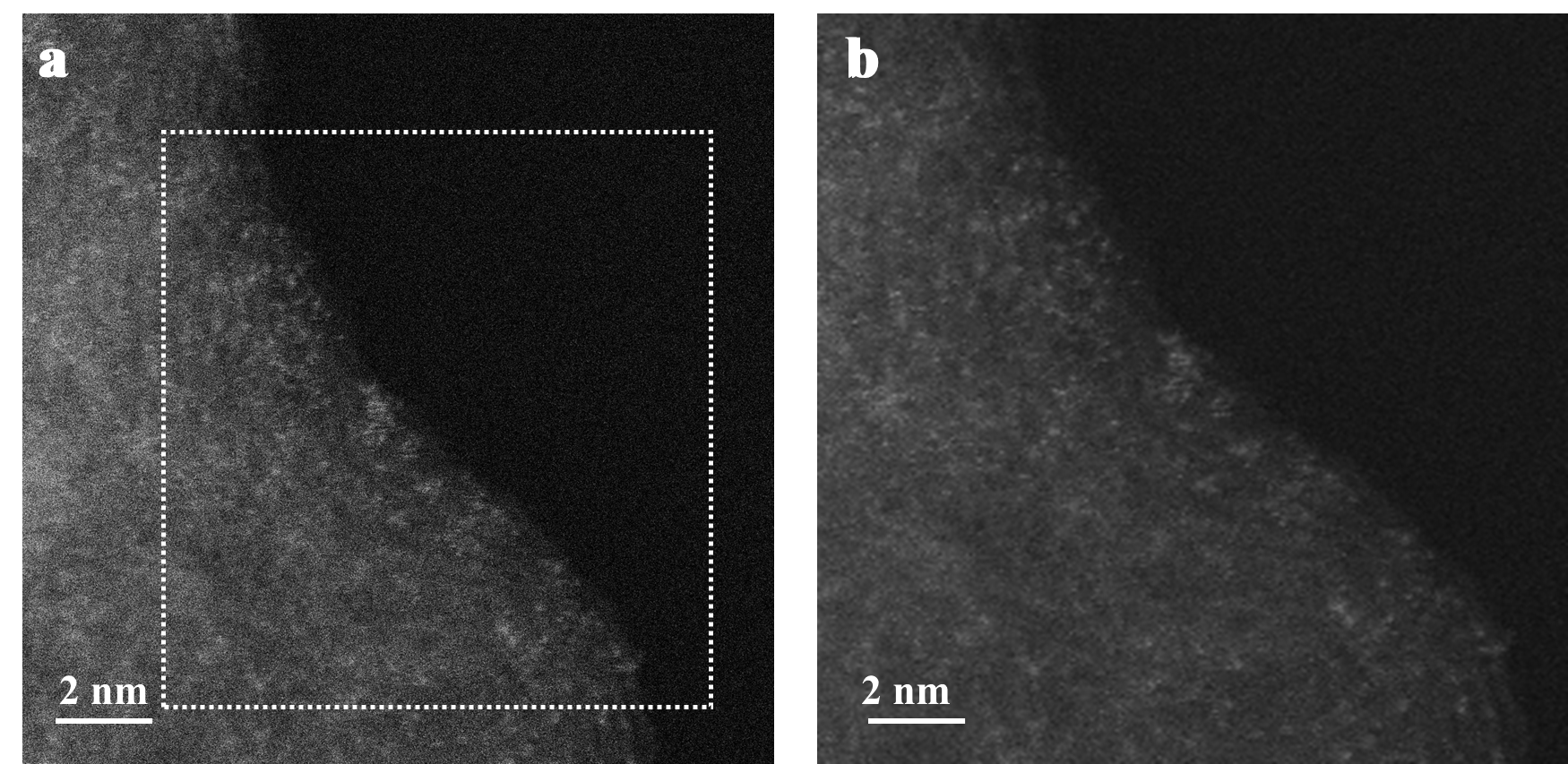


Figure S2. (a) AC HADDF-STEM image and (b) Enlarged intensity image of FePc-Fe-NCNT.


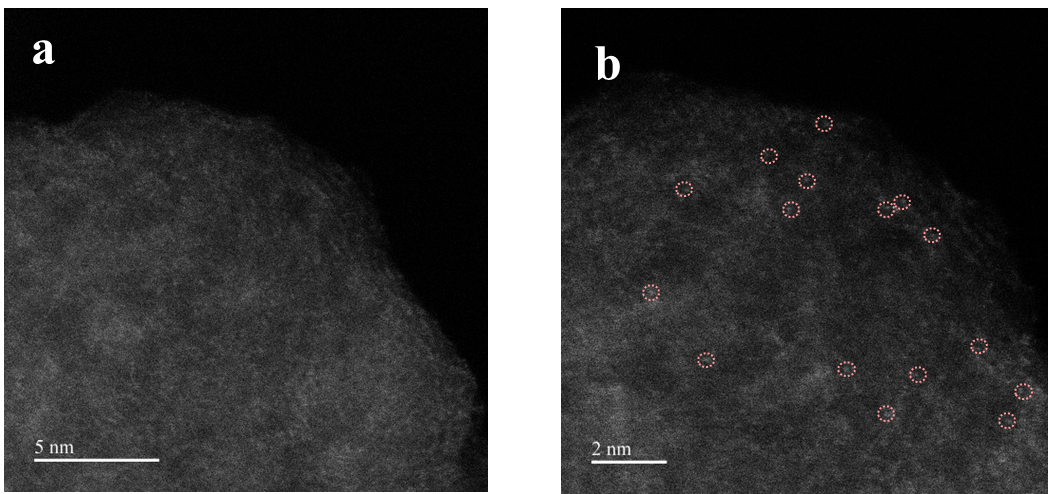


Figure S3. (a, b) HAADF-STEM image of FePc-NCNT.

^
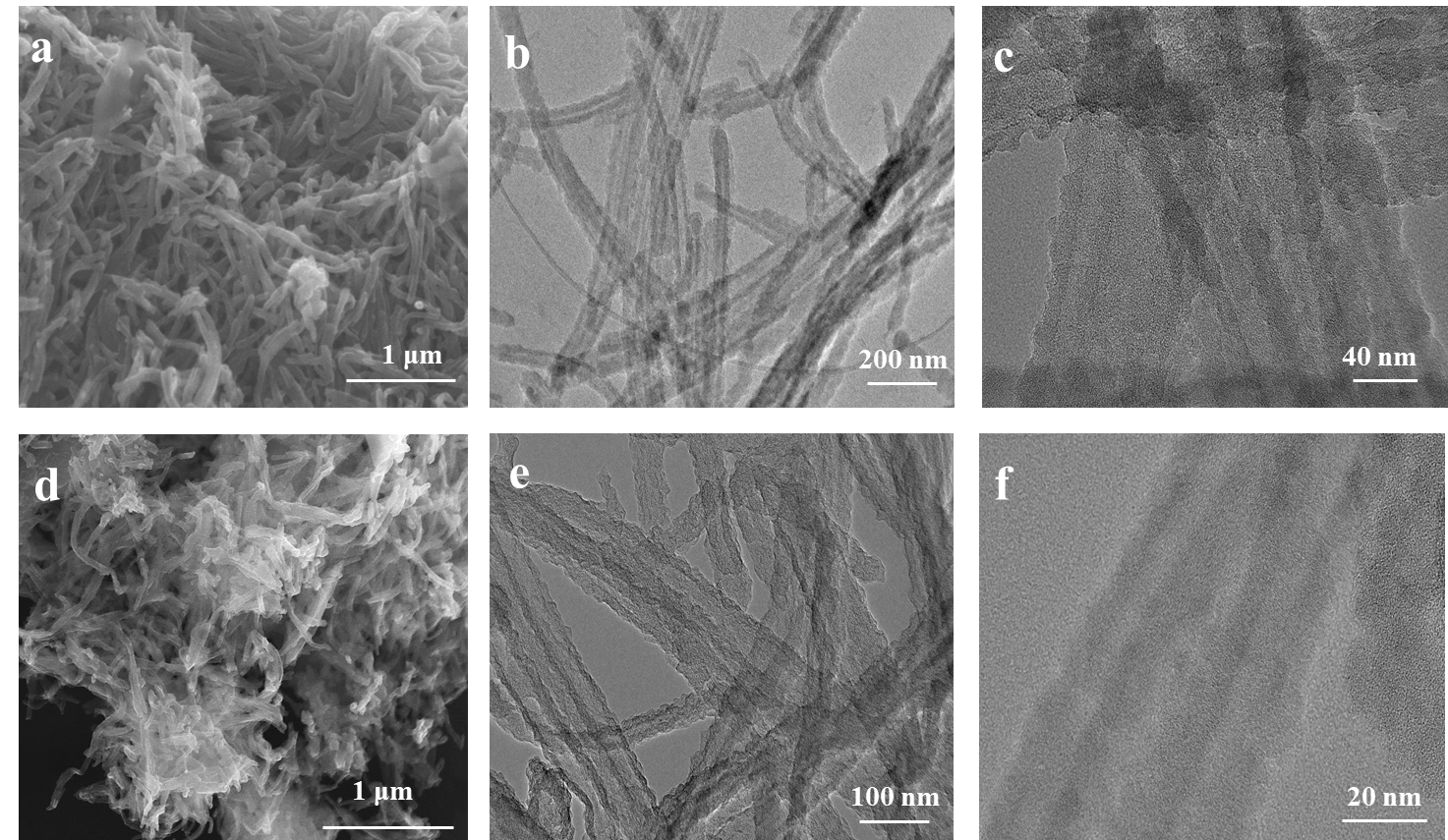
^

Figure S4. SEM and TEM image of FePc-NCNT (a-c), NCNT (d-f).

^
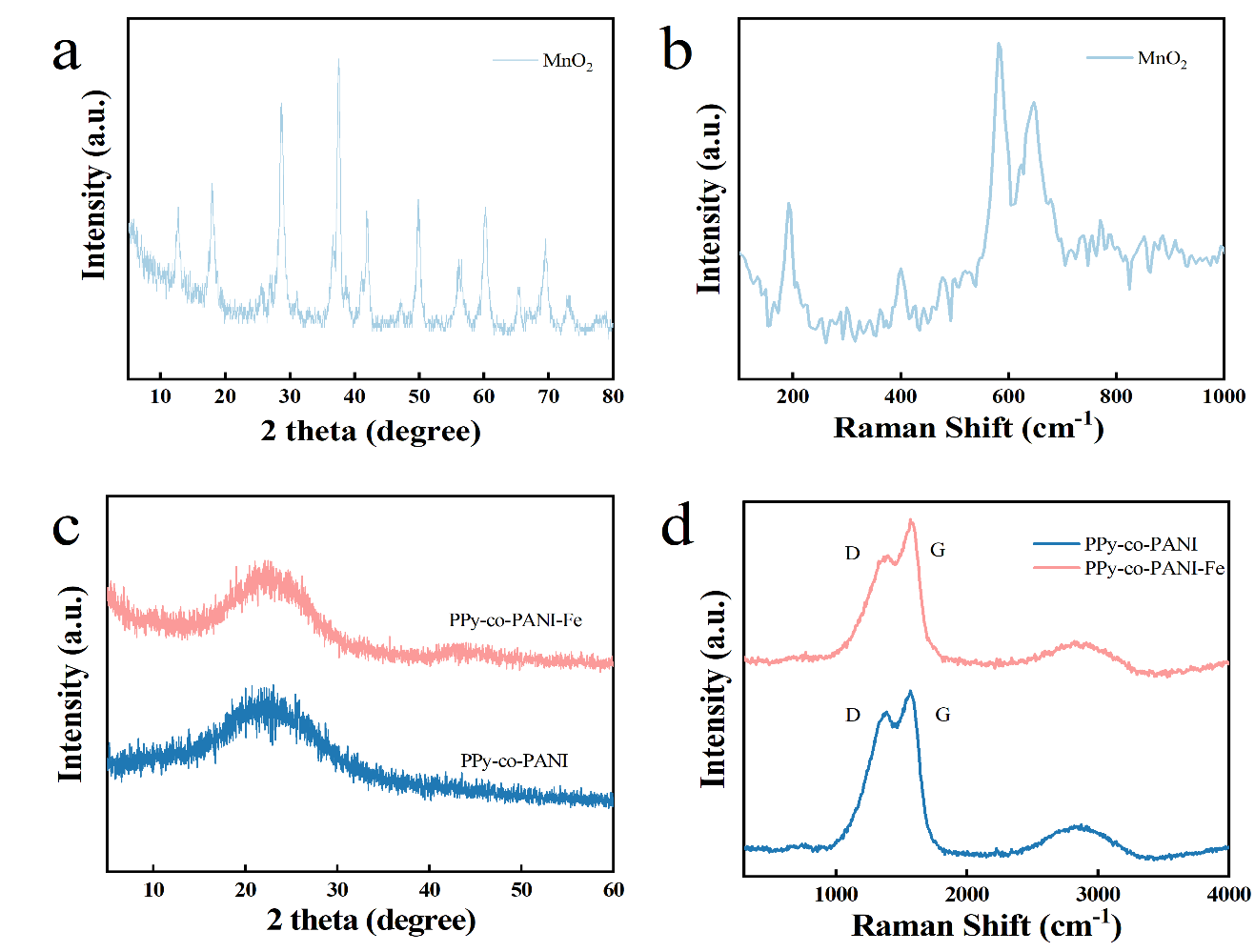
^

Figure S5. XRD patterns of (a) MnO_2_; Raman patterns of (b) MnO_2_; XRD patterns of (c) PPy-co-PANI, and PPy-co-PANI-Fe; Raman patterns of (d) PPy-co-PANI, and PPy-co-PANI-Fe.

^
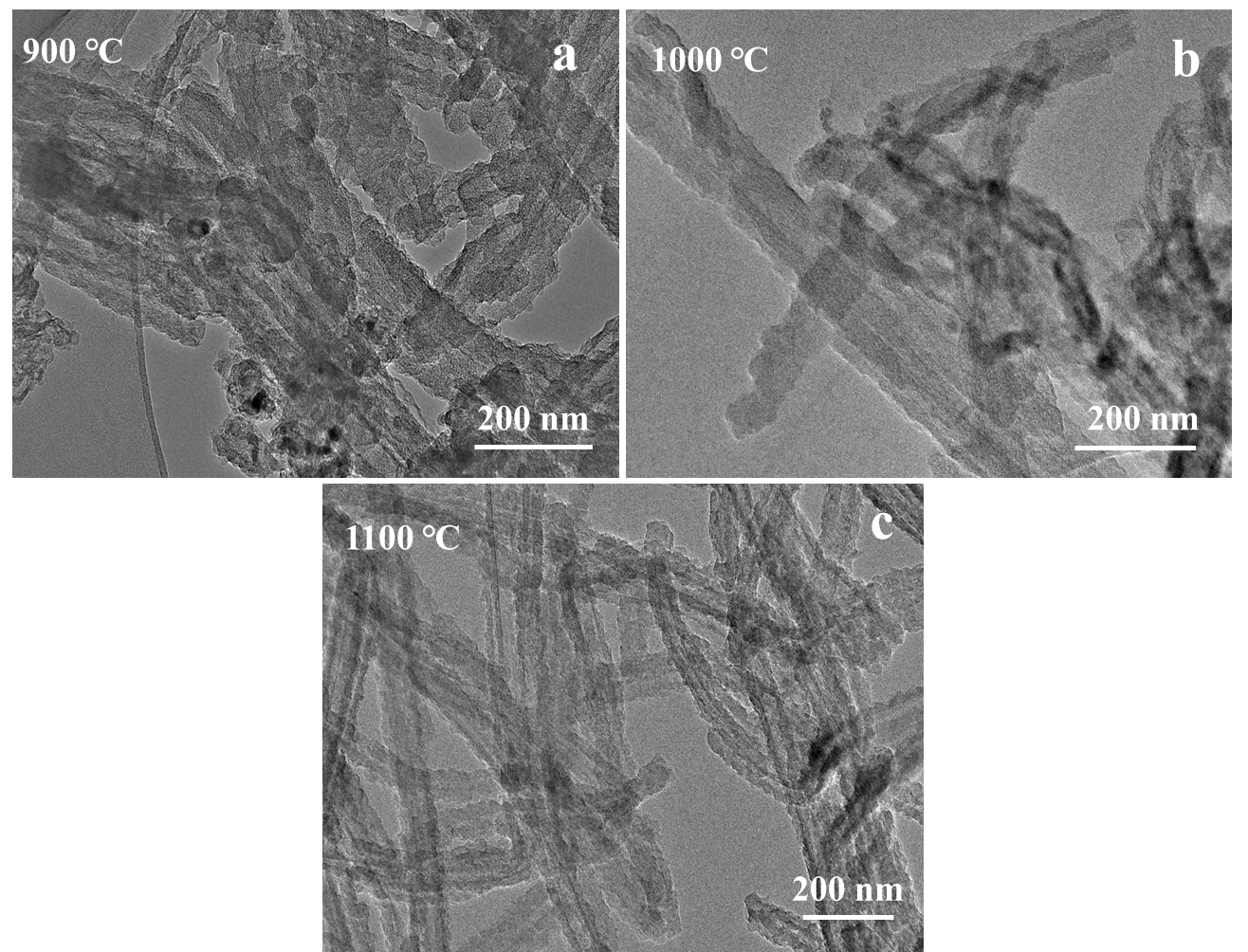
^

Figure S6. TEM image of (a) Fe-N_4_-NCNT-900 ℃, (b) Fe-N_4_-NCNT-1000 ℃, (c) Fe-N_4_-1100 ℃.

^
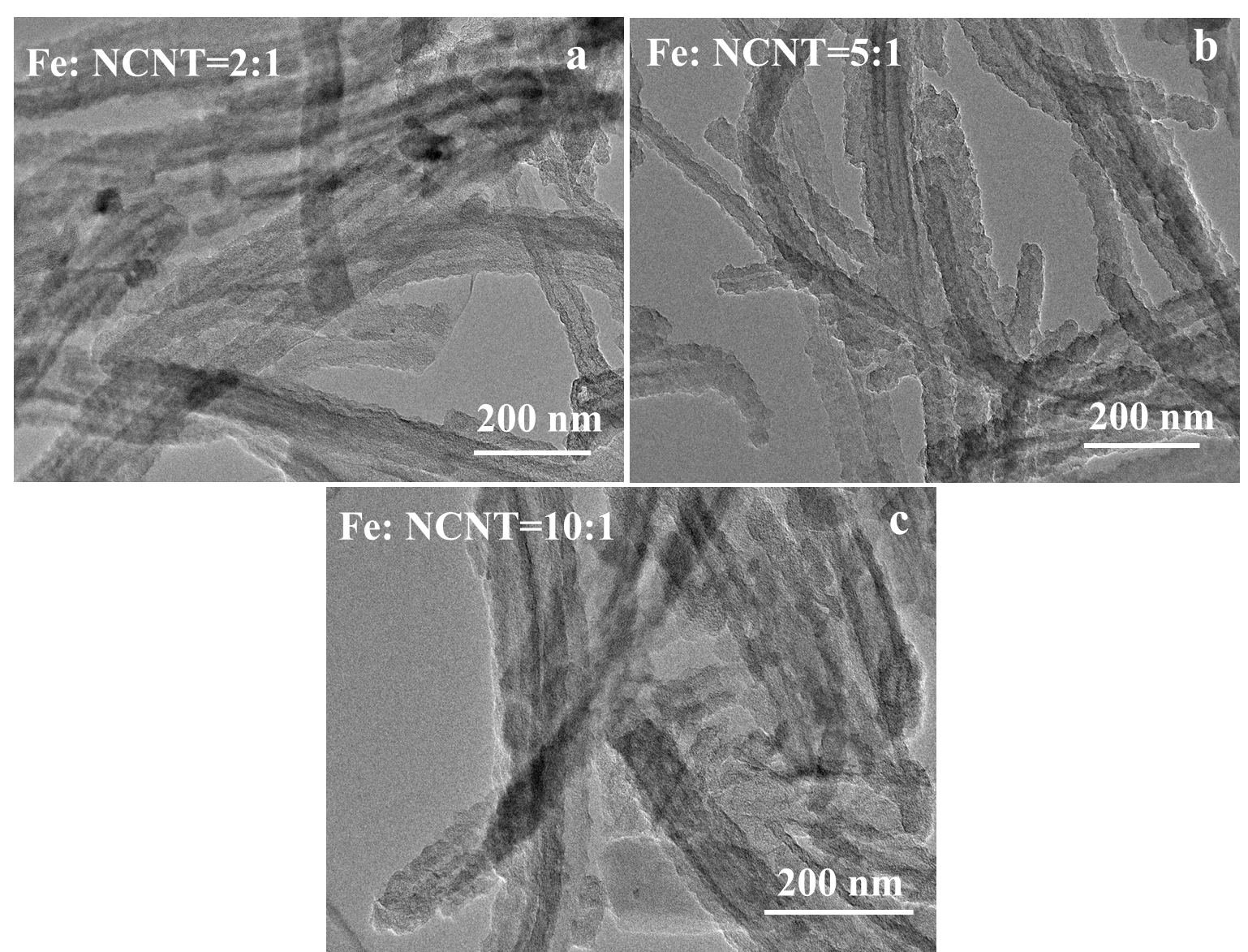
^

Figure S7. TEM image of Fe: NCNT (a) 2:1, (b) 5:1, (c) 10:1.

^
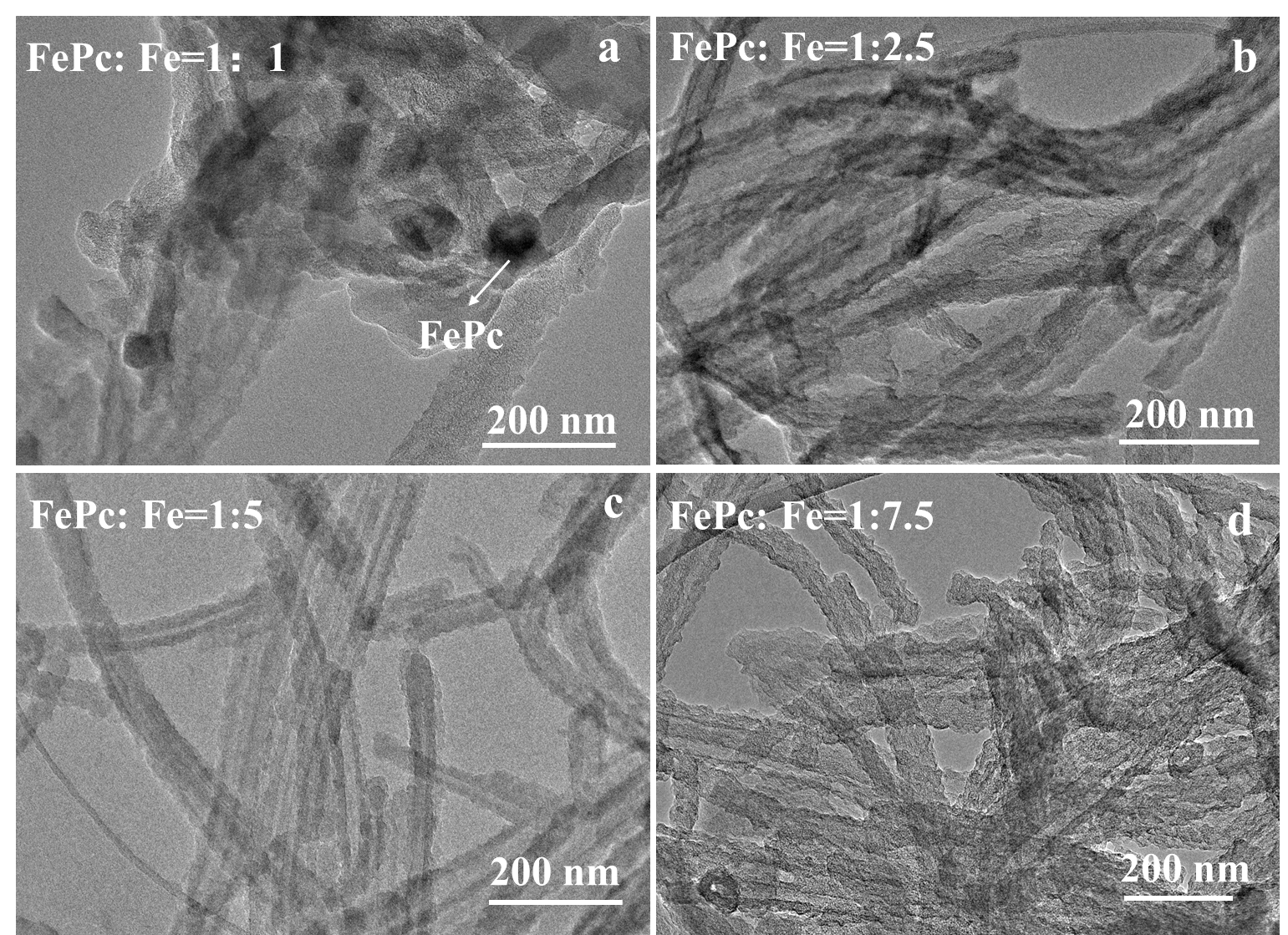
^

Figure S8. TEM image of FePc: Fe (a)1:1, (b) 1:2.5, (c) 1:5, (d) 1:7.5.

^
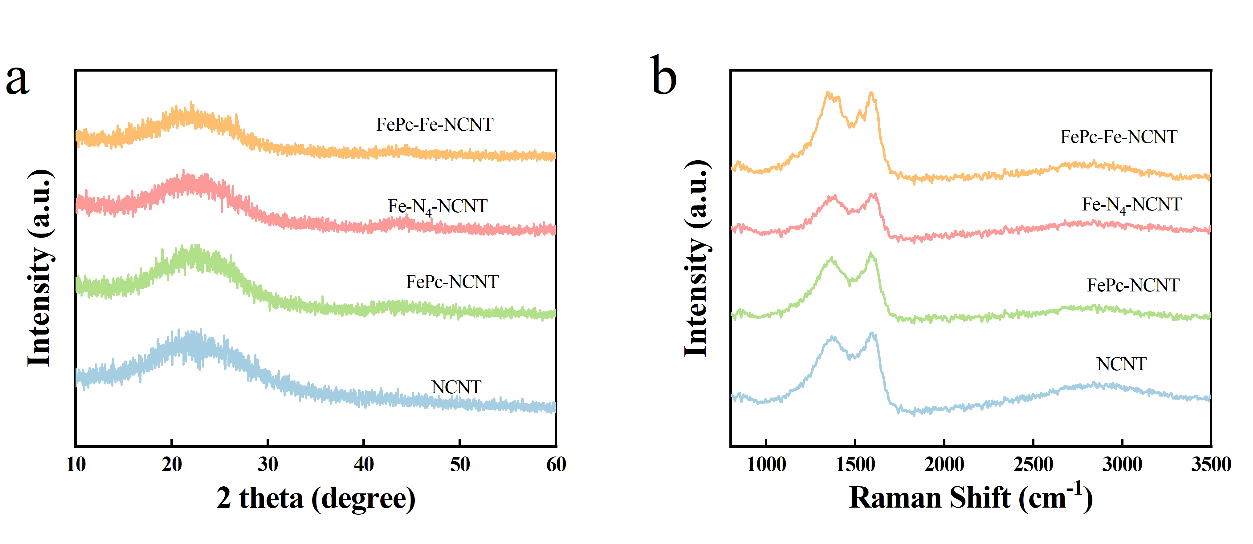
^

Figure S9. XRD patterns of (a) FePc-Fe-NCNT, Fe-N_4_-NCNT, FePc-NCNT and NCNT; (b) Raman of FePc-Fe-NCNT, Fe-N_4_-NCNT, FePc-NCNT and NCNT.


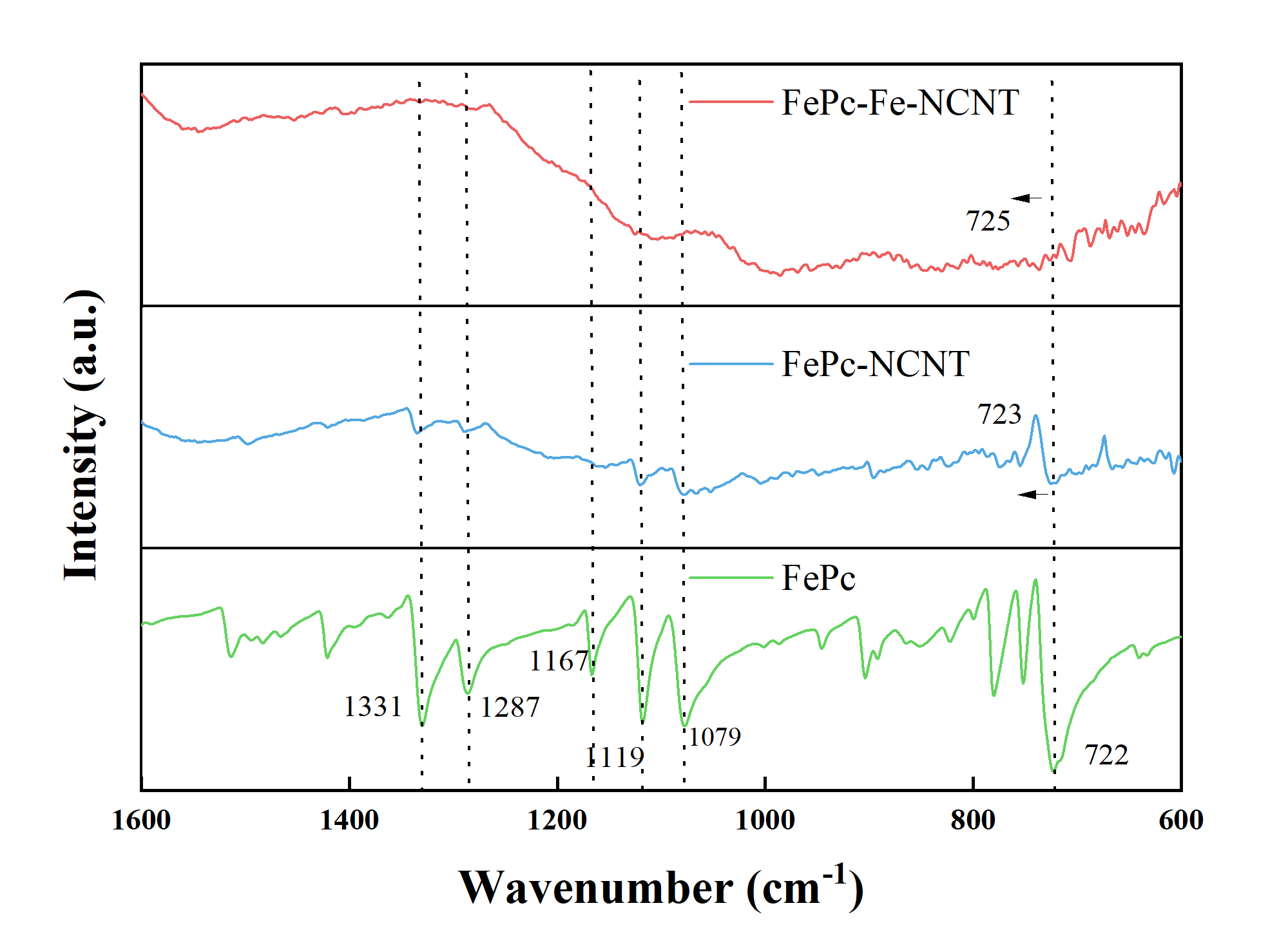


Figure S10. FT-IR spectra of FePc-Fe-NCNT, FePc-NCNT, and FePc.


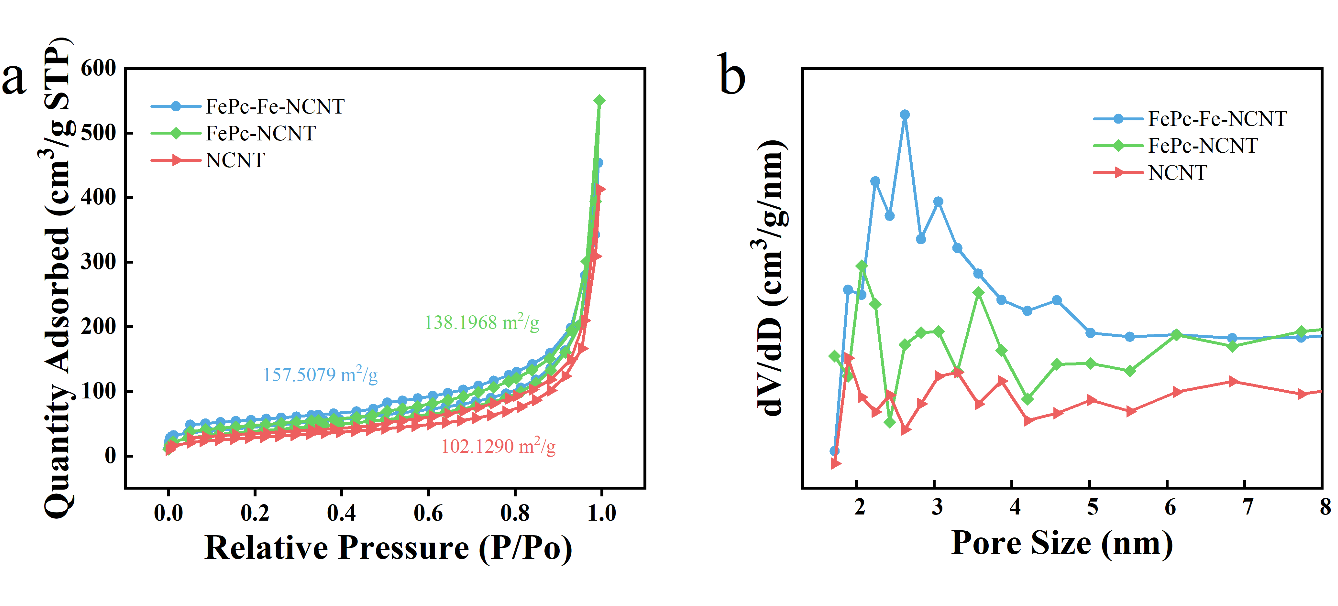


Figure S11. (a) N_2_ adsorption-desorption isotherms and (b) pore size distribution of FePc-Fe-NCNT FePc-NCNT and NCNT.

^
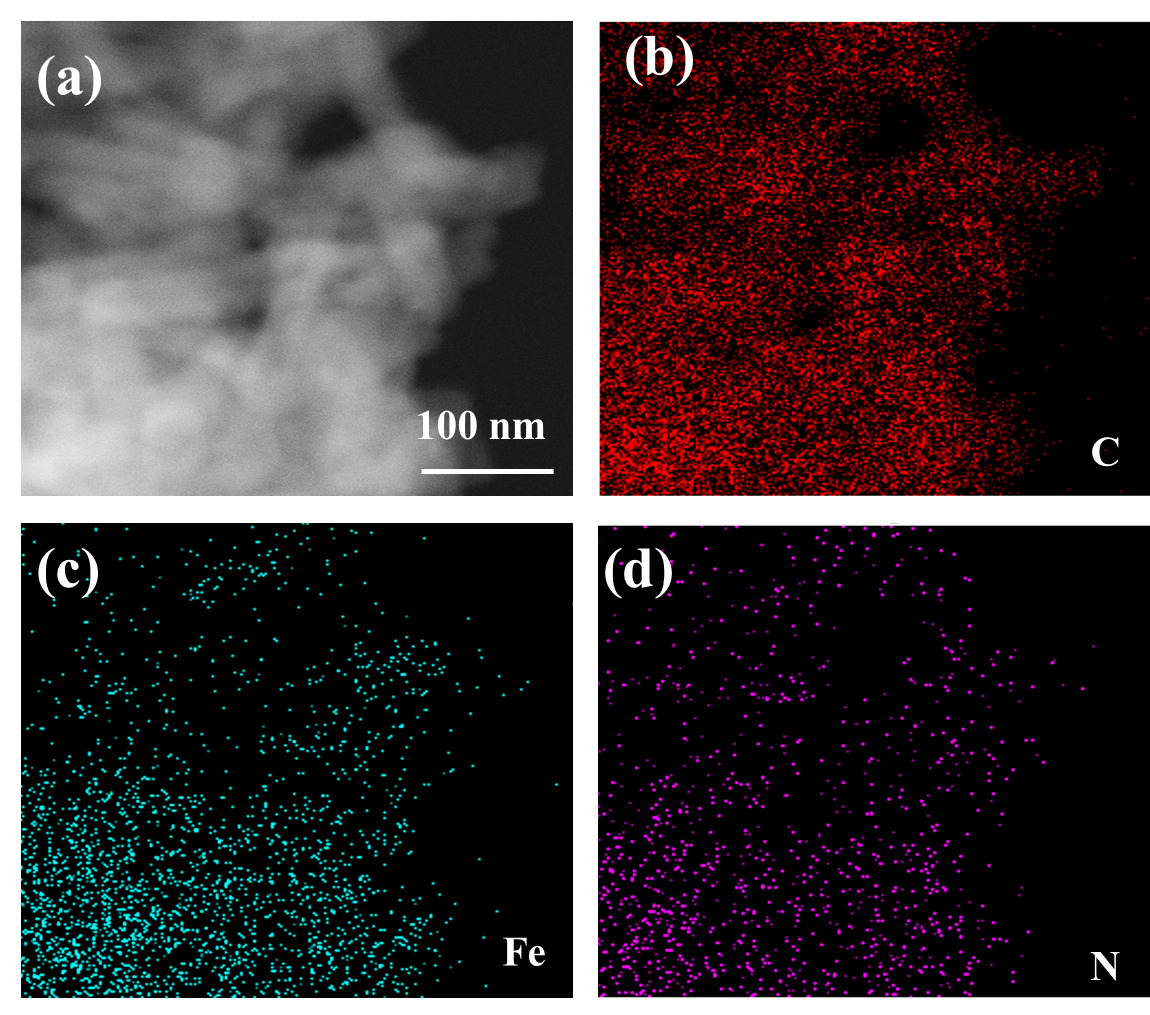
^

Figure S12. (a) STEM images and (b-d) corresponding EDS element mapping of FePc-NCNT.


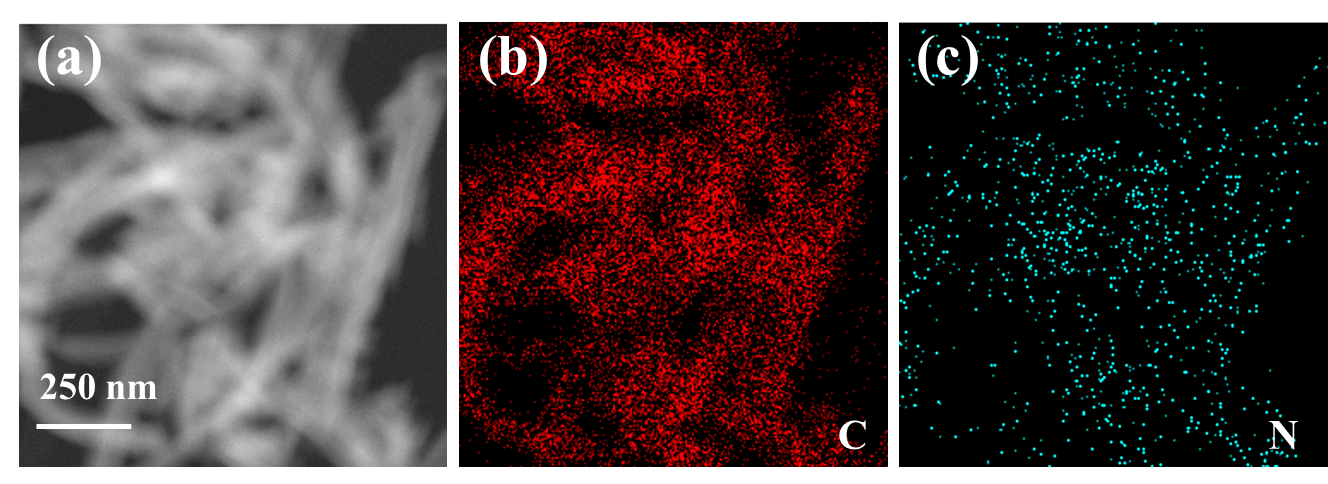


Figure S13. (a) STEM images and (b, c) corresponding EDS element mapping of NCNT.


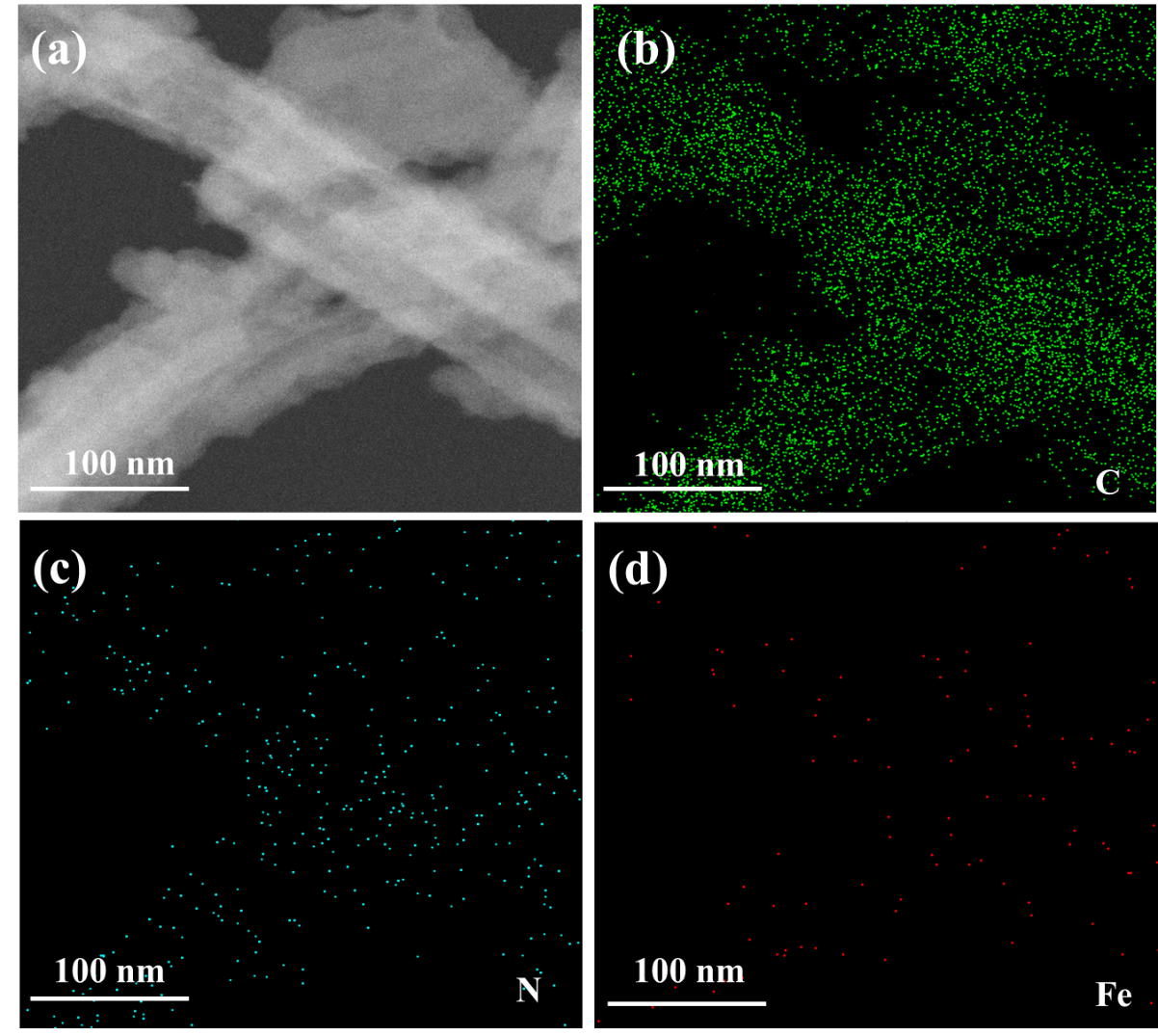


Figure S14. (a) STEM images and (b-d) corresponding EDS element mapping of Fe-N_4_-NCNT.

^
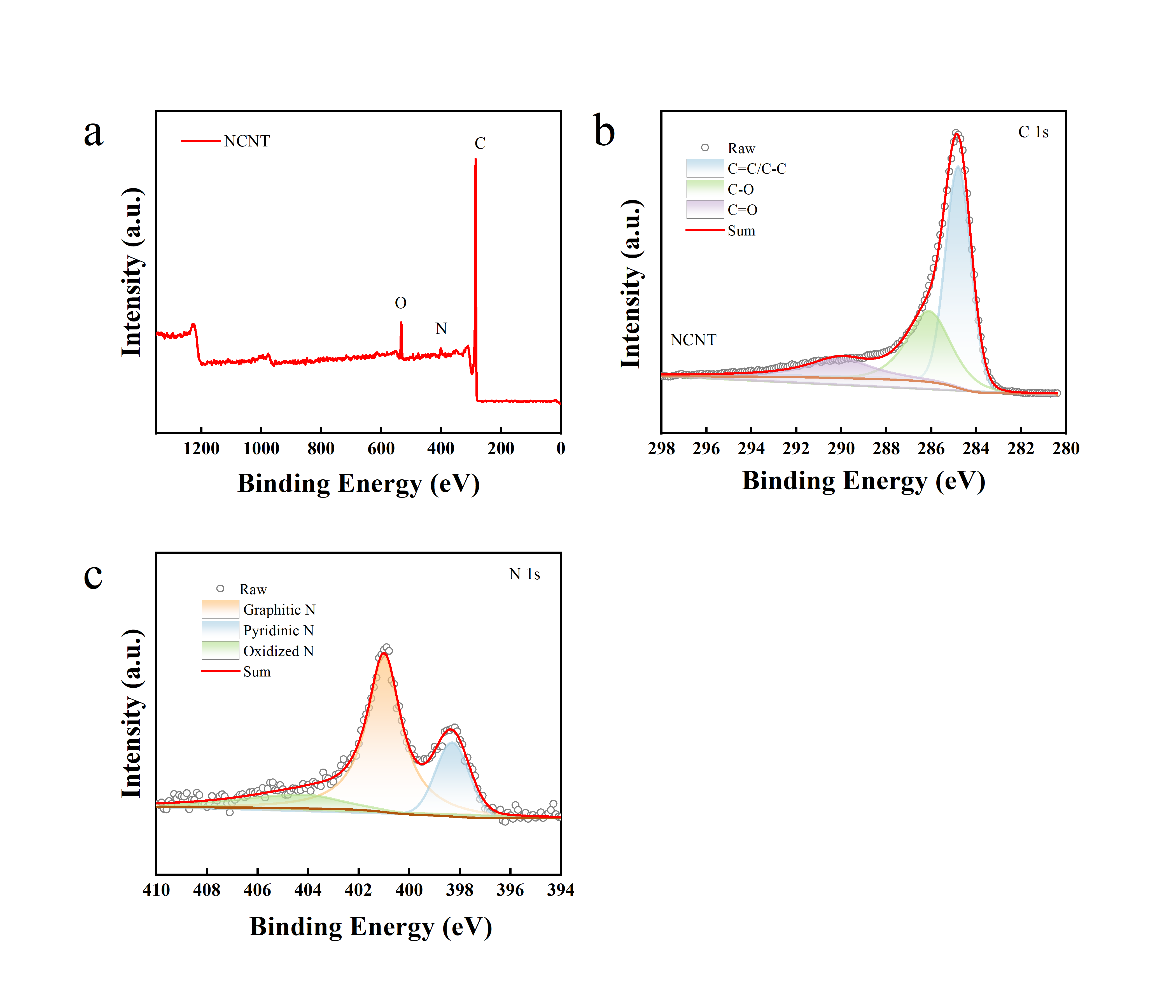
^

Figure S15. The XPS spectra for all (a), C 1s (b), N 1s (c) of NCNT.

^
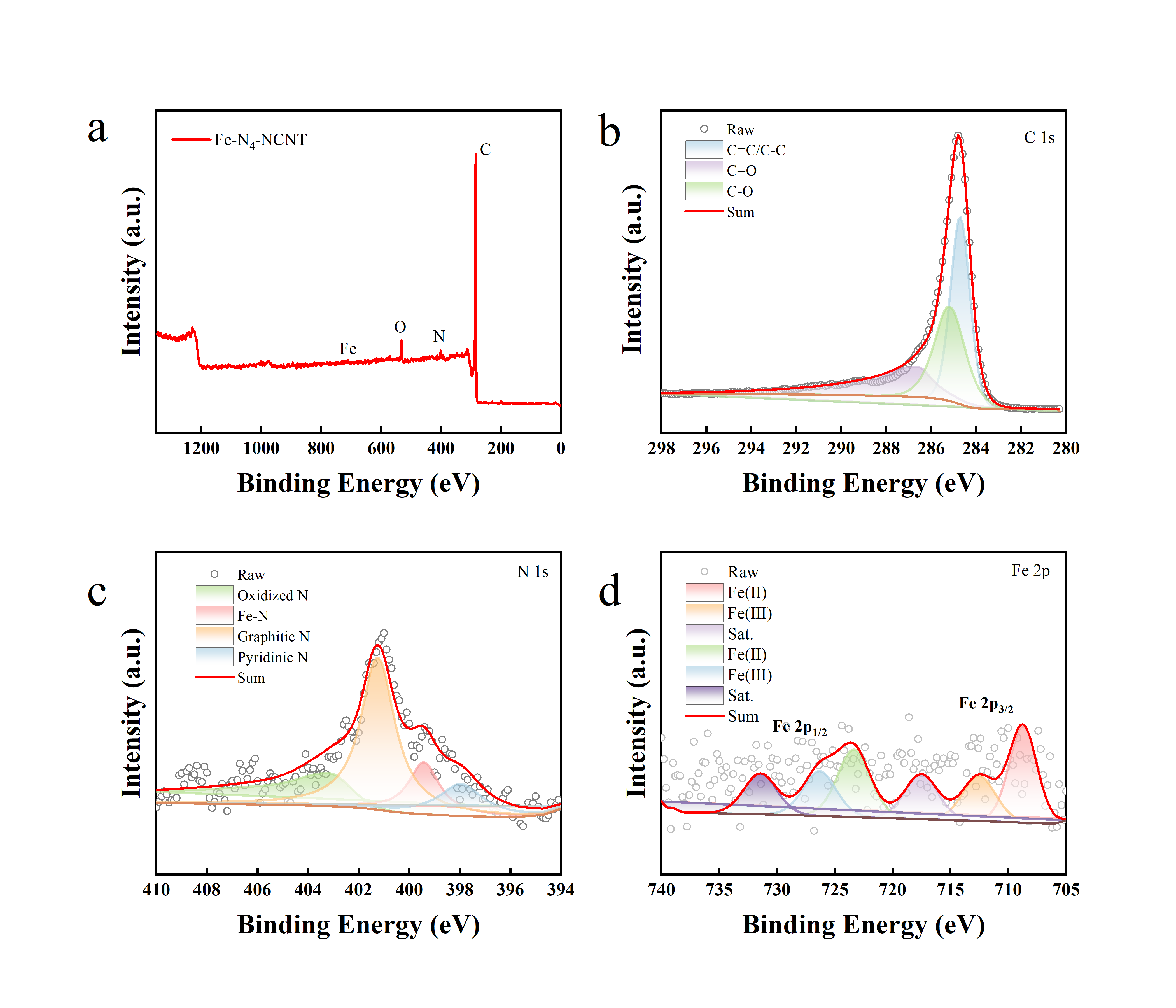
^

Figure S16. The XPS spectra for all (a), C 1s (b), N 1s (c), Fe 2p (d) of Fe-N_4_-NCNT.


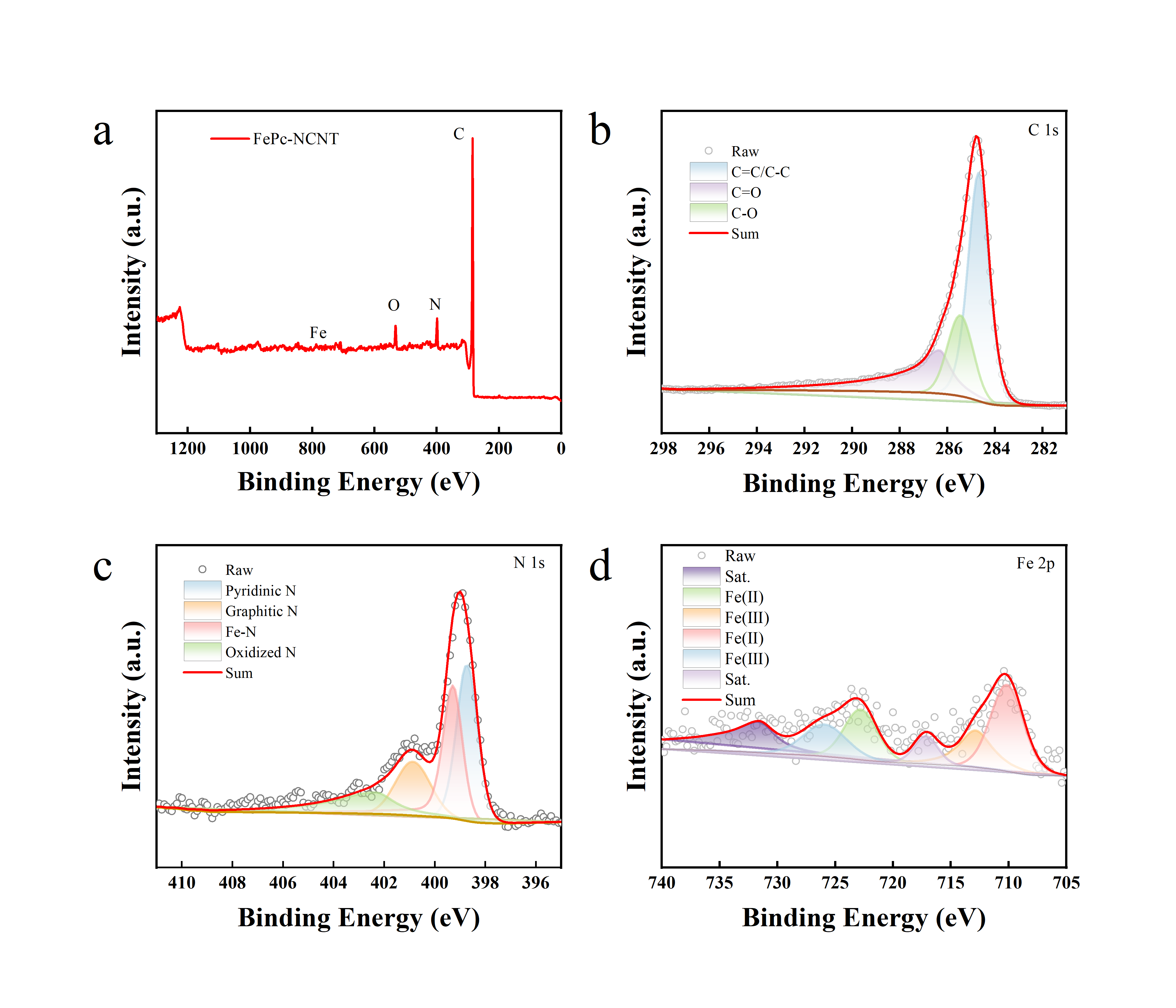


Figure S17. The XPS spectra for all (a), C 1s (b), N 1s (c), Fe 2p (d) of FePc-NCNT.


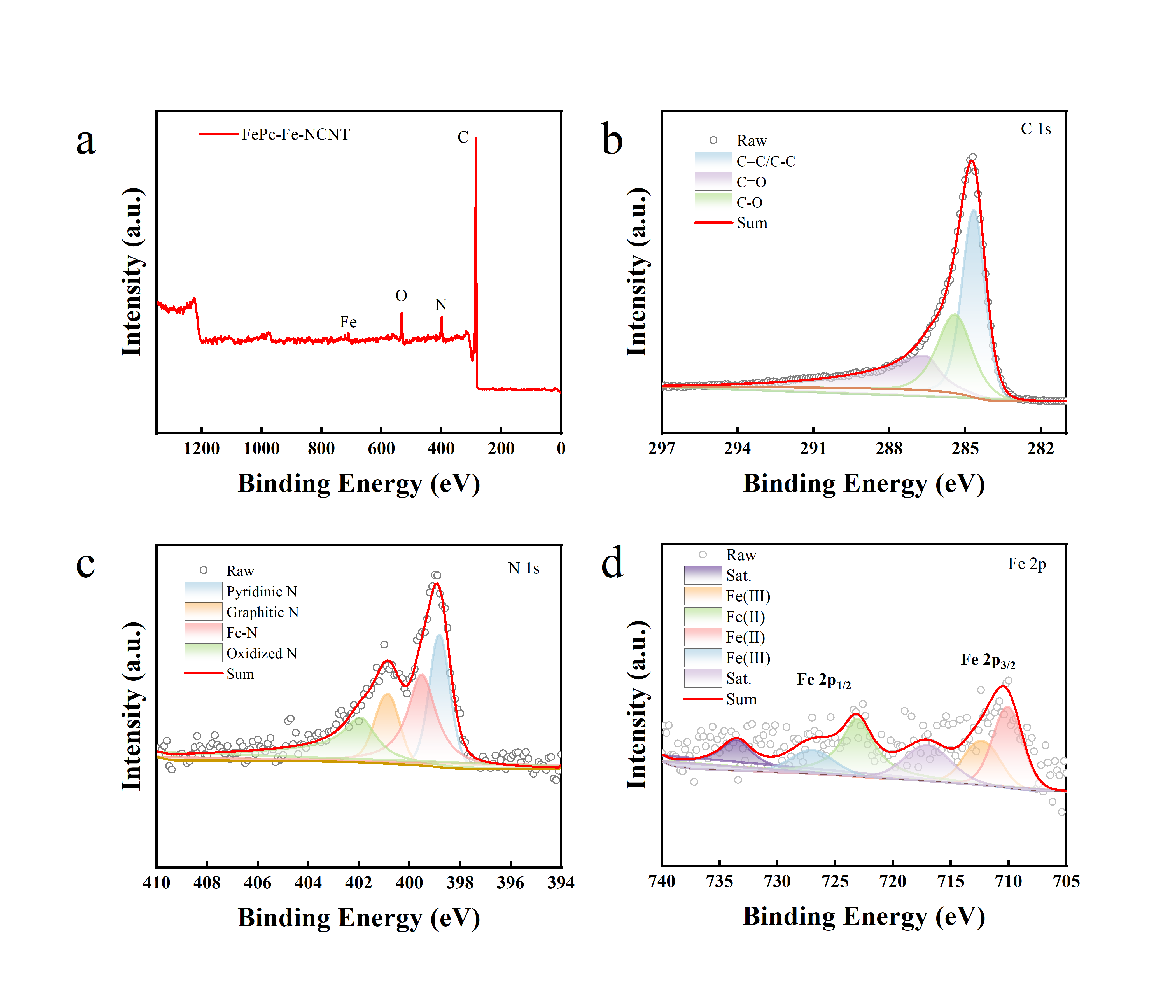


Figure S18. The XPS spectra for all (a), C 1s (b), N 1s (c), Fe 2p (d) of FePc-Fe-NCNT.


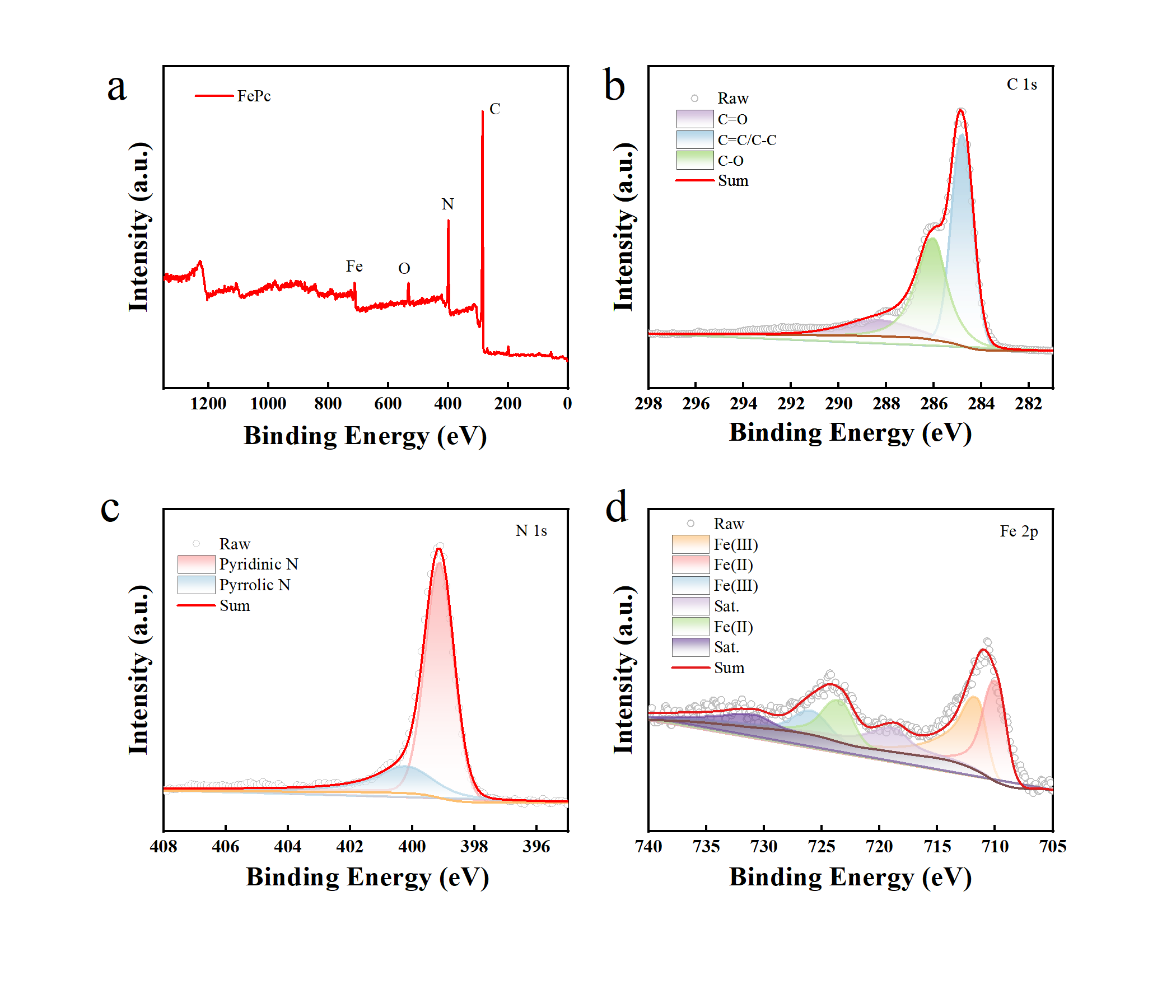


Figure S19. The XPS spectra for all (a), C 1s (b), N 1s (c), Fe 2p (d) of FePc.


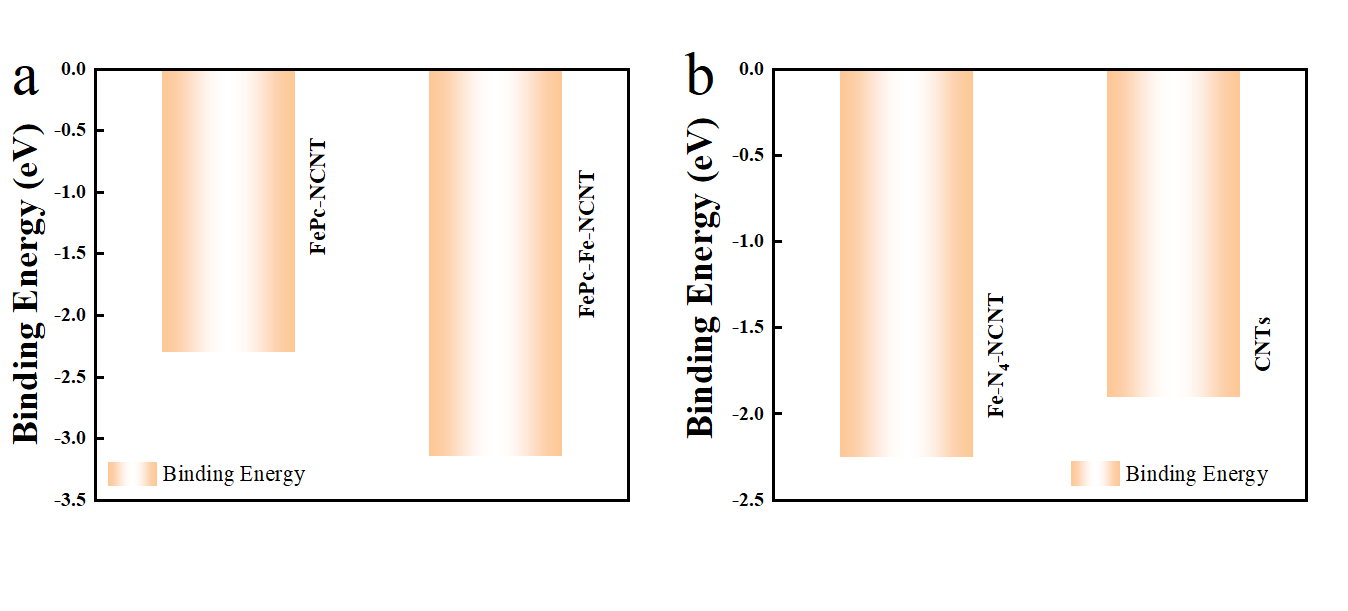


Figure S20. (a)The binding energy of FePc-NCNT and FePc-Fe-NCNT; (b) The binding energy of FePc adsorb onto Fe-N_4_ sites and CNTs.

Density functional theory (DFT) calculations show that FePc exhibits a more favorable interaction with Fe-N_4_ sites (−2.250 eV) than with the CNT surface (−1.902 eV), indicating preferential adsorption on the Fe–N_4_ coordination center.


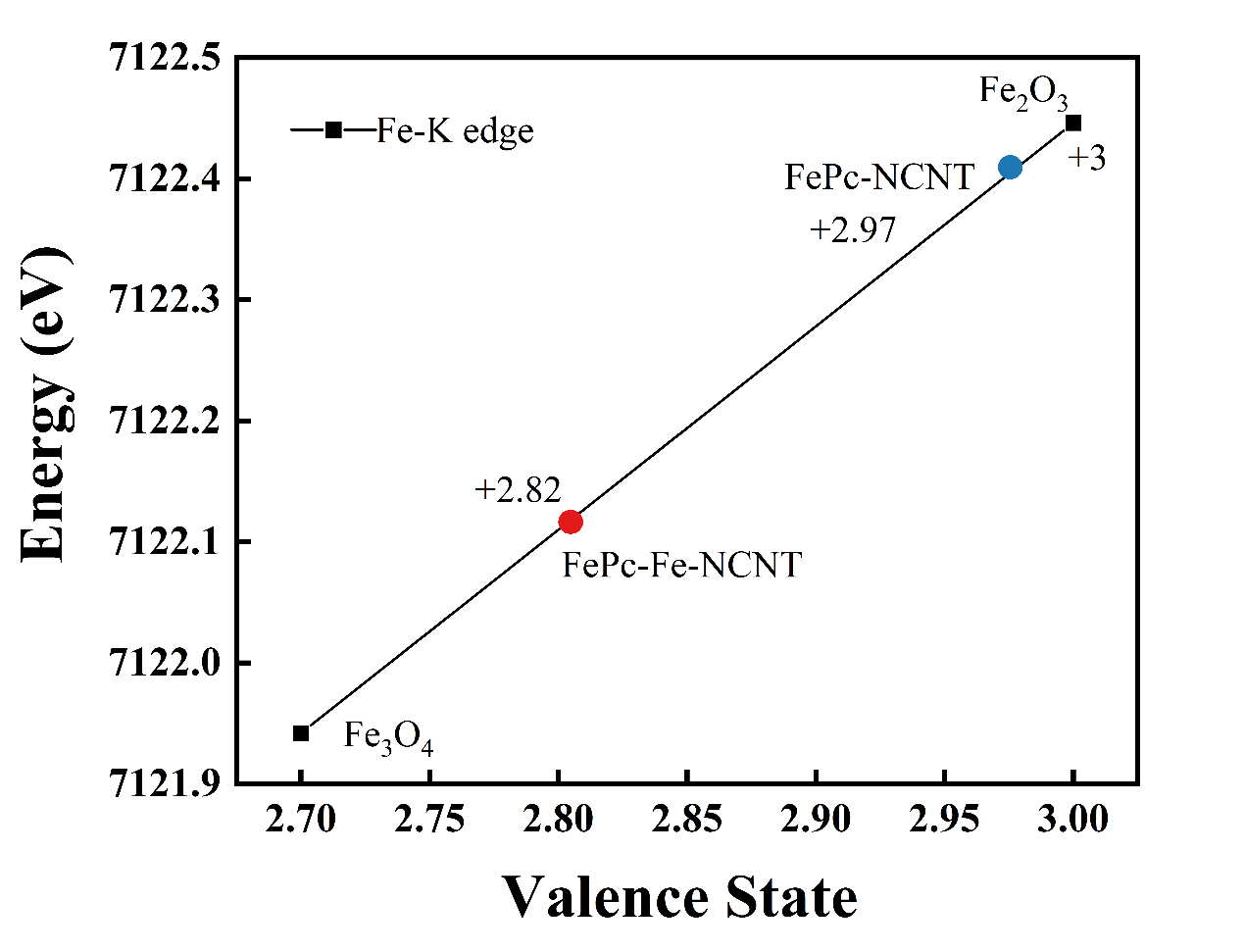


Figure S21. The average oxidation states of Fe from XANES.


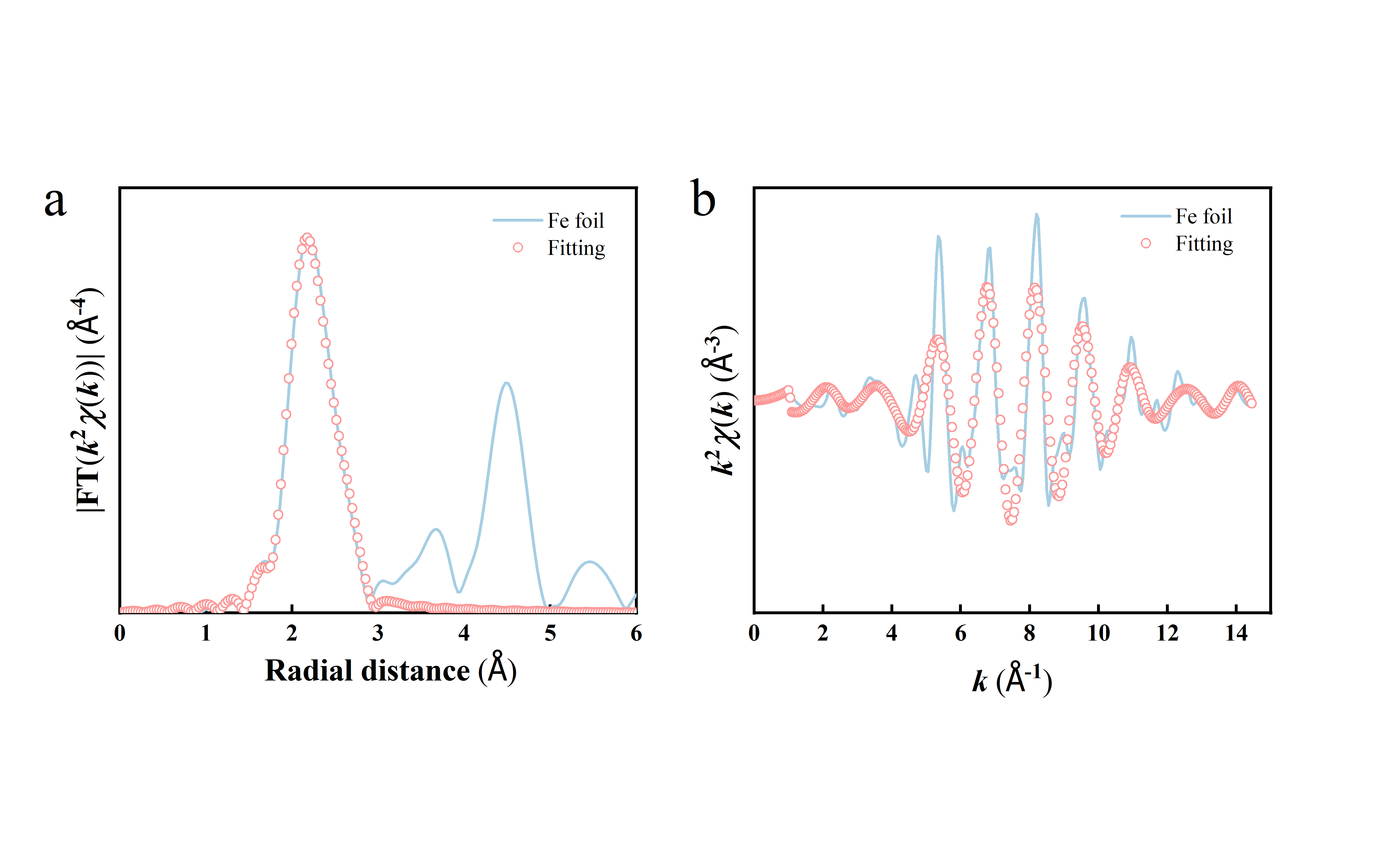


Figure S22. The Fourier-transformed experimental EXAFS spectrum and fitted spectrum of Fe foil: (a) EXAFS R space, (b) EXAFS k space.


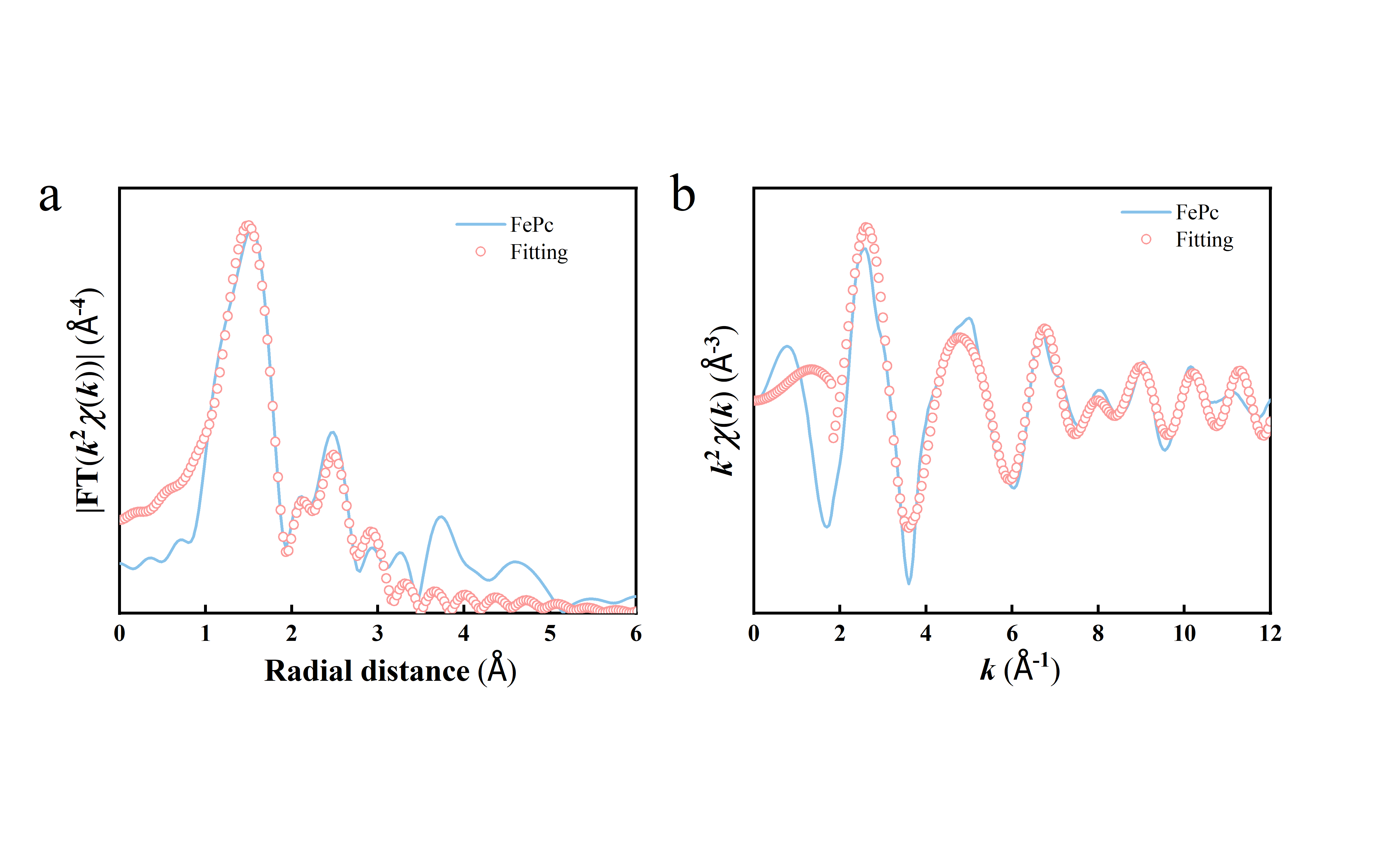


Figure S23. The Fourier-transformed experimental EXAFS spectrum and fitted spectrum of FePc: (a) EXAFS R space, (b) EXAFS k space.


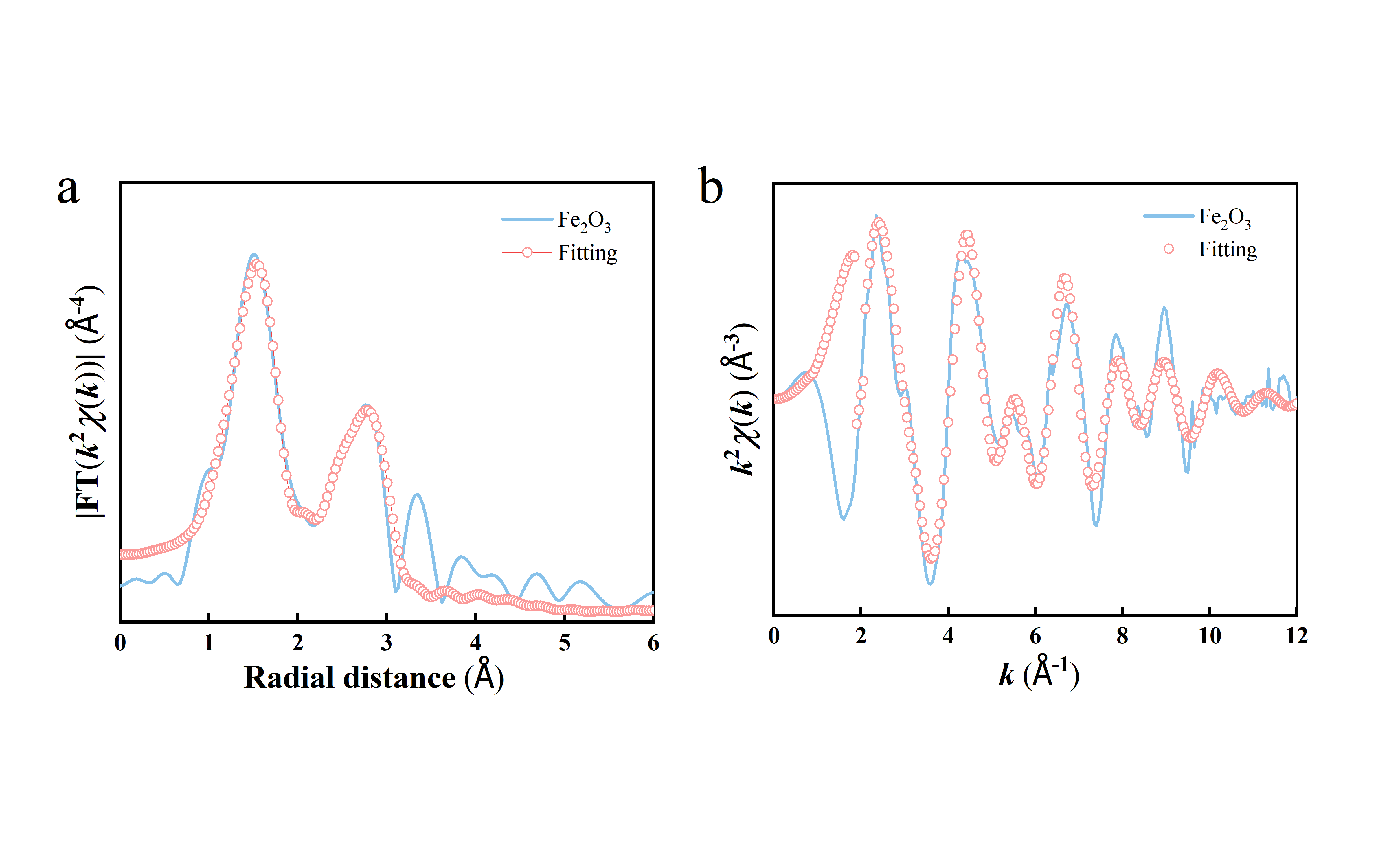


Figure S24. The Fourier-transformed experimental EXAFS spectrum and fitted spectrum of Fe_2_O_3_: (a) EXAFS R space, (b) EXAFS k space.


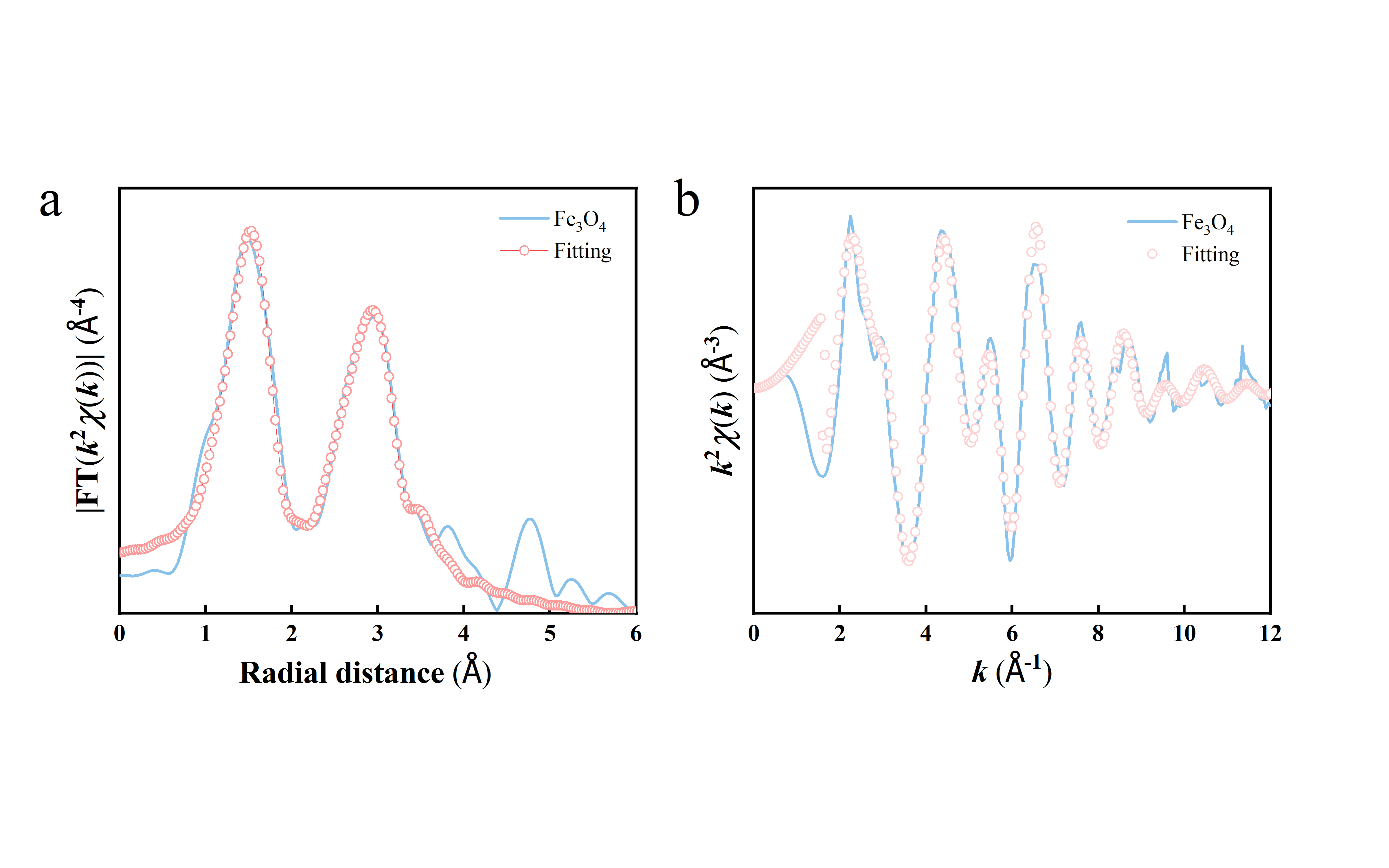


Figure S25. The Fourier-transformed experimental EXAFS spectrum and fitted spectrum of Fe_3_O_4_: (a) EXAFS R space, (b) EXAFS k space.


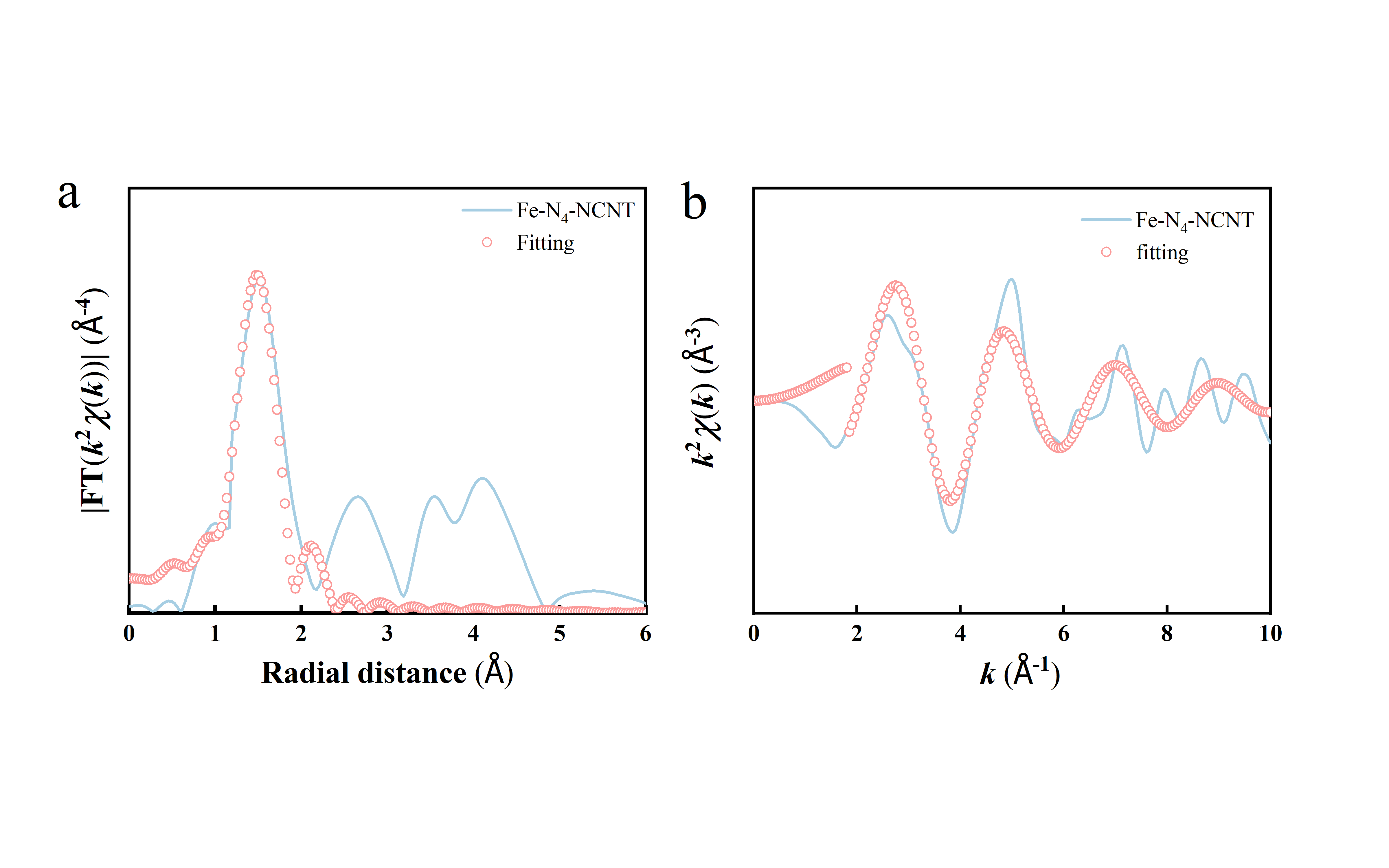


Figure S26. The Fourier-transformed experimental EXAFS spectrum and fitted spectrum of Fe-N_4_-NCNT: (a) EXAFS R space, (b) EXAFS k space.


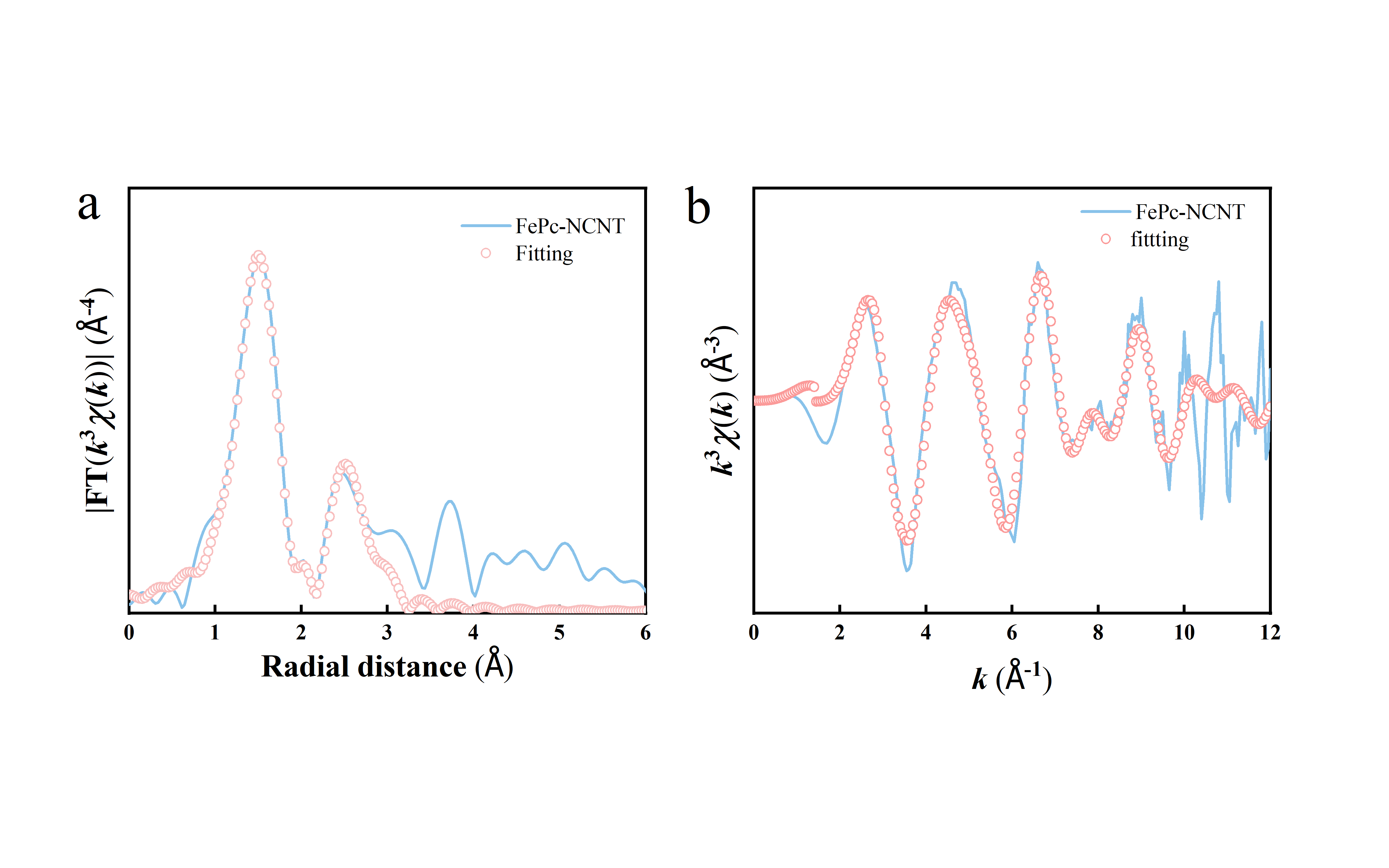


Figure S27. The Fourier-transformed experimental EXAFS spectrum and fitted spectrum of FePc-NCNT: (a) EXAFS R space, (b) EXAFS k space.


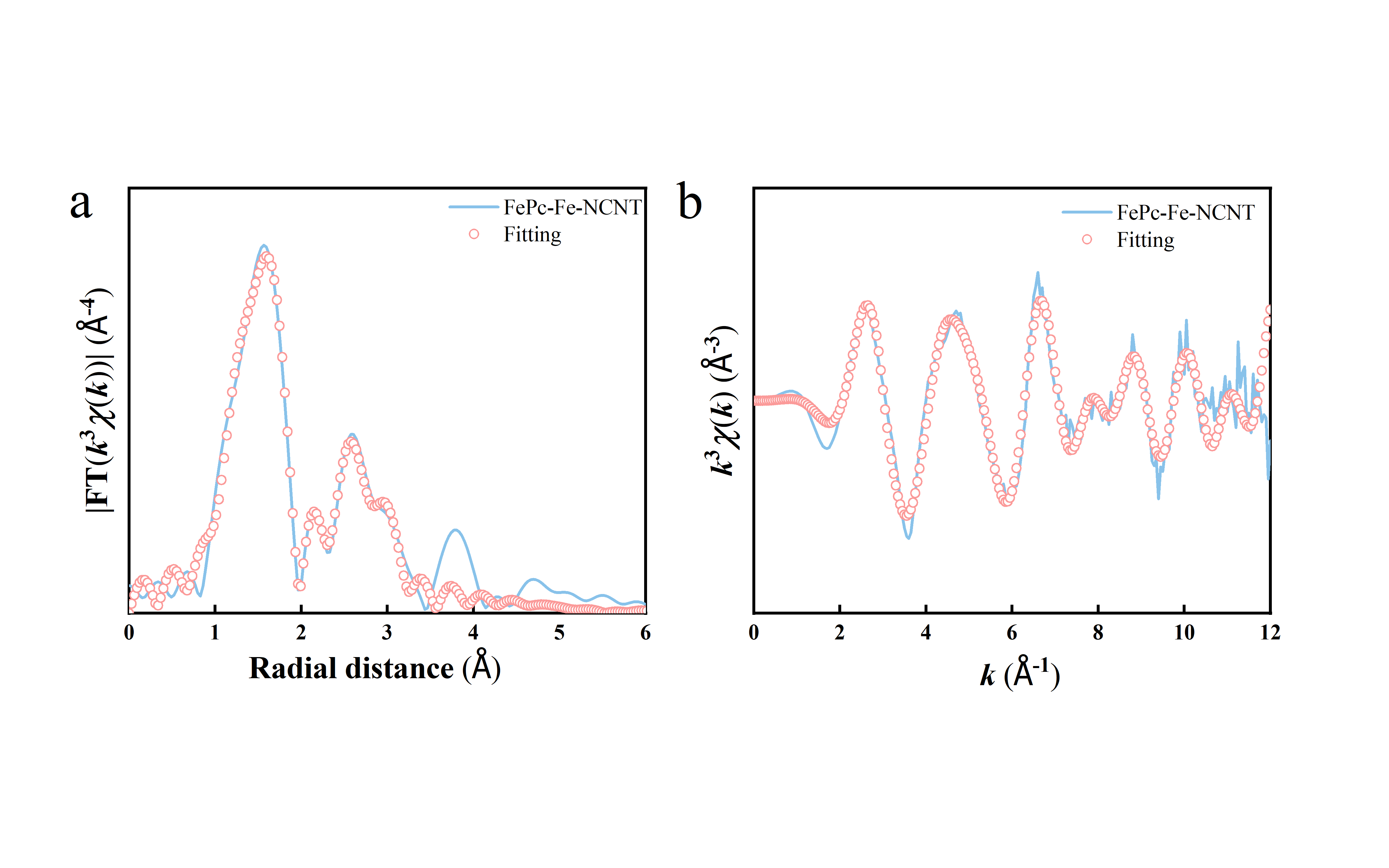


Figure S28. The Fourier-transformed experimental EXAFS spectrum and fitted spectrum of FePc-Fe-NCNT: (a) EXAFS R space, (b) EXAFS k space.


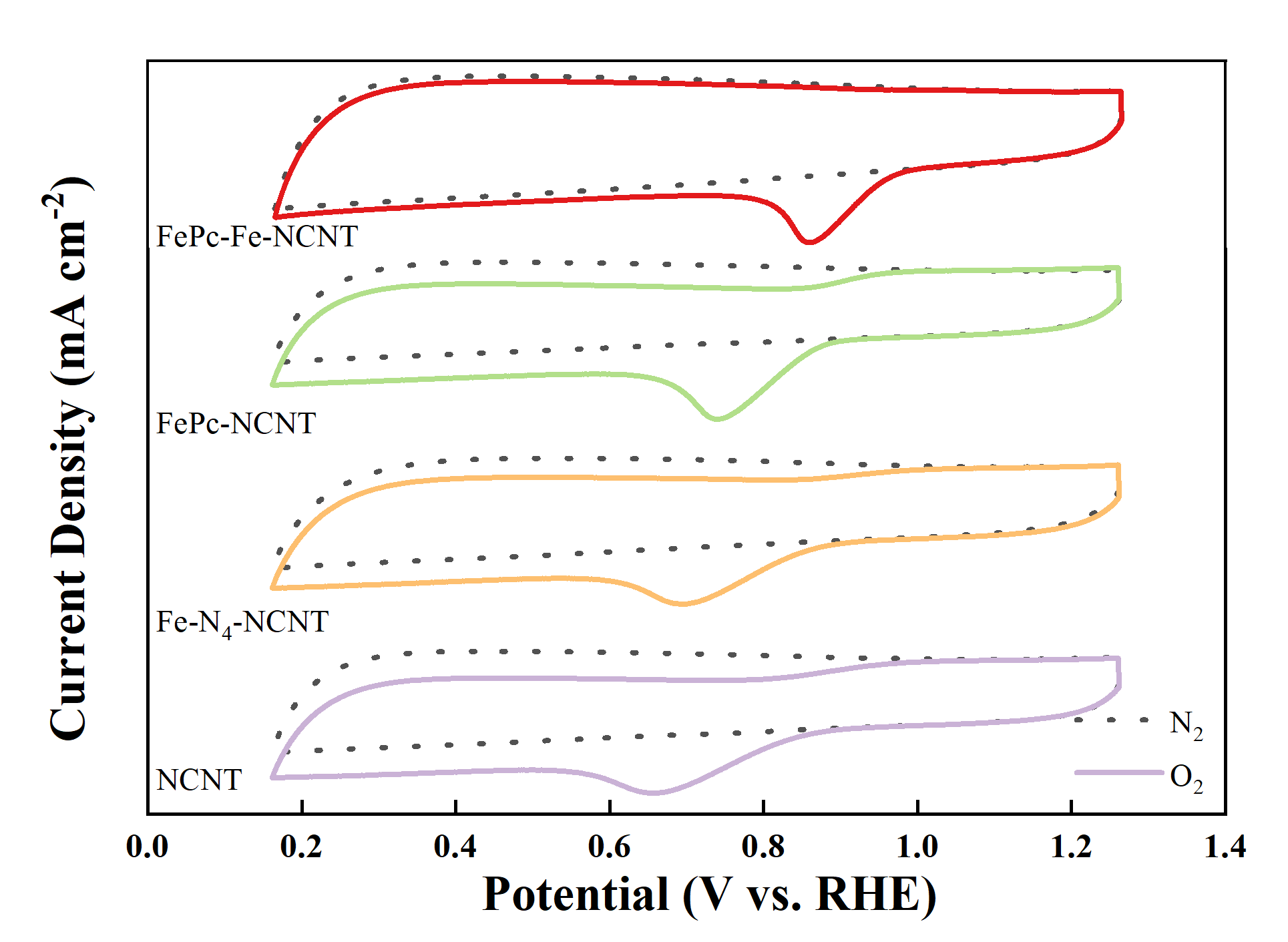


Figure S29. CV curves of FePc-Fe-NCNT and other catalysts in 0.1M KOH.


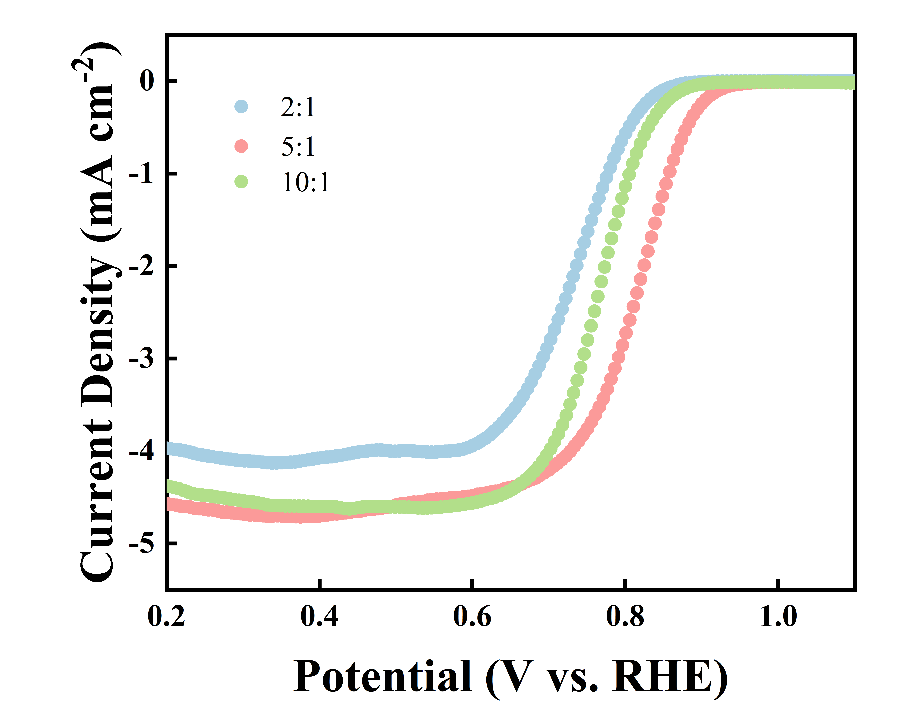


Figure S30. LSV polarization curves of Fe-N_4_-NCNT with different Fe loadings (the mass ratios of FeCl_3_ 6H_2_O to PPy-co-PANI are 2:1, 5:1, and 10:1, respectively).


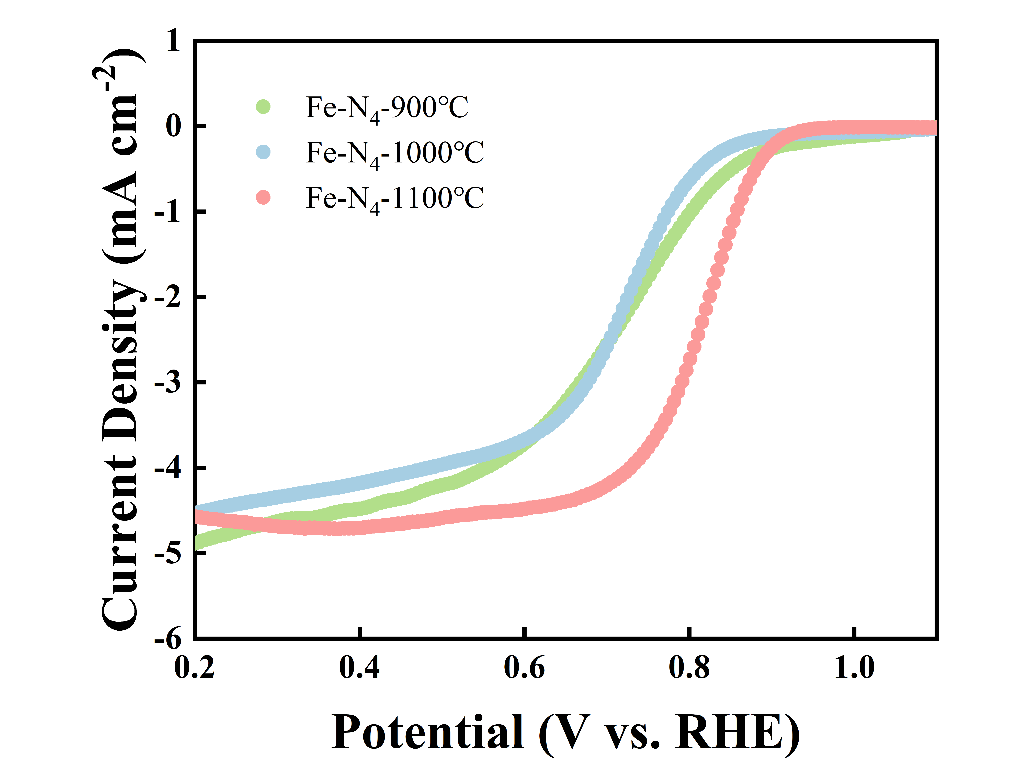


Figure S31. LSV polarization curves of Fe-Nx-NCNT with different pyrolysis temperature 900~1100 ℃.


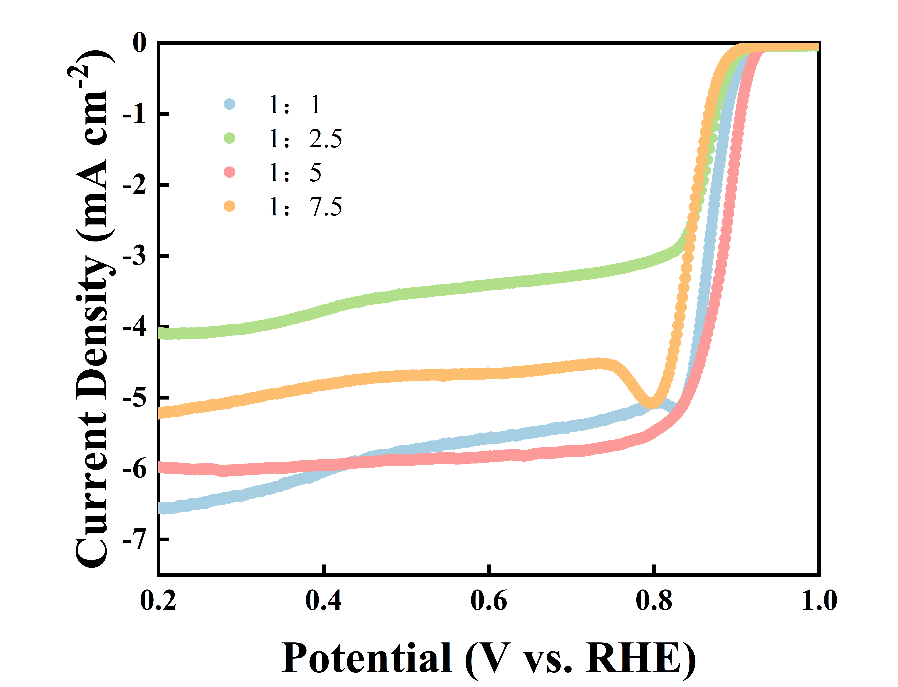


Figure S32. LSV polarization curves of FePc-Fe-NCNT with different FePc loadings (the mass ratios of Fe-N_4_-NCNT to FePc are 1:1, 1:2.5, 1:5 and 1:7.5, respectively).


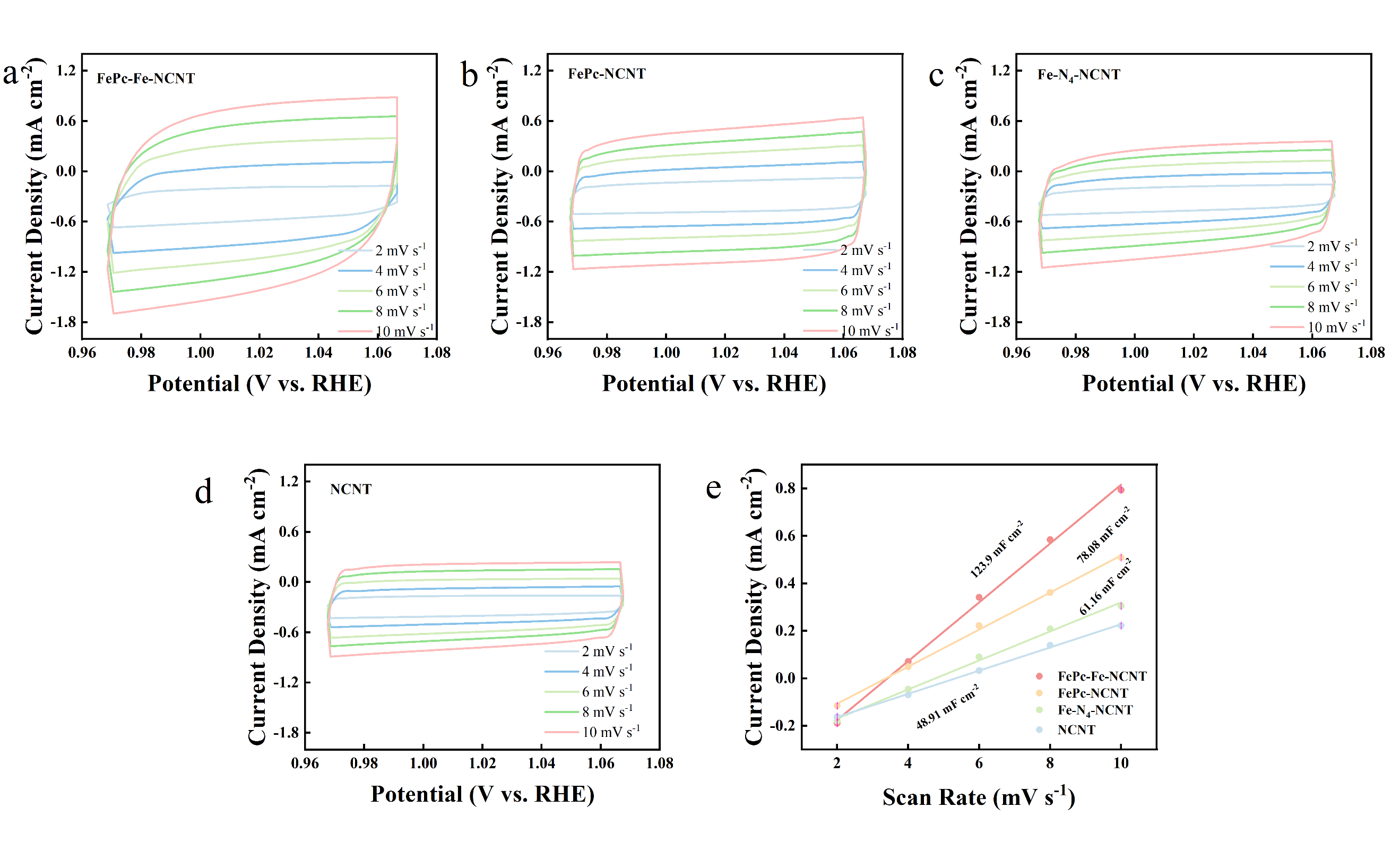


Figure S33. CV curves of different catalysts in O_2_-saturated 0.1 M KOH at different scan rates (2-10 mV s^−1^) in 1.08-1.13V vs RHE for ORR (a-d), and corresponding electrochemical double-layer capacitance (cdl) (e).


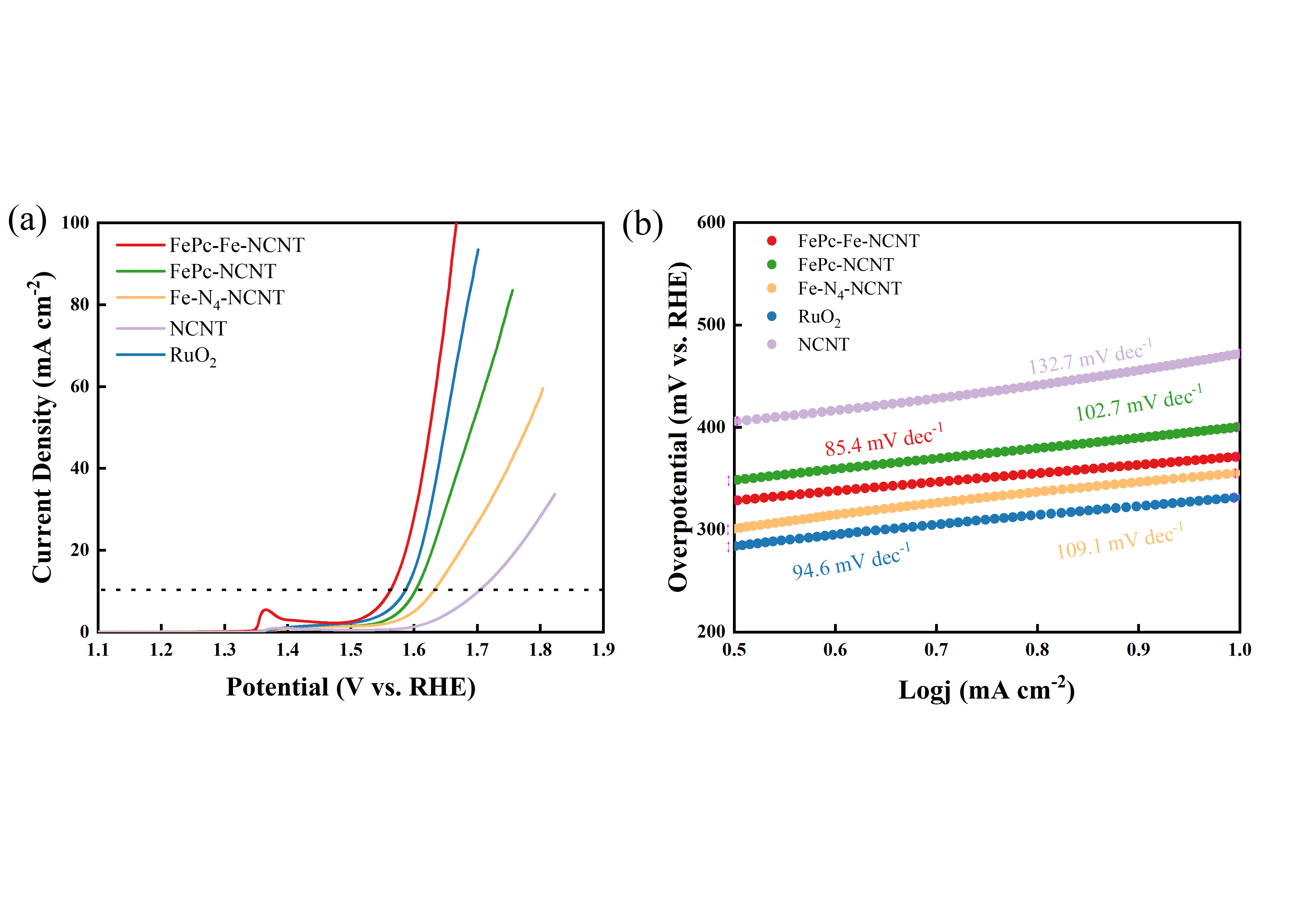


Figure S34. (a) OER LSV curves measured in 0.1 M KOH, and (b) the corresponding OER Tafel plots.


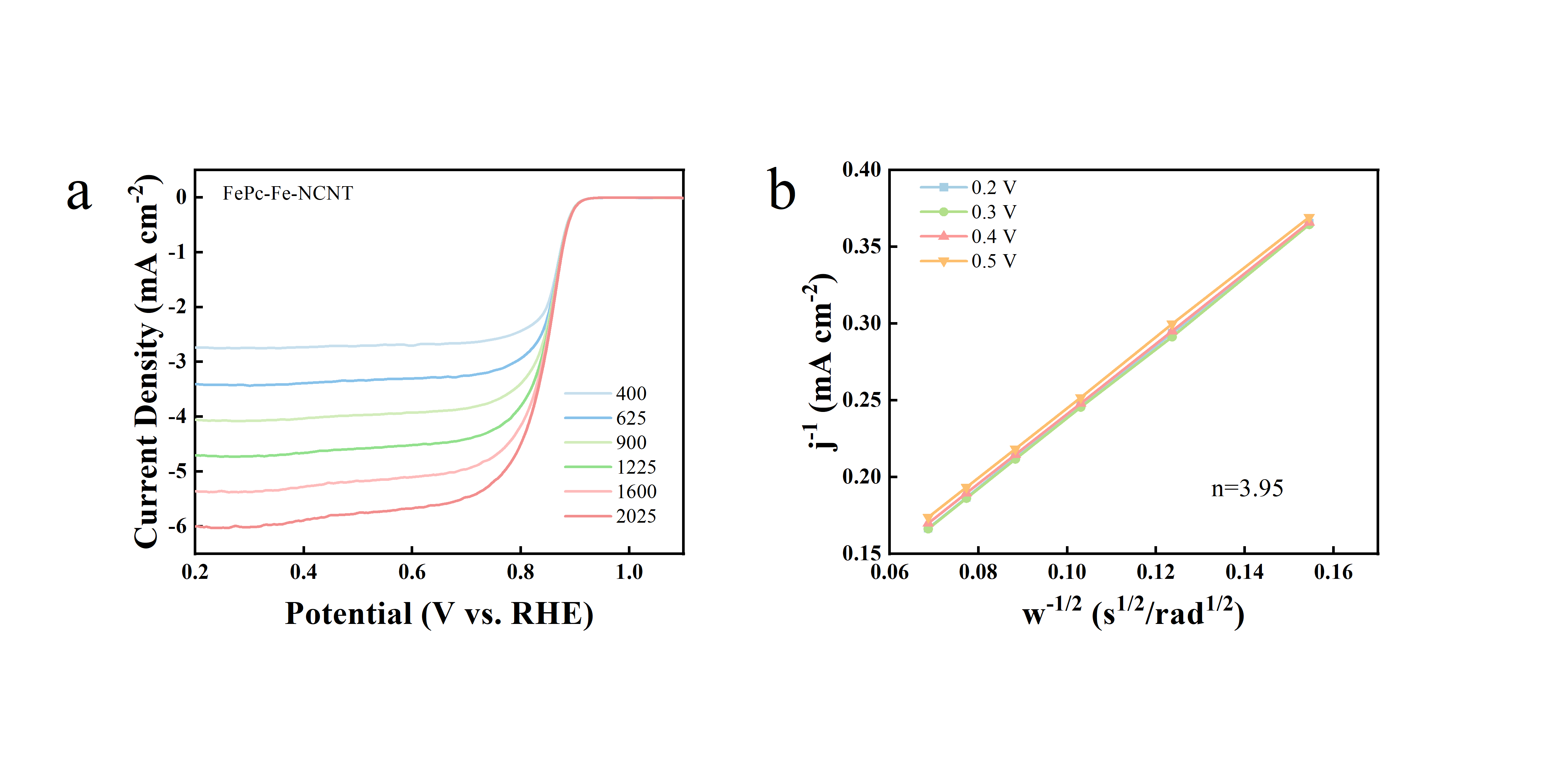


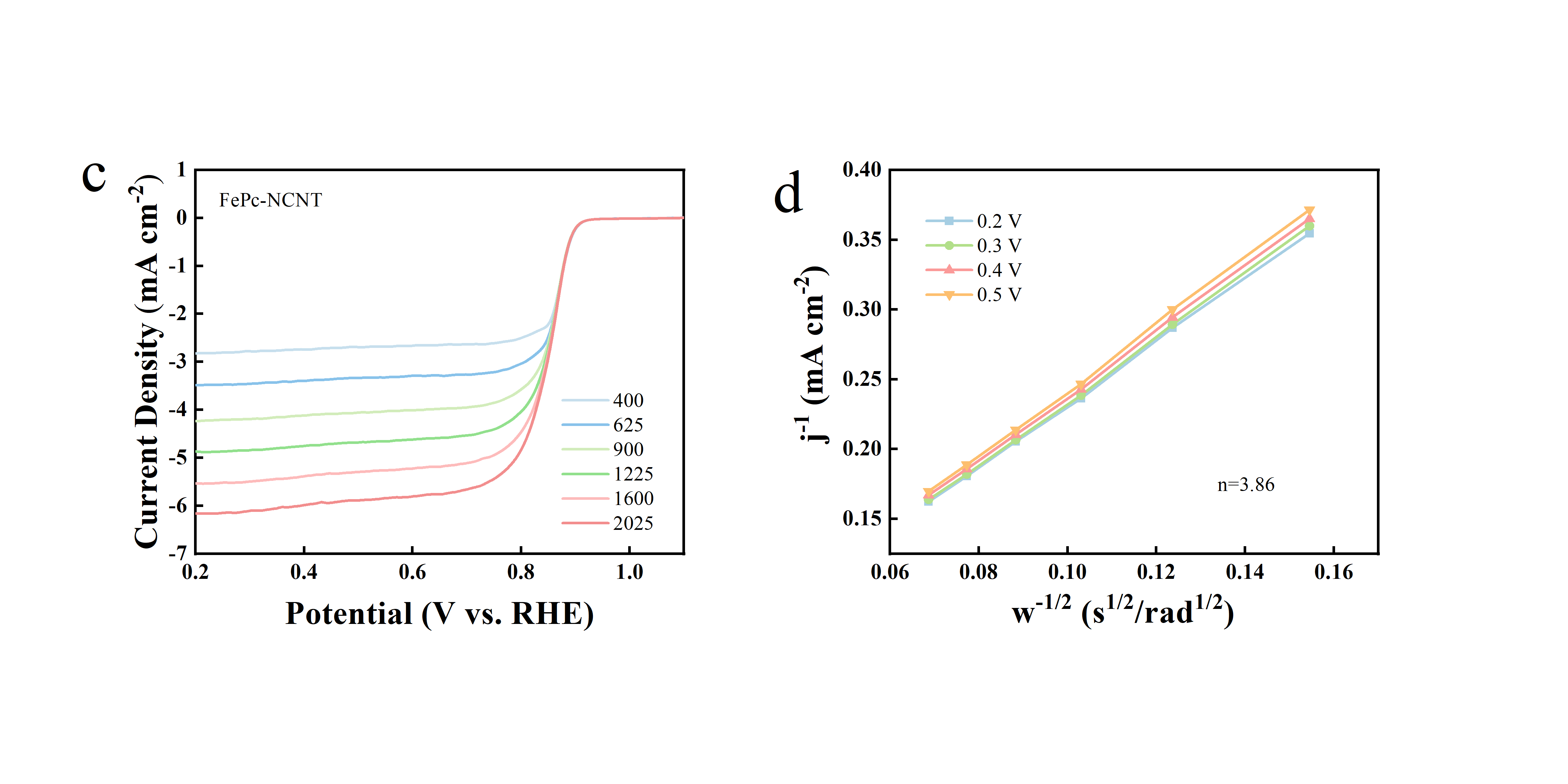


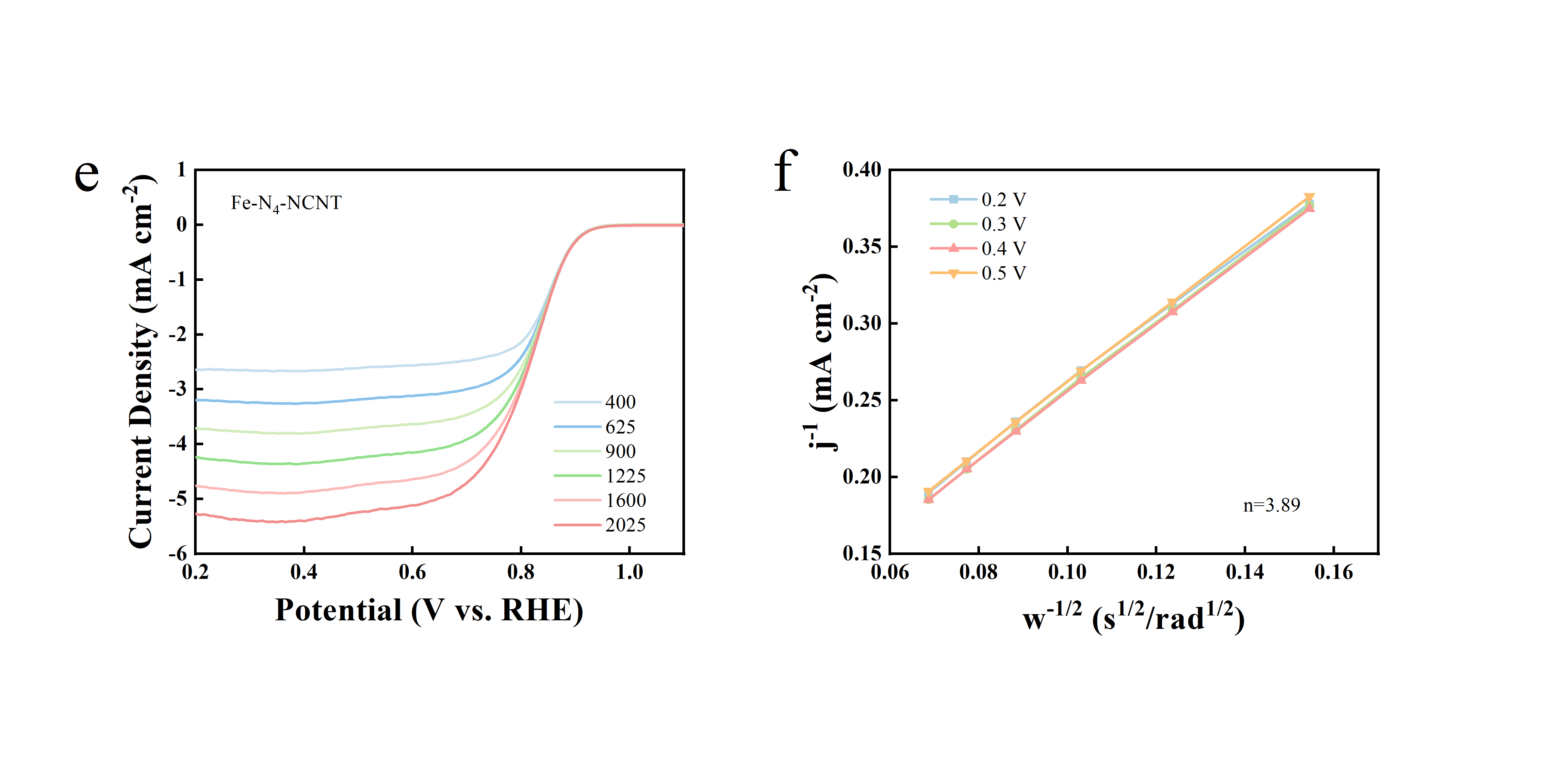


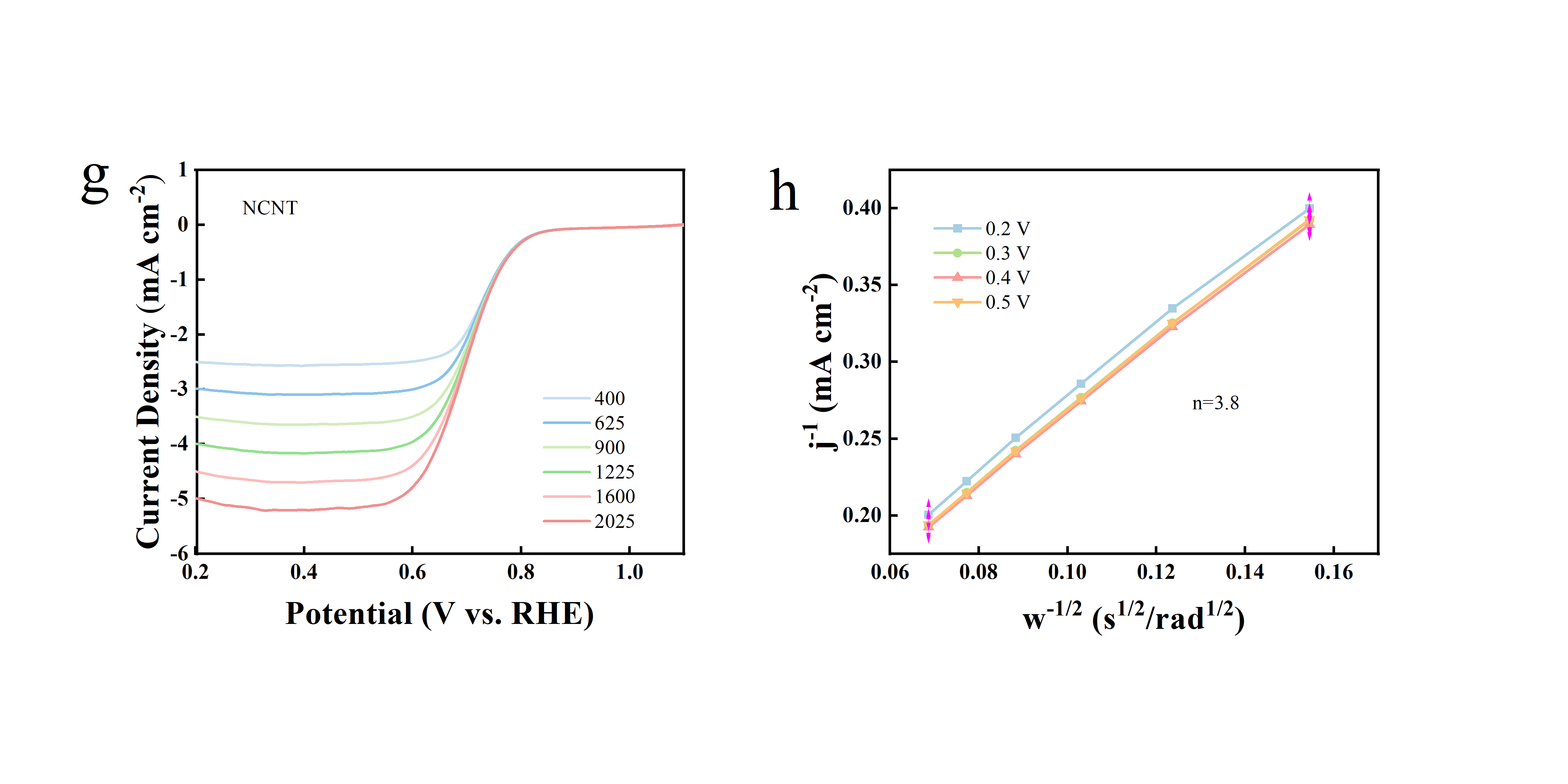


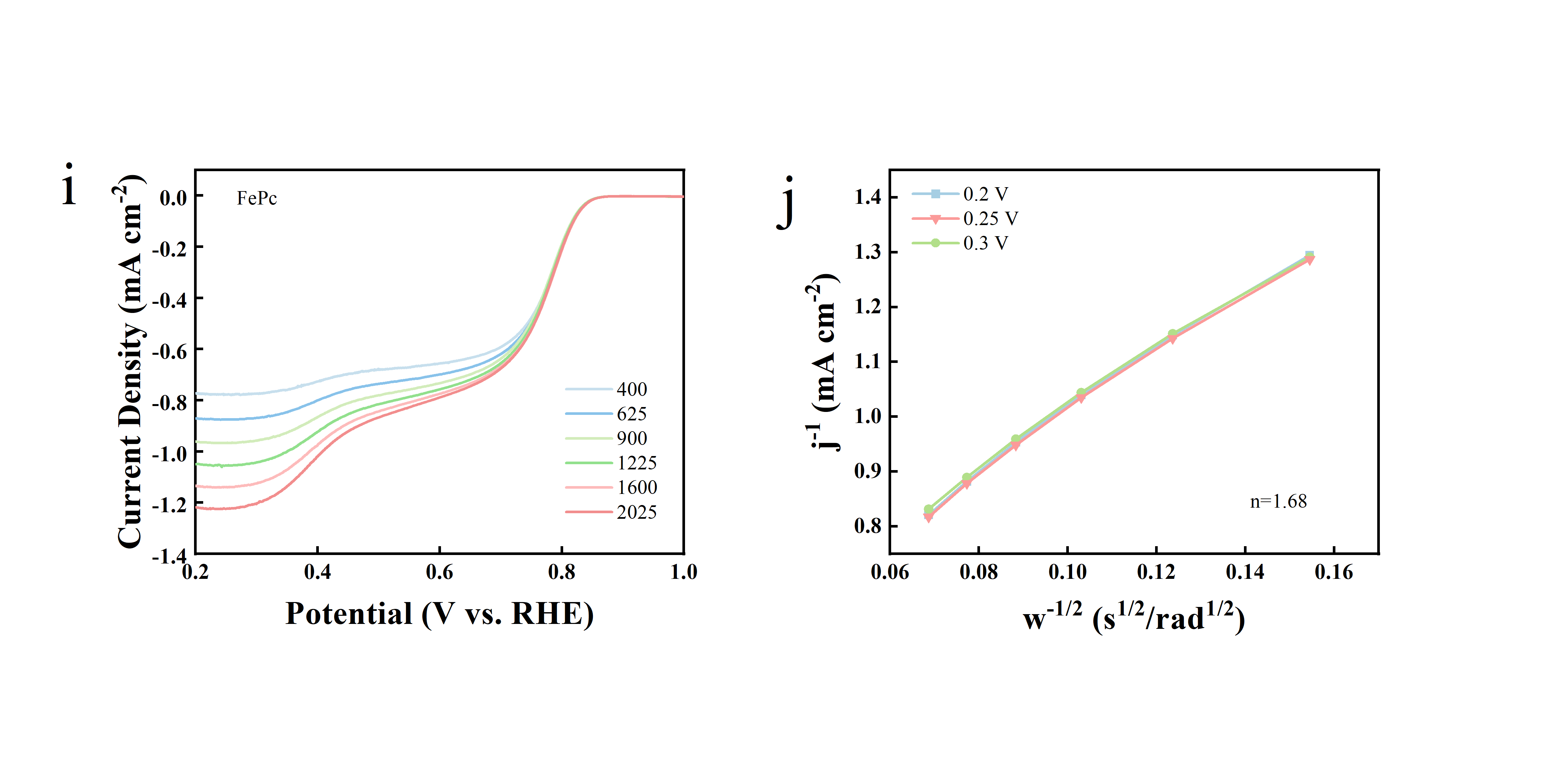


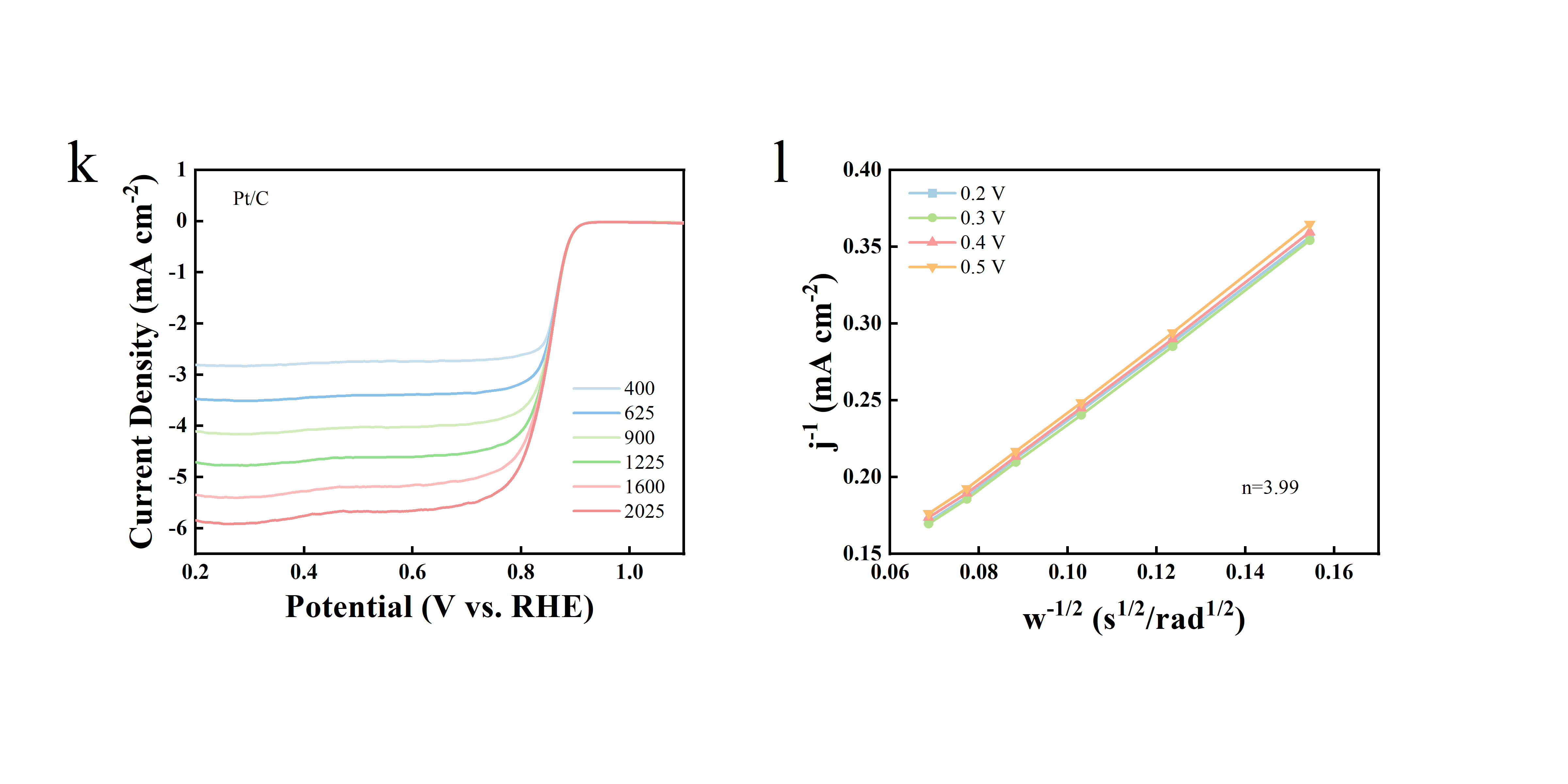


Figure S35. The ORR polarization curves of FePc-Fe-NCNT (a, b); FePc -NCNT (c, d); Fe-N_4_-NCNT (e, f); NCNT (g, h); FePc (i, j) and Pt/C (k, l) at different rotating rates with K-L plots and electron transfer number.


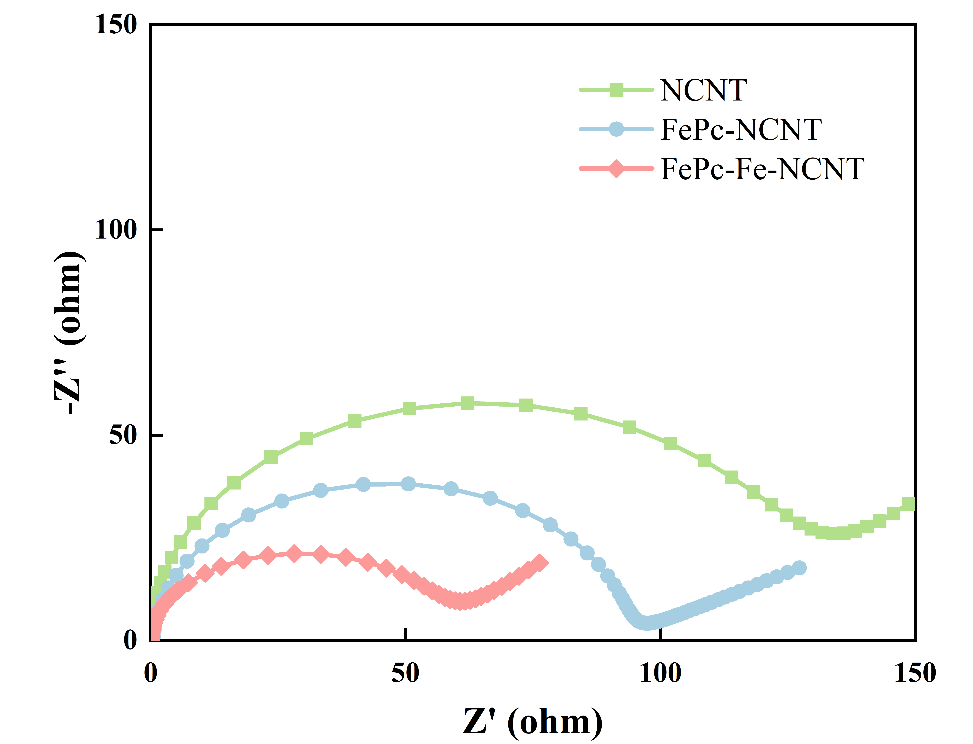


Figure S36. Nyquist plots at 0.6 V vs. RHE in O_2_ saturated electrolyte at 1600 rpm.


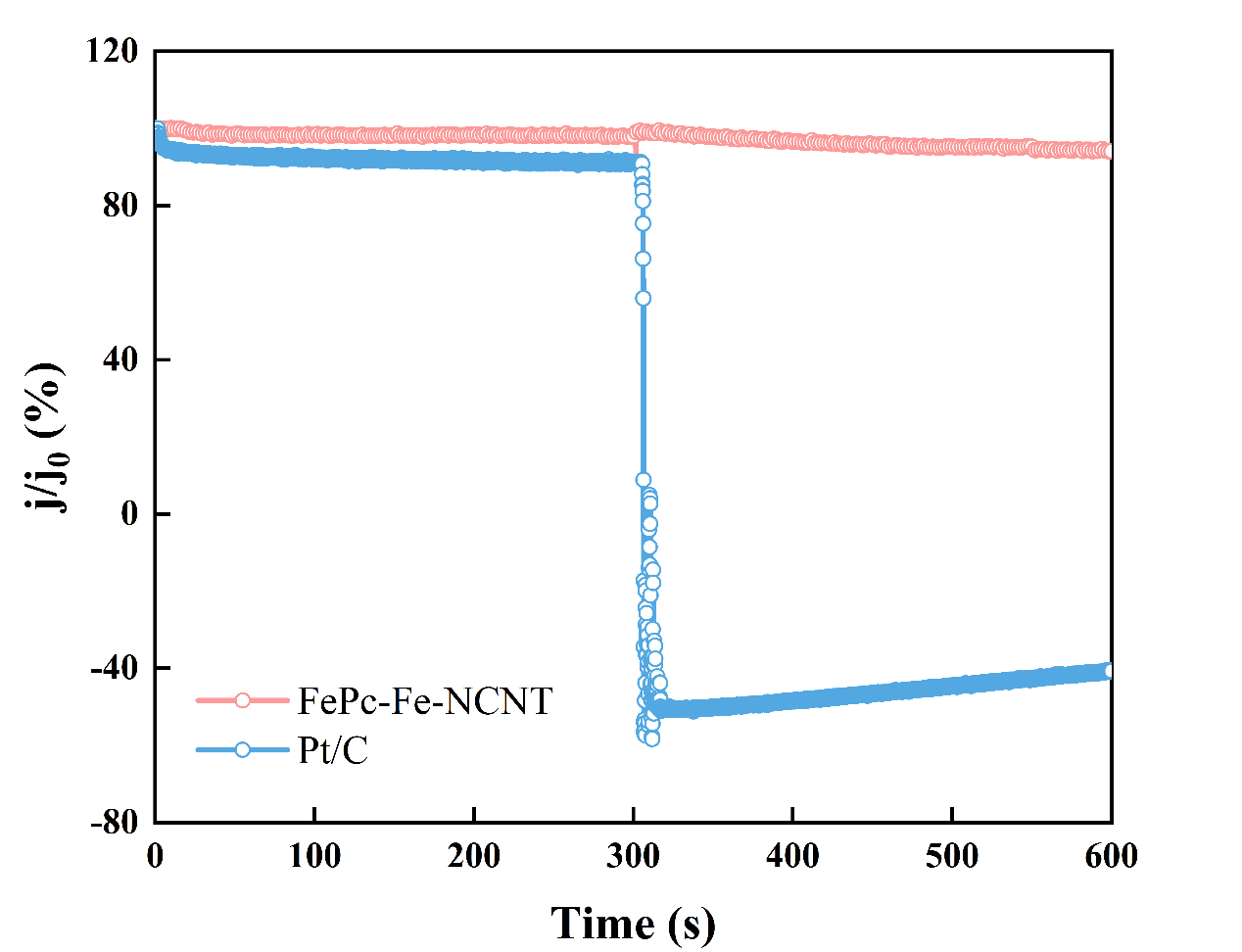


Figure S37. The i–t curves of FePc-Fe-NCNT and commercial Pt/C before and after the addition of methanol.


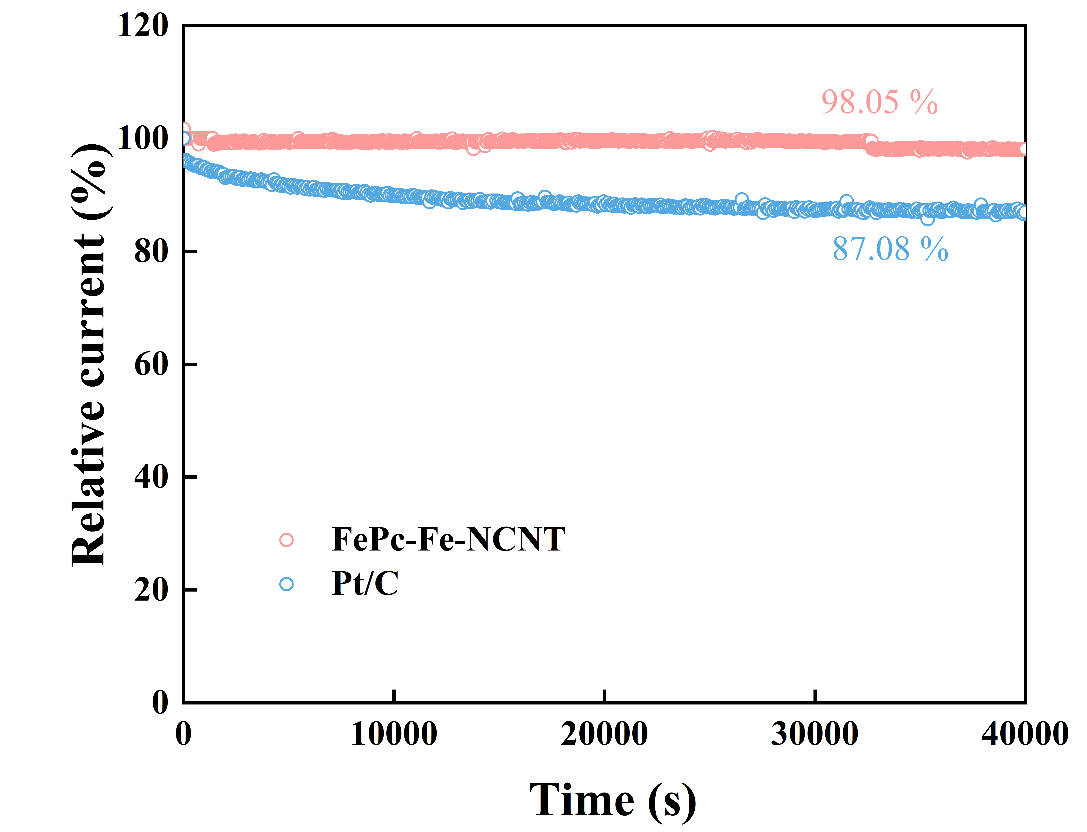


Figure S38. Chronoamperometric curves of FePc-Fe-NCNT and Pt/C.


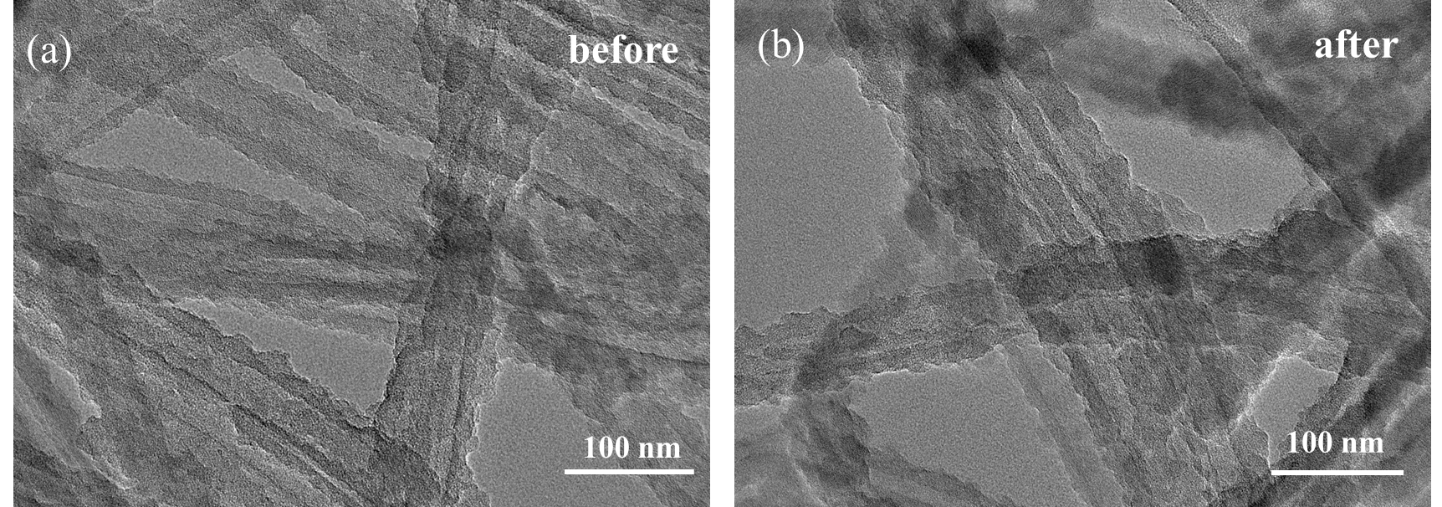


Figure S39. TEM of FePc-Fe-NCNT (a, b) after ORR long term test.


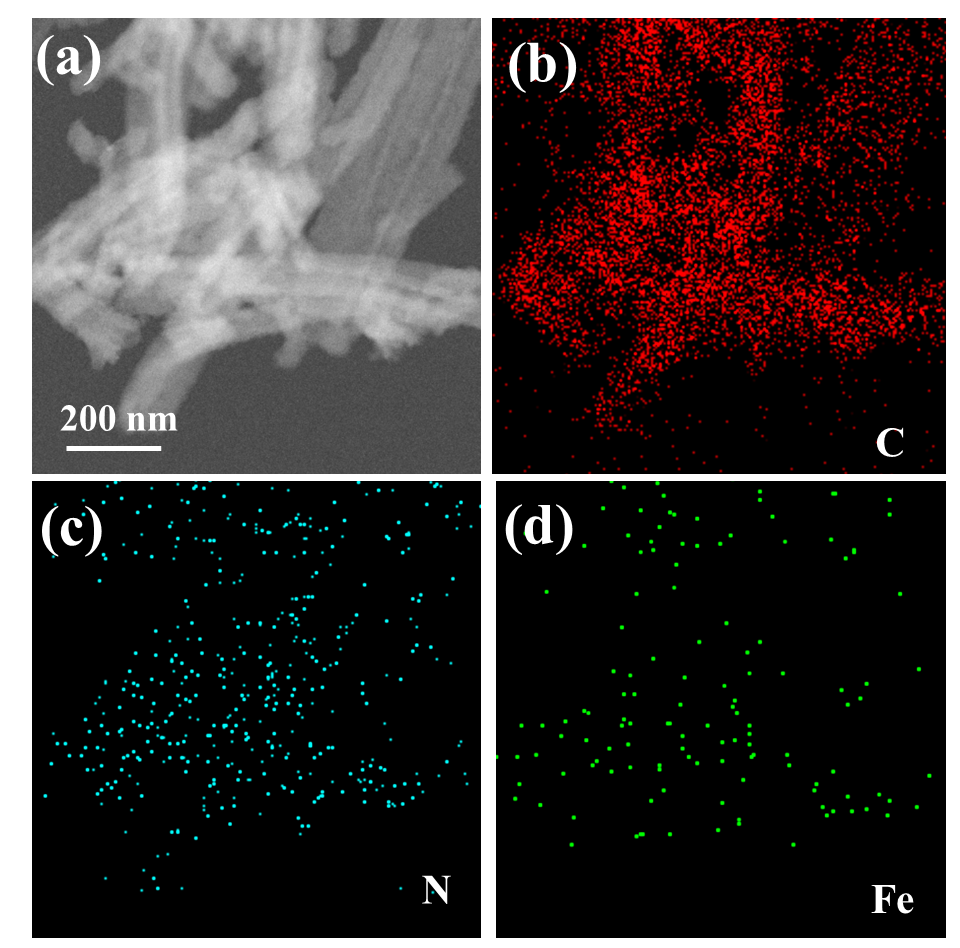


Figure S40. (a) STEM and (b-d) mapping of FePc-Fe-NCNT after stability test.


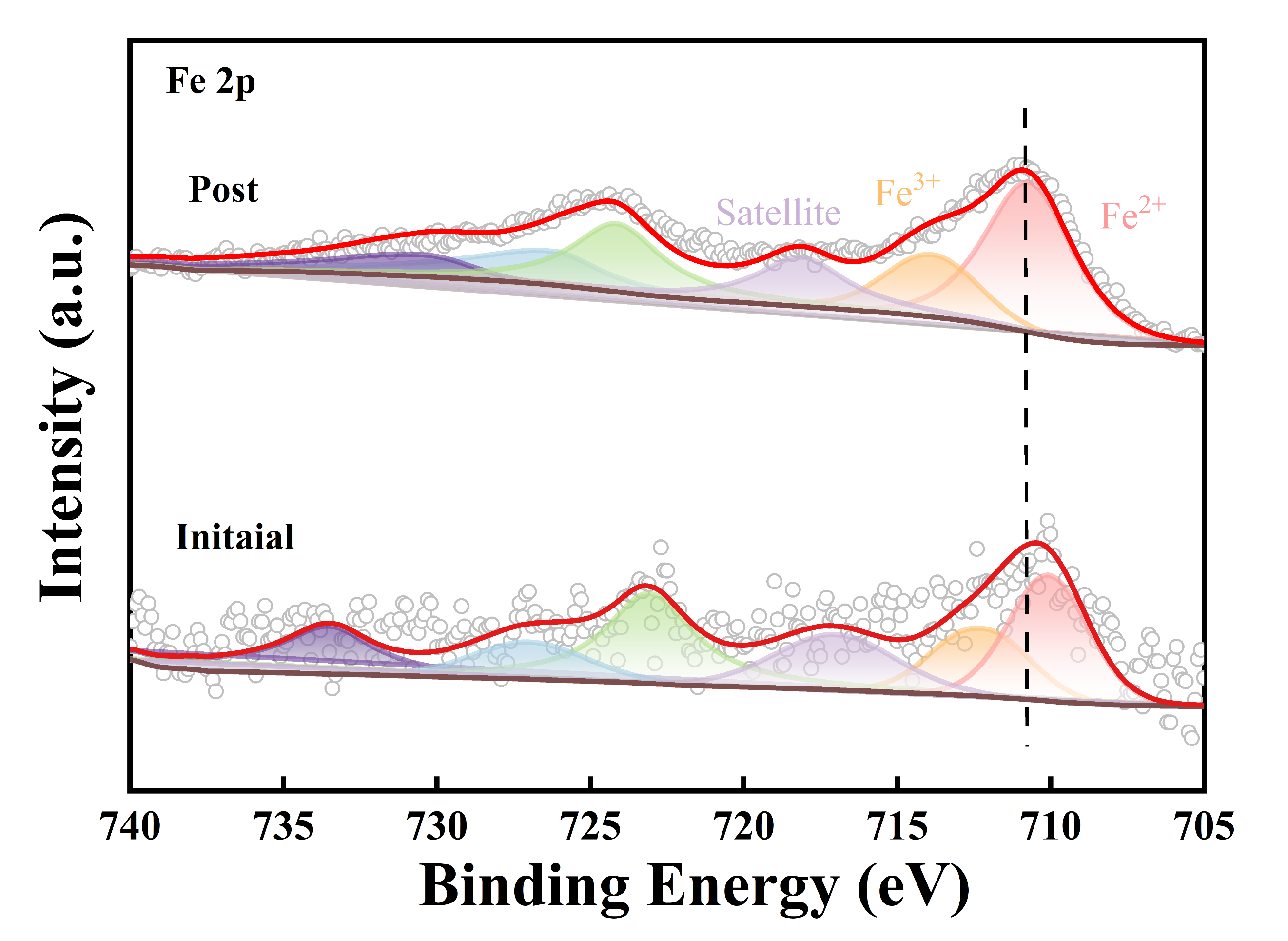


Figure S41. The high-resolution XPS Fe 2p spectra of initial and post-ADT FePc-Fe-NCNT.


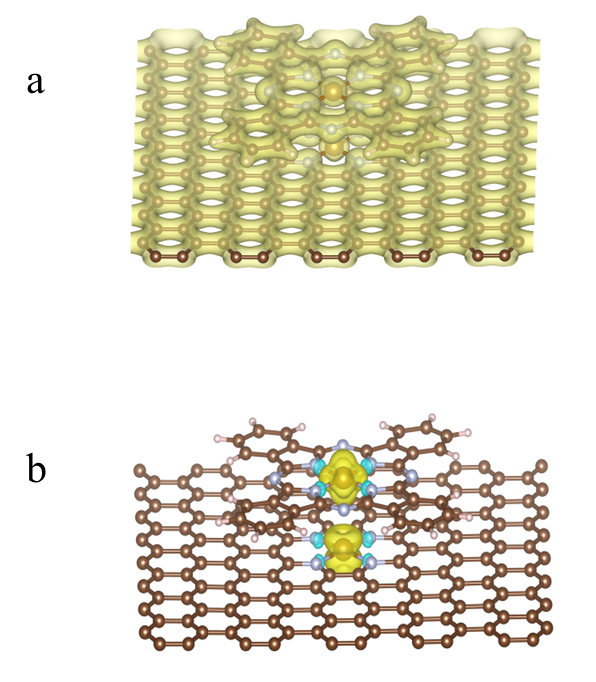


Figure S42. (a) Total charge density of FePc-Fe-NCNT; (b) Spin density plot of FePc-Fe-NCNT ((yellow and green iso-surfaces are positive and negative spin density, respectively, isosurface = 0.007 a.u.).


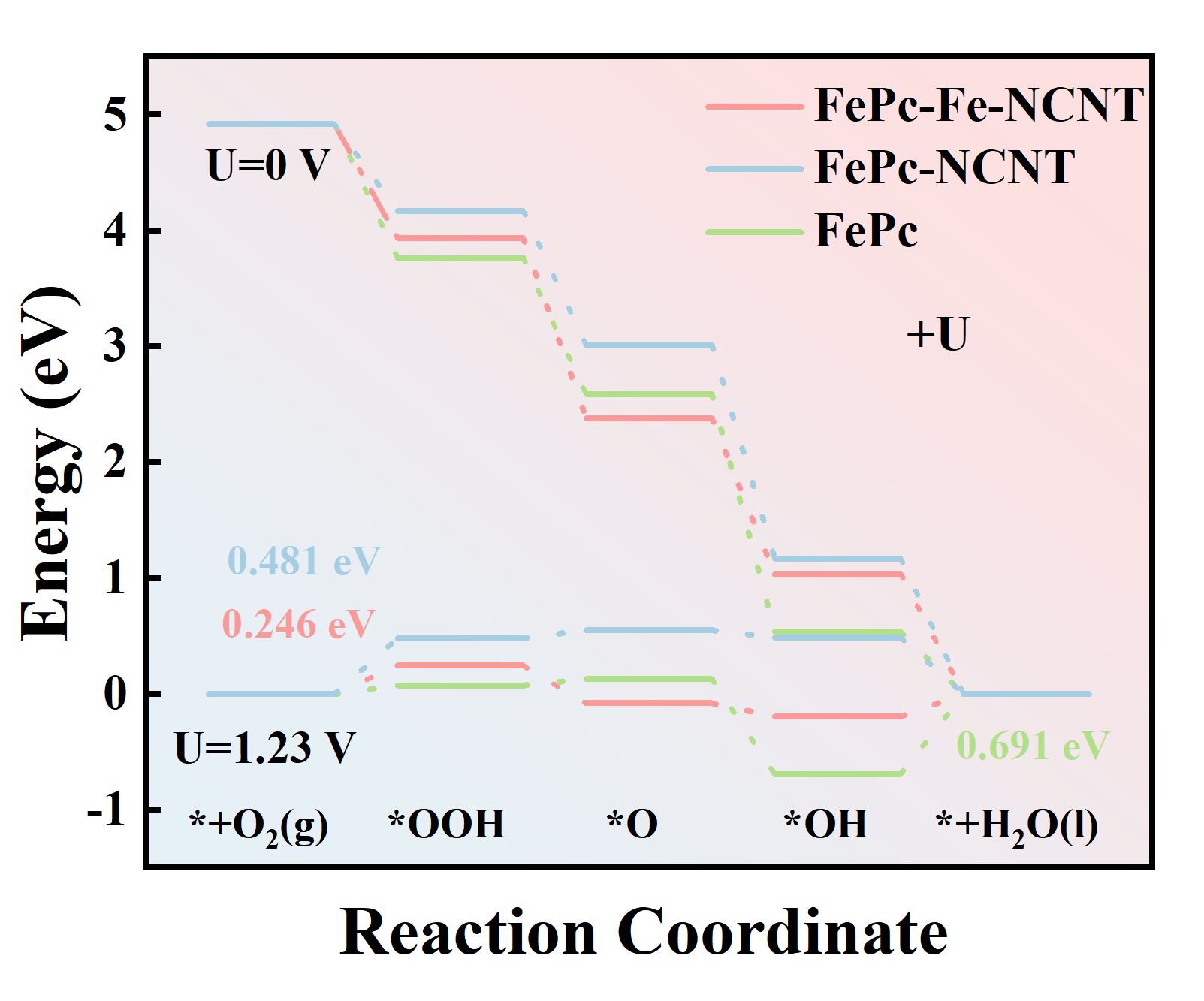


Figure S43. Free-energy diagram of ORR with U at U =0 V /1.23 V of FePc-Fe-NCNT, FePc-NCNT and FePc.


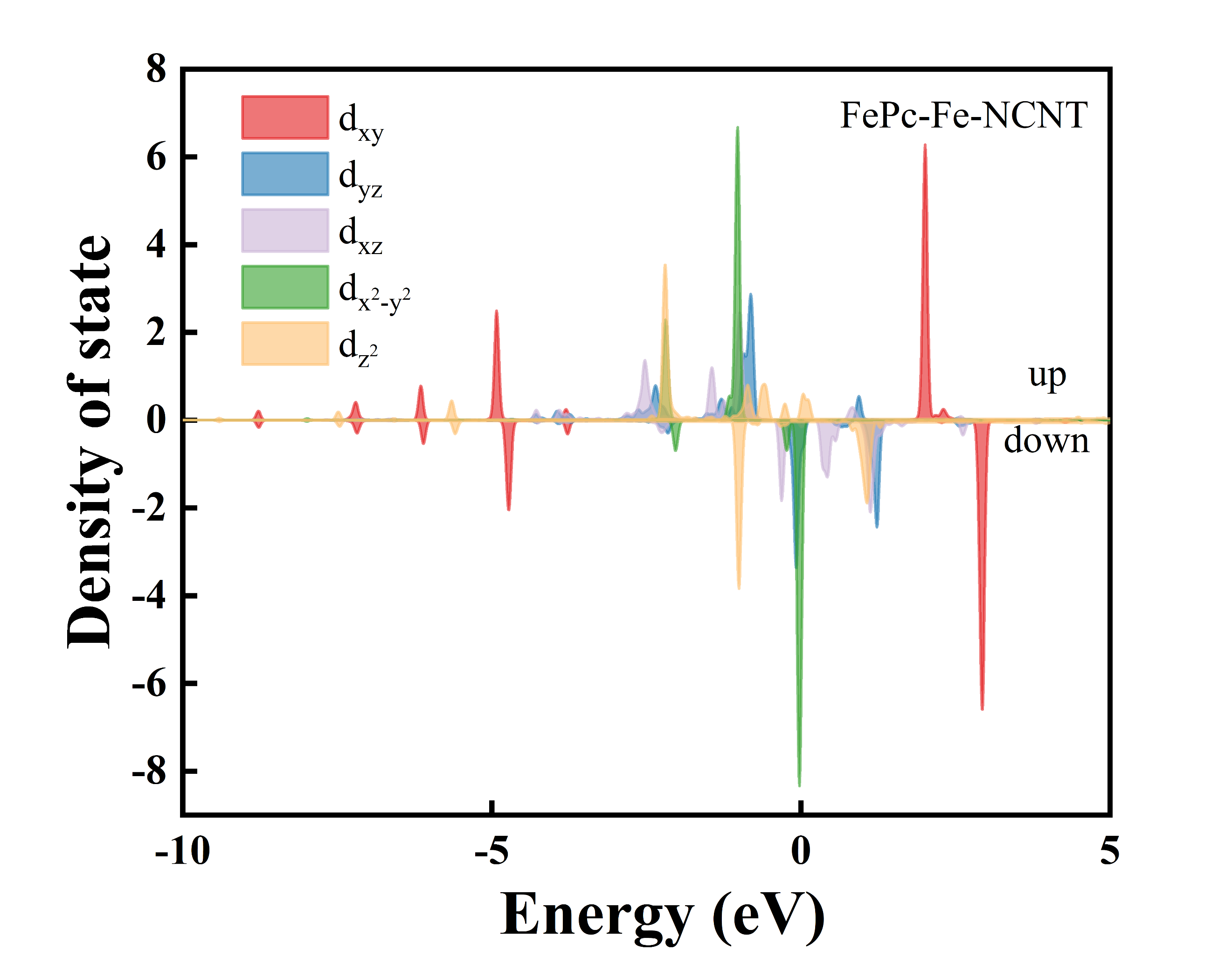


Figure S44. The projected density of states (DOS) for FePc-Fe-NCNT.


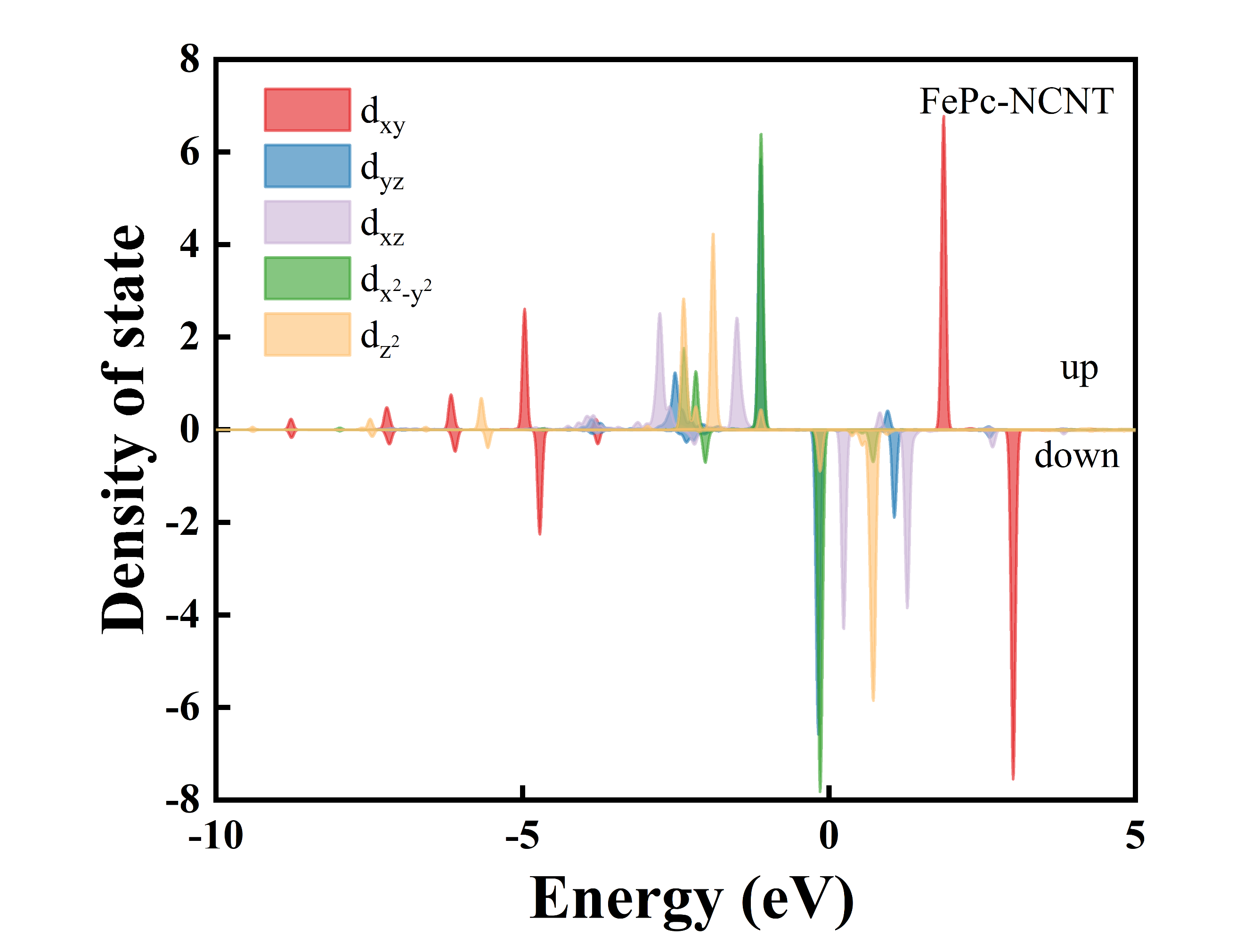


Figure S45. The projected density of states (DOS) for FePc-NCNT.


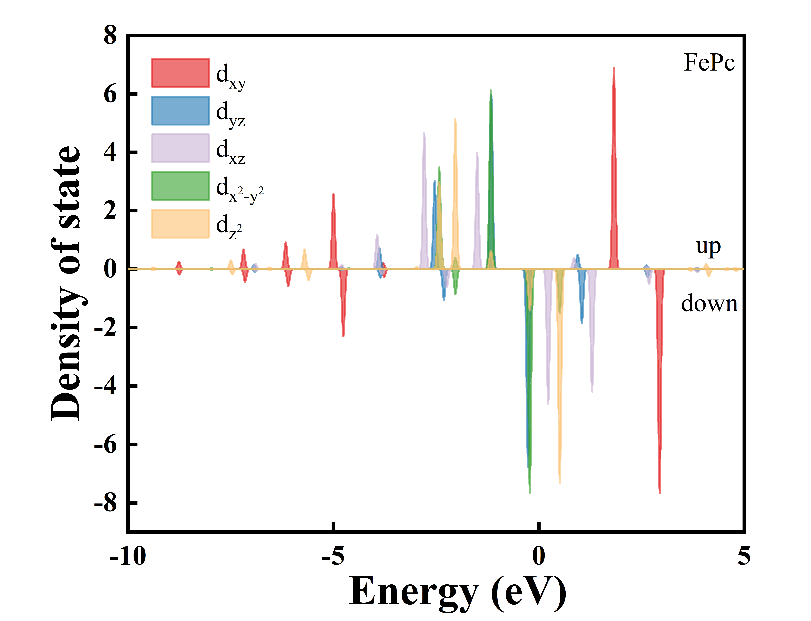


Figure S46. The projected density of states (DOS) for FePc.


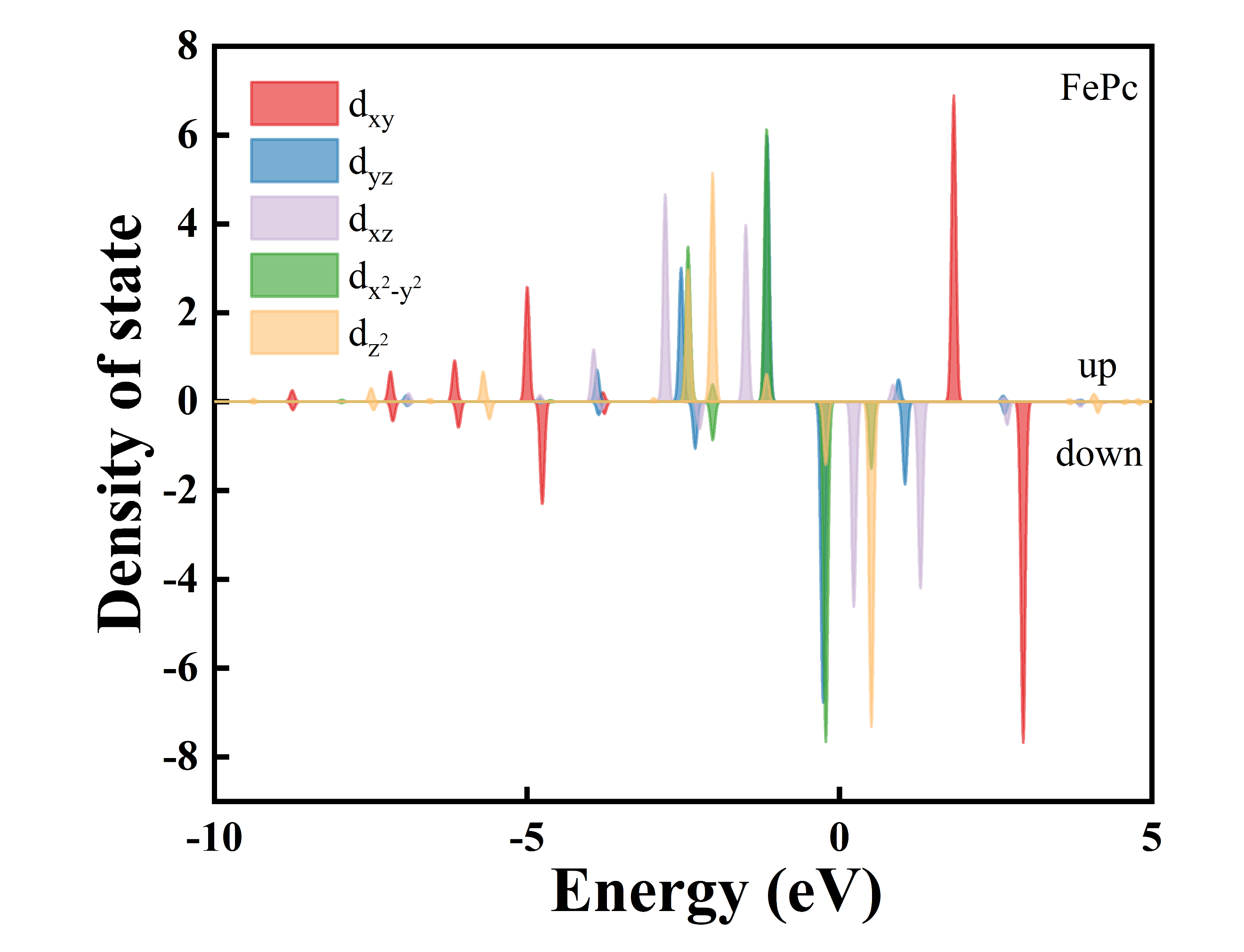


Figure S47. Projected density of states (pDOS) of Fe and O over FePc.


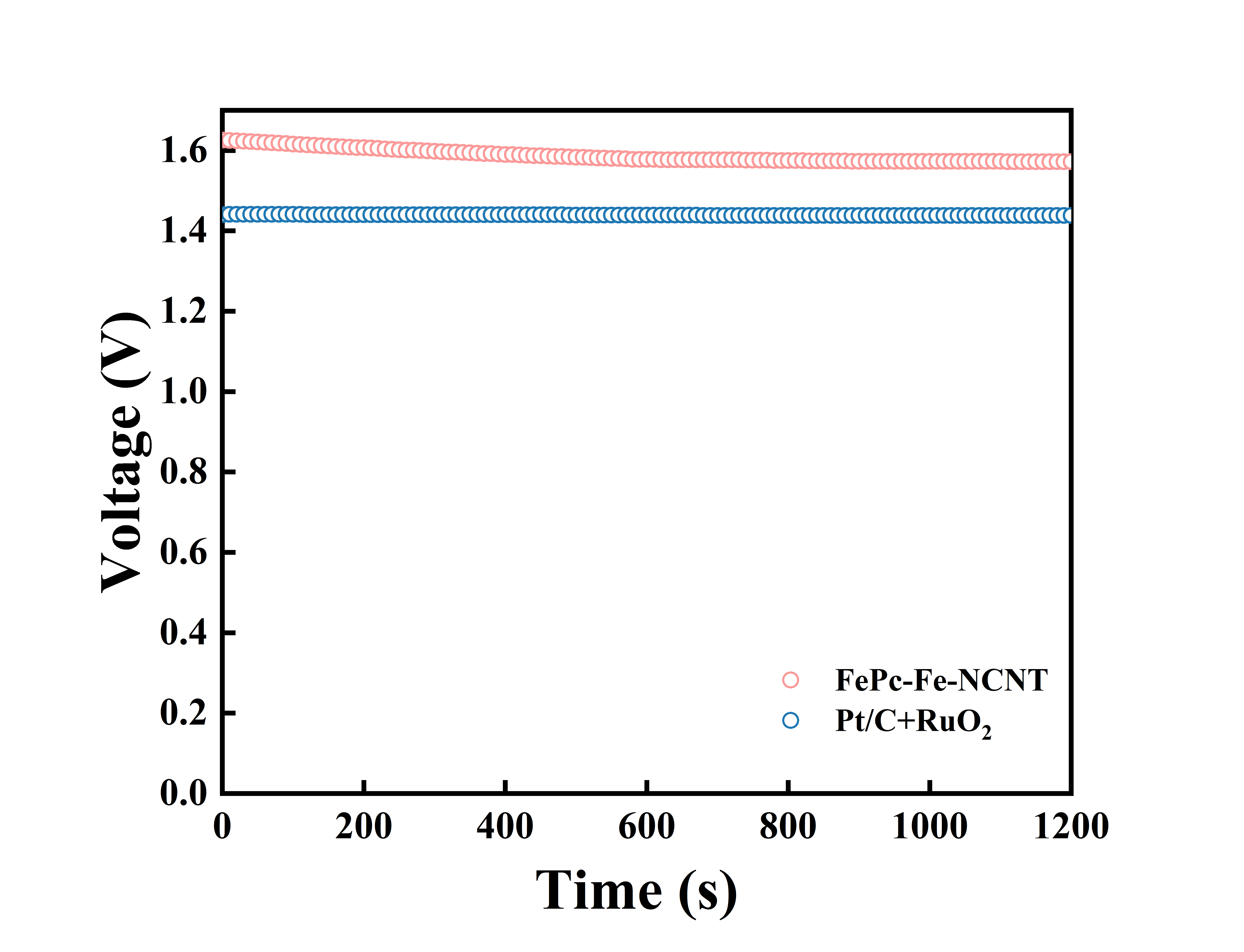


Figure S48. Open-circuit voltage (OCV) of the liquid ZABs using FePc-Fe-NCNT and Pt/C + RuO_2_ catalysts.


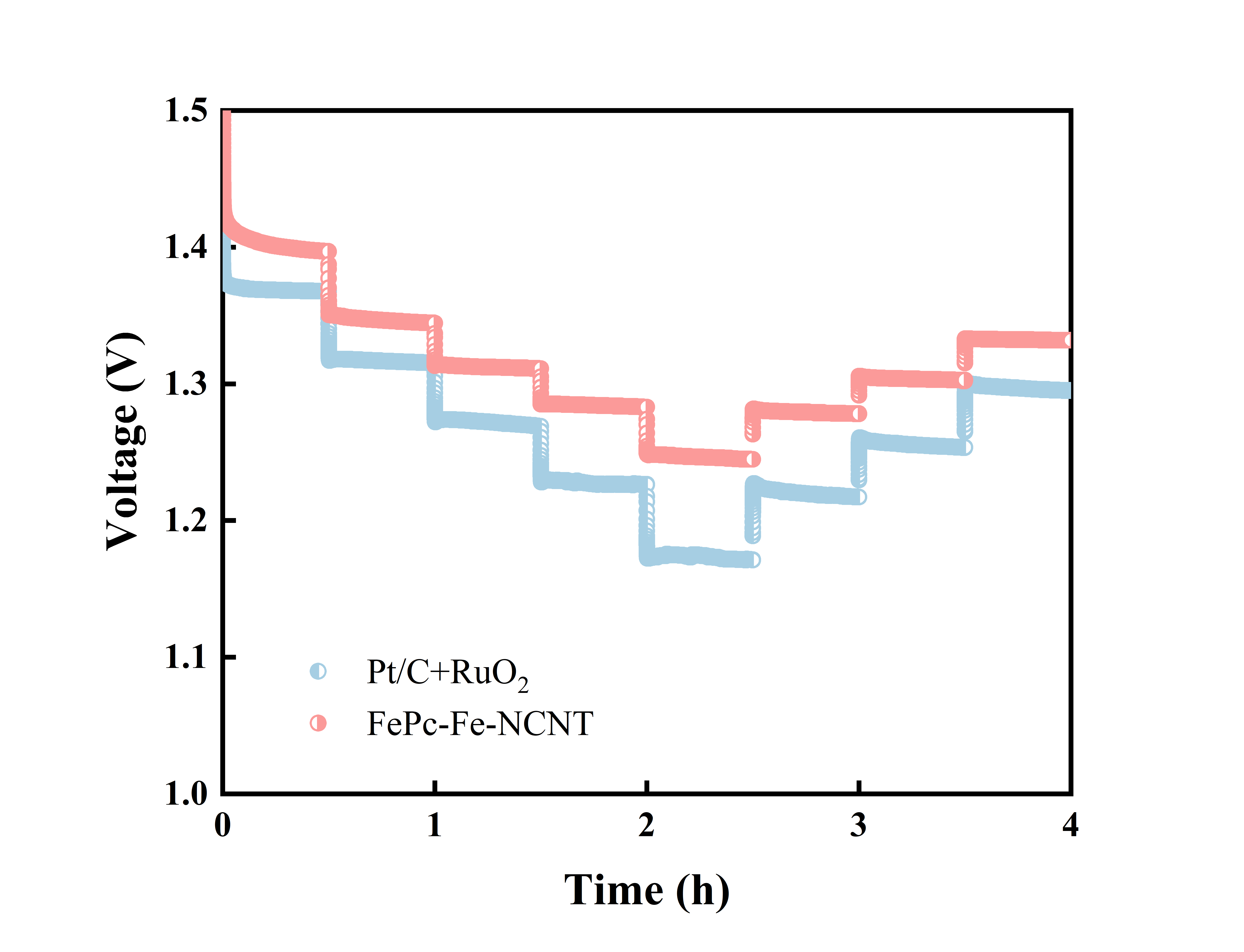


Figure S49. Rate capability test of the Zn–air battery with FePc-Fe-NCNT and Pt/C+RuO_2_ catalysts at different current densities.


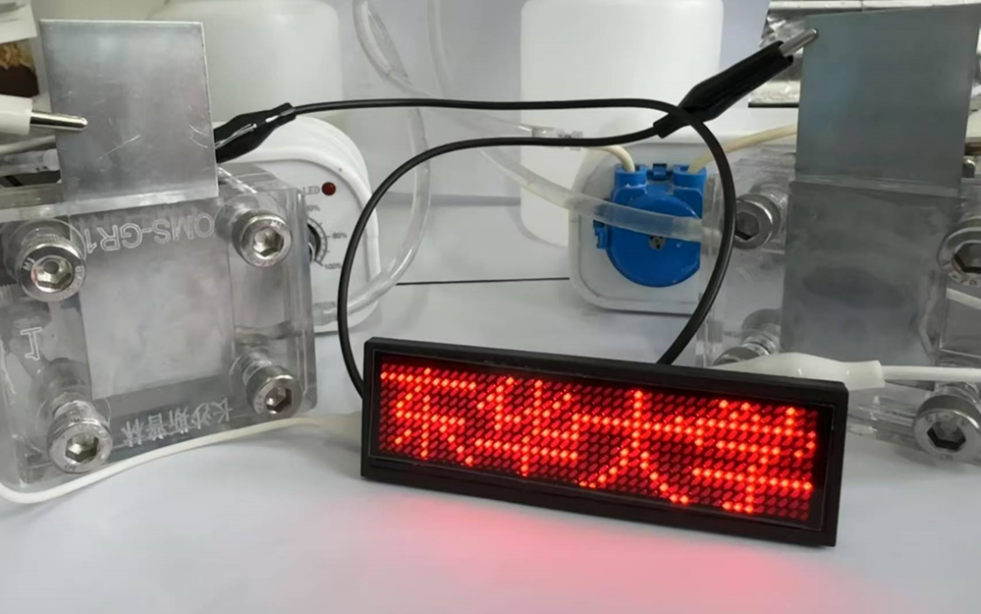


Figure S50. Photograph of an LED powered by recharge ZAB utilizing FePc-Fe-NCNT catalyst.


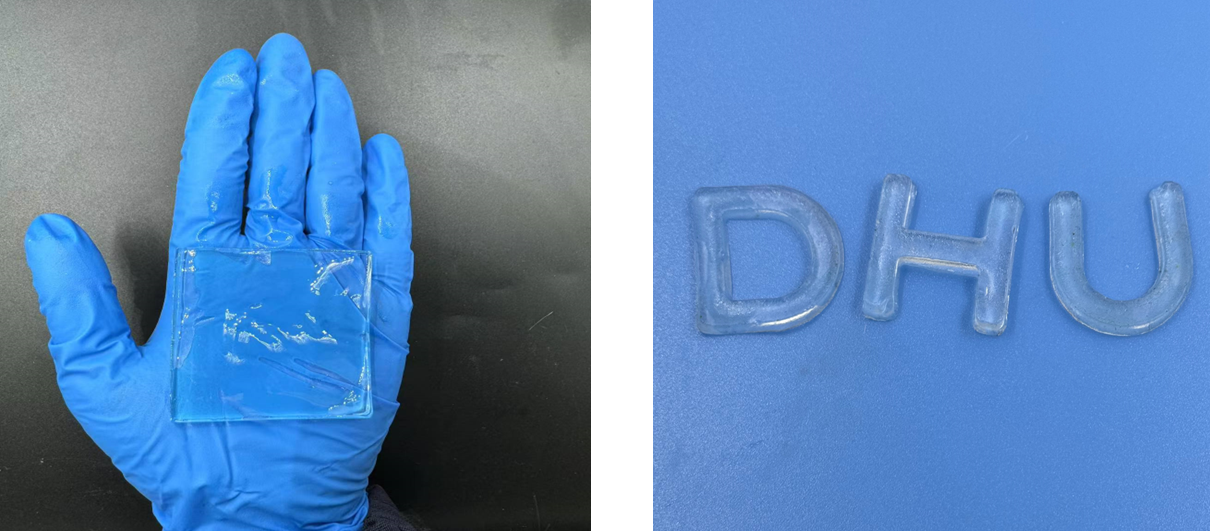


Figure S51. Optical photograph of the QSS-DMSO hydrogel electrolyte


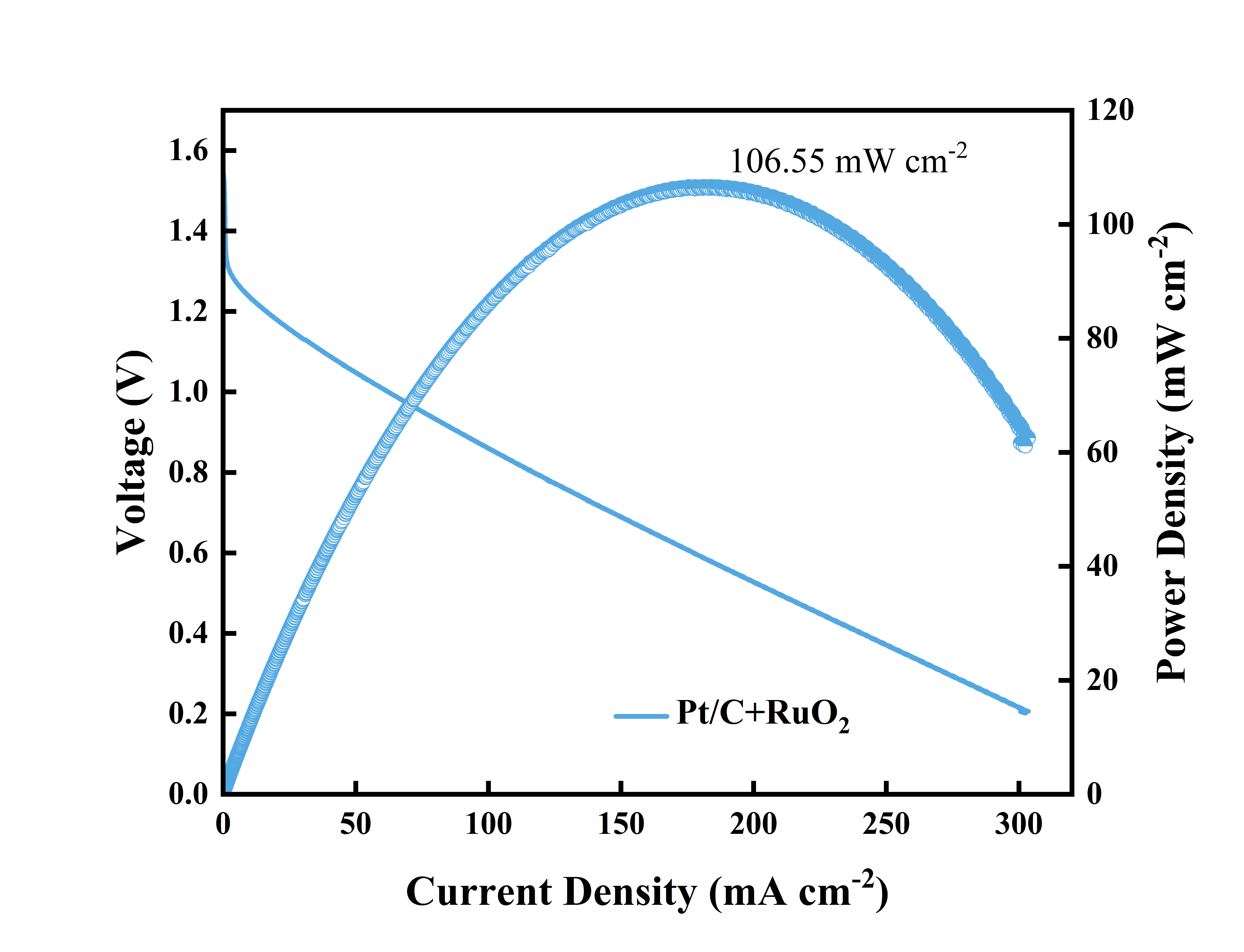


Figure S52. Polarization and power density curves of Zn–air batteries using Pt/C+RuO_2_.


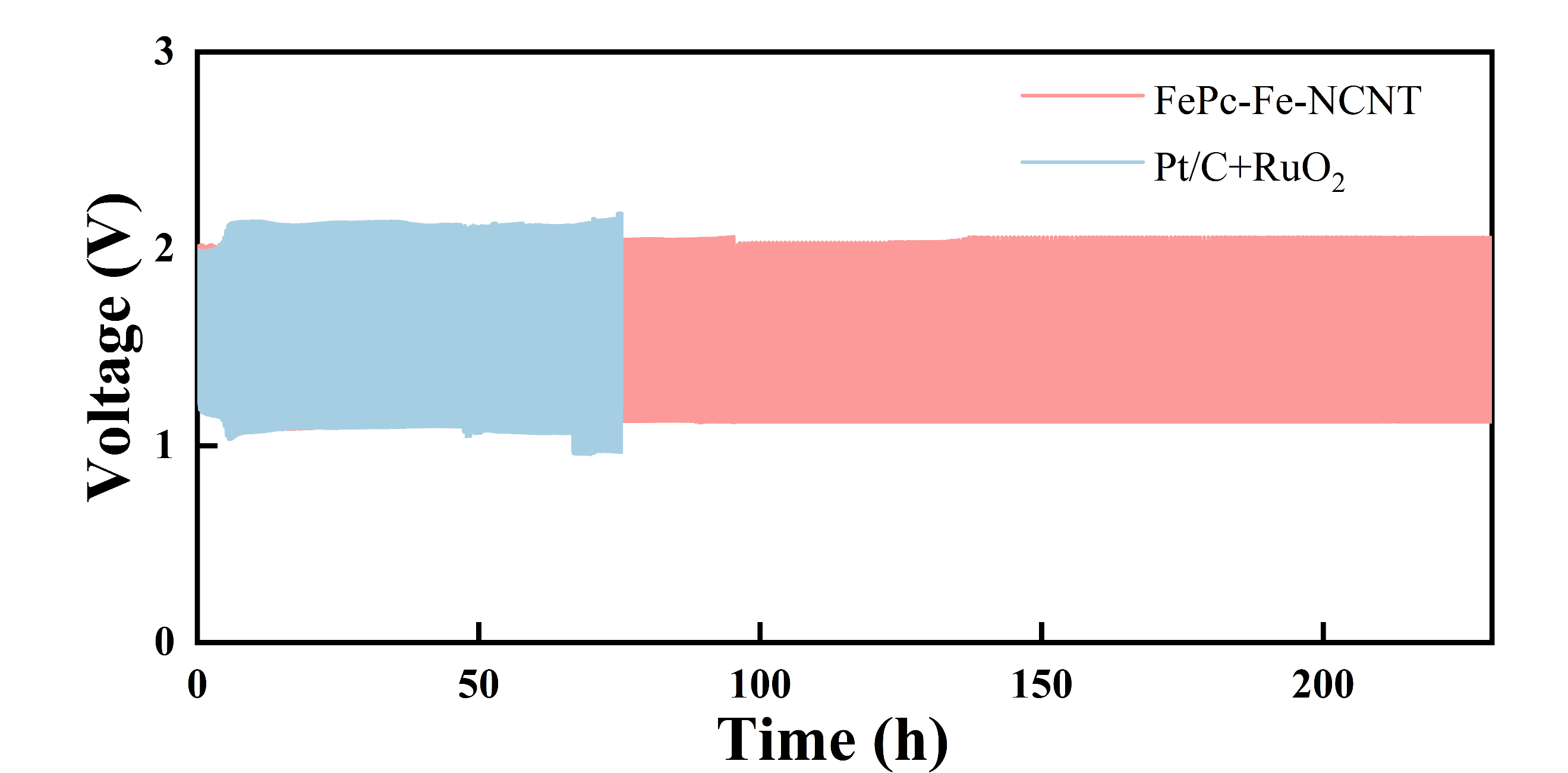


Figure S53. Charge/discharge curves of the FePc-Fe-NCNT and Pt/C+RuO_2_ based ZABs at 5 mA cm^−2^.


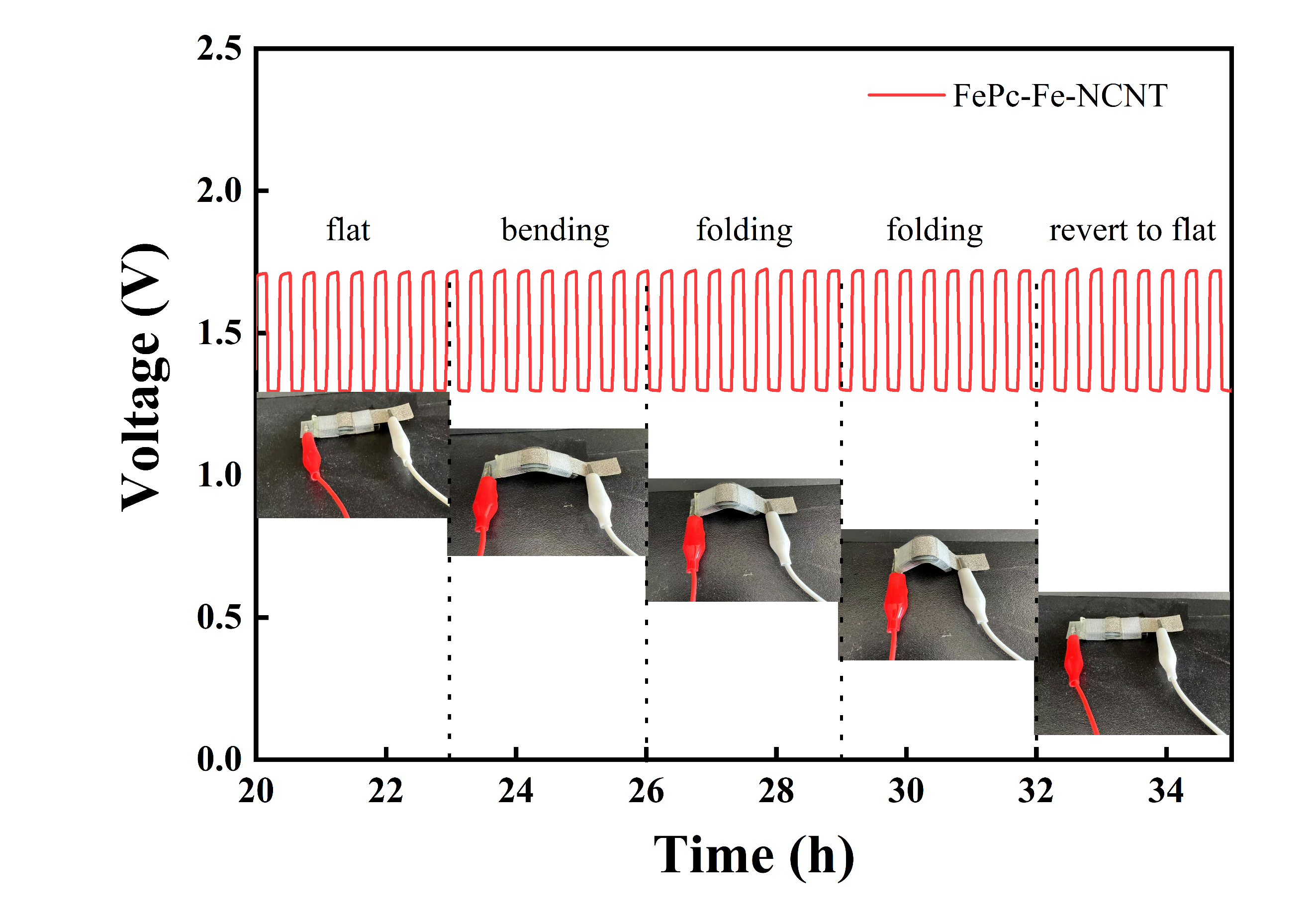


Figure S54. Galvanostatic charge/discharge curve of the QSS-ZABs assembled FePc-Fe-NCNT under various mechanical deforms.


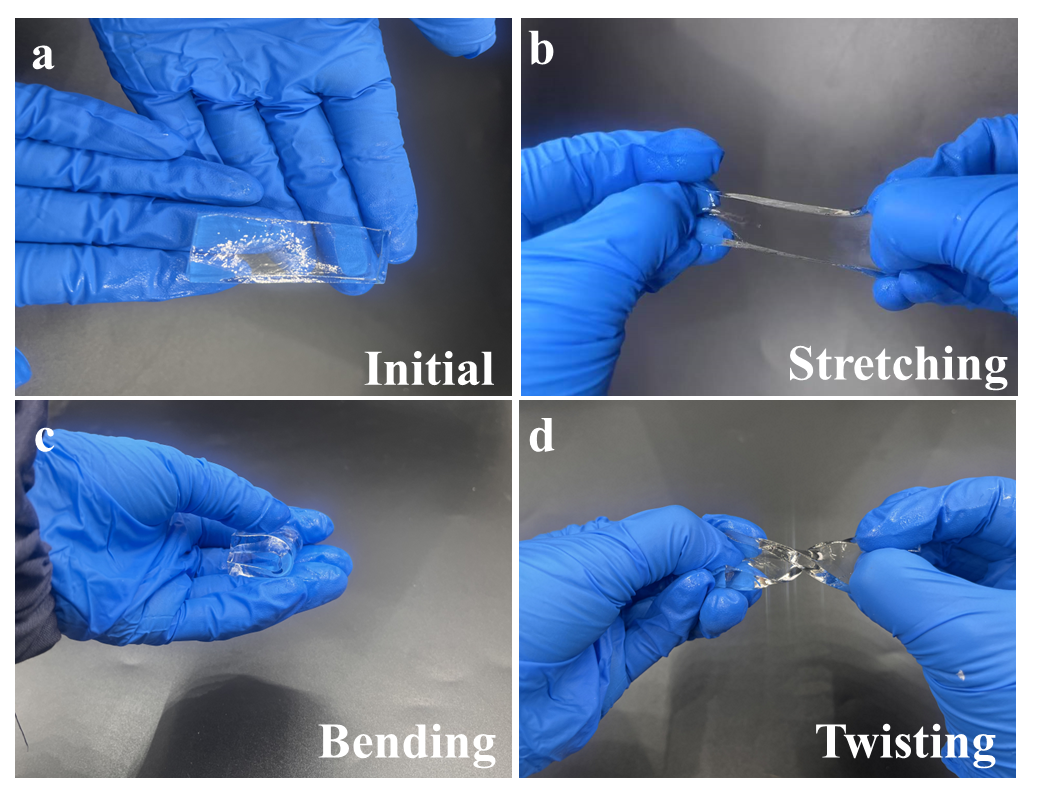


Figure S55. Photos of (a)initial, (b) stretching, (c) bending and (d) twisting capability for PAM-DMSO GPE.

As illustrated in Figure S55, PAM-DMSO GPE was subjected to arbitrary stretching, bending, and twisting without any surface fracturing, proving its potential for real-world applications under extreme mechanical conditions. It demonstrated the superior mechanical performance and stability of PAM-DMSO GPE.


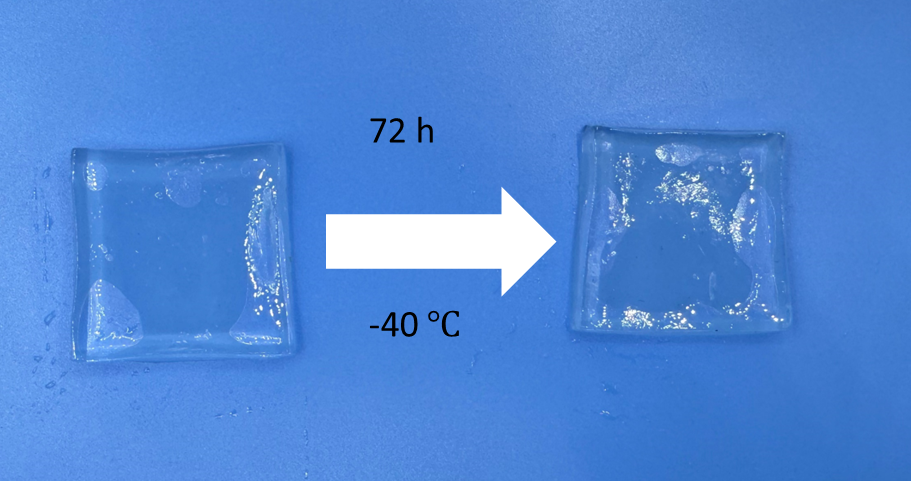


Figure S56. The photograph of the PAM-DMSO GPE at (a) 25 °C, and (b) –40 °C.

As shown in Figure S56, the surface of PAM/DMSO GPE did not exhibit noticeable ice crystal formation at the low temperature of −40 °C.

Table S1. The Fe content for different samples tested by ICP-OES.

| Sample | Content (wt%) |
| --- | --- |
| FePc-Fe-NCNT | 1.56 % |
| FePc-NCNT | 1.2 % |

Table S2. Deconvolution results of high resolution XPS spectra for N1s.

| Sample | Relative ratios of the deconvoluted N species (%) | | | |  |
| --- | --- | --- | --- | --- | --- |
|  | Pyridinic N | Graphitic N | Oxidized N | Fe-N | pyrrolic N |
| FePc-Fe-NCNT | 20.42 | 34.50 | 25.33 | 19.75 | - |
| FePc-NCNT | 14.97 | 14.78 | 35.29 | 34.96 | - |
| Fe-N_4_-NCNT | 31.72 | 31.05 | 23.47 | 13.76 | - |
| NCNT | 37.96 | 38.78 | 23.26 | - | - |
| FePc | 77.90 | - | - | - | 22.10 |

Table S3. EXAFS fitting parameters for Fe foil, Fe_2_O_3_, Fe_3_O_4_, FePc, FePc-NCNT and FePc-Fe-NCNT.

| **Sample** | **Shell** | **CN^a^** | **R(Å)^b^** | **σ^2^(Å^2^)^c^** | **ΔE_0_(eV)^d^** | **R factor** |
| --- | --- | --- | --- | --- | --- | --- |
| Fe foil | Fe-Fe | 8* | 2.453 | 0.0044 | 4.313 | 0.009 |
| Fe_3_O_4_ | Fe-Fe | 6* | 2.835 | 0.0045 | 4.013 | 0.0013 |
|  | Fe-O | 12* | 3.518 | 0.017 | 11.010 |  |
|  | Fe-Fe | 12* | 3.501 | 0.013 | 10.227 |  |
|  | Fe-Fe | 4* | 3.656 | 0.011 | -8.186 |  |
| FePc-NCNT | Fe-N | 4.04 | 1.939 | 0.010 | 5.960 | 0.0014 |
| FePc-Fe-NCNT | Fe-N | 3.9 | 1.901 | 0.013 | -0.5834 | 0.003 |
|  | Fe-Fe | 1.1 | 2.581 | -0.004 | -5.646 |  |
| FePc | Fe-N | 4* | 1.915±0.019 | 0.0025 | 2.87 | 0.0018 |
| Fe_2_O_3_ | Fe-O | 3* | 0.0251 | 0.011 | 13.124 | 0.009 |
|  | Fe-O | 3* | 0.0387 | 0.0310 | 2.9763 |  |
|  | Fe-Fe | 1* | 0.1578 | 0.03112 | 16.798 |  |
|  | Fe-Fe | 3* | -0.00207 | 0.0105 | 16.428 |  |

Table S4. Summary of fitted ^57^Fe Mössbauer parameters and assignments of Fe species

| **Catalyst** | **Component** | **IS / mm****^–1^** | **QS / mm^–1^** | **Area / %** |
| --- | --- | --- | --- | --- |
| **FePc-NCNT** | D1 | 0.24 | 0.4 | 51.2 |
|  | D2 | 0.28 | 0.98 | 48.8 |
| **FePc-Fe-NCNT** | D1 | 0.09 | 0.66 | 25.1 |
|  | D2 | 0.08 | 3.25 | 74.9 |

Table S5. Comparison of TOF and MA between FePc-Fe-NCNT and other M-N-C materials.

| **Electrocatalysts** | **MA at 0.85 V (A/g)** | **TOF(s^–1^)** | **References** |
| --- | --- | --- | --- |
| FePc-Fe-NCNT | 990.91 | 0.64 | *This work* |
| FePc-NCNT | 446.67 | 0.52 | *This work* |
| Ti_4_N_3_O_x_/FePc | - | 0.142 | *Adv. Energy Mater. 2024, 2403899.* |
| Ti_4_N_3_Cl_x_/FePc | - | 0.288 | *Adv. Energy Mater. 2024, 2403899.* |
| 3D-G-PFePc | 81.88 | 0.93 | *Angew. Chem. Int. Ed. 2023, 62, e202301642.* |
| Pz-FeTPr | 63.6 | 0.57 | *Angew. Chem. Int. Ed. 2023, 62, e202308070.* |

Table S6. Comparison of ORR performance of FePc-Fe-NCNT at 1600 rpm in 0.1 M KOH with FePc-Fe-NCNT electrocatalysts in recent reported literatures.

| **Catalysts** | **E_1/2_ (V)** | **E_0_ (V)** | **References** |
| --- | --- | --- | --- |
| FePc-Fe-NCNT | 0.898 | 1.02 | **This work** |
| o-MQFe-10:20:5 | 0.861 | ~0.98 | *Angew. Chem., Int. Ed. 2022, 61, 202117617* |
| FePc/CoPc HS | 0.879 | 0.971 | *Adv. Funct. Mater. 2020, 30, 2005000* |
| Fe-ND/C | 0.79 | 0.91 | *Nano Res. 2021, 14, 1069-1077* |
| Fe SA-NSC-900 | 0.86 | 0.94 | *ACS Energy Lett. 2021, 6, 379-386* |
| Ti_4_N_3_O_x_/FePc | 0.87 | ~0.98 | *Adv. Energy Mater. 2024, 2403899* |
| FeSA/FeAC@PPy/CC | 0.83 | 0.93 | *Energy Environ. Sci., 2025, 18, 2839–2851* |
| Zn-N-C-1 | 0.873 | 0.96 | *Angew. Chem. Int. Ed., 2019, 58, 7035-7039* |
| Fe/CuSAs-Mag | 0.86 | 1.57 | *Angew. Chem. Int. Ed. 2023, 62 e2022304229* |
| H-3DOM-Co/ONC | 0.876 | 0.94 | *Adv. Mater. 2023, 35, 2301894* |
| Fe–N@Ni–HCFs | 0.88 | 0.935 | *Adv. Funct. Mater., 2022, 32, 2209273* |
| FePc NTs-rGO | 0.88 | 0.97 | *Proc. Natl. Acad. Sci. USA* ***2024****, 121, e2316553121* |
| FePc-DNA-rGO | 0.855 | 0.93 | *ACS Catal.* ***2024****, 14, 7514* |
| FePc@CNF | 0.875 | 0.966 | *Chem. Eng. J.* ***2024****, 483, 149243* |
| FePc-NPy-CNT | 0.923 | 1.011 | *Int. J. Hydrogen Energ. 101 (2025) 605–616* |
| FePc/CoPc HS | 0.879 | 0.971 | *Adv. Funct. Mater.* **2020**, 30, 2005000 |

Table S7. Comparison of liquid Zn-air battery performance between FePc-Fe-NCNT and previously reported electrocatalysts.

| **Electrocatalysts** | **Catalyst loading (mg cm^2^)** | **Power density (mW cm^2^)** | **Specific capacity**  **(mAh g ^−1^ )** | **References** |
| --- | --- | --- | --- | --- |
| **FePc-Fe-NCNT** | **1** | **186.9** | **788** | **This work** |
| Fe_ACs_/NPS | 1 | 172.4 | 863.5 | *Angew. Chem.Int. Ed.2023,62, e2023141* |
| Fe–N–C-2 | 2 | 218 | 810 | *Adv. Sci. 2023, 2305194* |
| FeN_3_O-O-Ti | 1 | 158 | 807.8 | *Angew. Chem. Int. Ed. 2022, 61, 61, e2021* |
| CR-Co/ClNC | 1 | 176.6 | 745 | *Nature Communications \| (2024) 15:1675* |
| Fe SACs HS | 1 | 170 | - | *Angew. Chem. Int. Ed. 2023, 62, e202304229* |
| Au SAC CoN@NF | 1 | 161.94 | 813.80 | *Adv. Funct. Mater.2024, 2316699* |
| Fe_SA/AC_@HNC | 1 | 171.5 | 811.8 | *Adv. Mater. 2024, 2400523* |
| CoSA/N, S-HCS | 1 | 173.1 | 781.1 | *Adv. Energy Mater., 2020, 2002896* |
| N-FeN_4_ | 1 | 171 | 772 | *Adv. Funct. Mater. 2024, 2409794* |
| CoFeN-NCNTs/CCM | 1 | 145 | 778 | *Adv. Funct. Mater., 2021, 2107608* |
| Co_2_P/CoN_4_@NSC-500 | 1 | 134.5 | - | *Angew.Chem.Int. Ed.2023*,62, e202216950 |

**References**

[1] B. Ravel and M. Newville, ATHENA, ARTEMIS, HEPHAESTUS: data analysis for X-ray absorption spectroscopy using IFEFFIT, J. Synchrotron. Radiat., 12 (2005) 537–541.

[2] Zabinsky, S. I.; Rehr, J. J.; Ankudinov, A.; Albers, R. C.; Eller, M. J. Multiple-Scattering Calculations of X-Ray-Absorption Spectra. Phys. Rev. B, 52 (1995) 2995−3009.

[3] O. Bunauand Y. Joly, Self-consistent aspects of x-ray absorption calculations, J. Phys.: Condens. Matter, 21 (2009) 345501.

[4] G. Kresse, J. Furthmüller, *Comput. Mater. Sci.* 1996, **6**, 15.

[5] P. E. Blochl, *Phys. Rev. B* 1994, **50**, 17953.

[6] G. Kresse, J. Furthmüller, *Phys. Rev. B* 1996, **54**, 11169.

[7] J. P. Perdew, K. Burke, M. Ernzerhof, *Phys. Rev. Lett.* 1996, **77**, 3865.

[8] S. Grimme, J. Antony, S. Ehrlich, H. Krieg, *J. Chem. Phys.* 2010, **132**, 154104.

[9] Y. Wang, Y.-J. Tang, K. Zhou, *J. Am. Chem. Soc.* 2019, **141**, 14115.

[10] R. Nelson, C. Ertural, J. George, V. L. Deringer, G. Hautier, R. Dronskowski, *J. Comput. Chem.* 2020, **41**, 1931.

[11] J. K. Nørskov, J. Rossmeisl, A. Logadottir, L. Lindqvist, J. R. Kitchin, T. Bligaard, H. Jonsson, *J. Phys. Chem. B* 2004, **108**, 17886.
